# Investigation of Novel Pesticides with Insecticidal and Antifungal Activities: Design, Synthesis and SAR Studies of Benzoylpyrimidinylurea Derivatives

**DOI:** 10.3390/molecules23092203

**Published:** 2018-08-31

**Authors:** Peiqi Chen, Xiangmin Song, Yongmei Fan, Weihao Kong, Hao Zhang, Ranfeng Sun

**Affiliations:** 1 Key Laboratory of Green Prevention and Control of Tropical Plant Diseases and Pests, Hainan University, Haikou 570228, China; chenpq17@lzu.edu.cn (P.C.); 18898958572@163.com (X.S.); yongmeifan@126.com (Y.F.); 15799032481@163.com (W.K.); 2 Institute of Tropical Agriculture and Forestry, Hainan University, Haikou 570228, China; 3 School of Chemical & Chemical Engineering, Inner Mongolia University, Hohhot 010021, China; haozhang@imu.edu.cn

**Keywords:** benzoylpyrimidinylurea derivatives, antifungal activity, insecticidal activity, embryo toxicity, zebrafish

## Abstract

In order to find pesticides with insecticidal and antifungal activities, a series of novel benzoyl pyrimidinylurea derivatives were designed and synthesized. All target compounds were identified by ^1^H-NMR spectroscopy and HRMS. Insecticidal and antifungal activity of these compounds were evaluated and the structure-activity relationships (SAR) were clearly and comprehensively illustrated. Compound **7**, with low toxicity to zebrafish (LC_50_ = 378.387 µg mL^−1^) showed 100% inhibition against mosquito (*Culex pipiens pallens*) at 0.25 µg mL^−1^. Both compounds **19** and **25** exhibited broad-spectrum fungicidal activity (>50% inhibitory activities against 13 phytopathogenic fungi), which were better than those of the commercial pesticide pyrimethanil (>50% inhibitory activities against eight phytopathogenic fungi). Furthermore, compounds **19** and **25** exhibited protective activity against *Sclerotinia sclerotiorum* on leaves of *Brassica oleracea* L. during in vivo experiments.

## 1. Introduction

**Figure 1:**
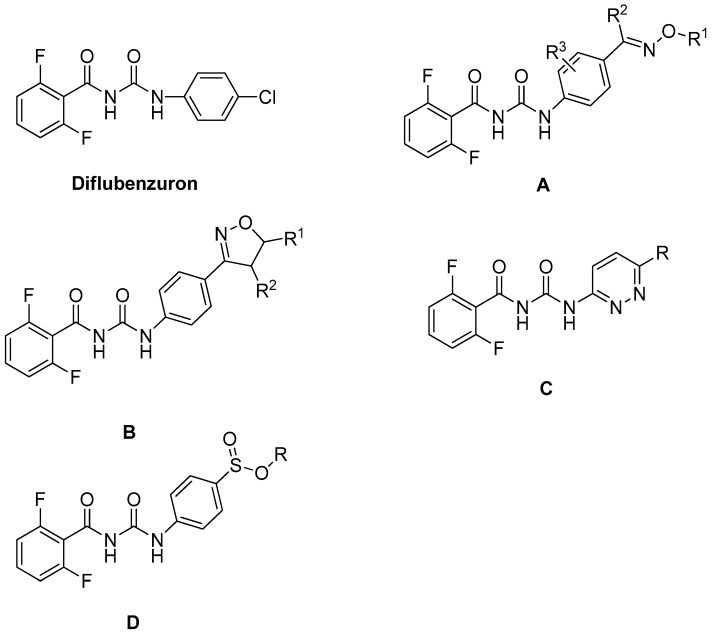
Chemical structures of benzoylurea compounds.

Plant disease and pest control are at least as old as agriculture because there is a need to keep crops free of pests and phythopatogenic fungi. Benzoylphenylureas (BPUs) have been developed as chitin synthesis inhibitors because of their unique mode of action coupled with a high degree of activity on targeted pests and low toxicity to non-target organisms [1,2]. The first commercial product Dimilin (diflubenzuron) (Figure 1), introduced in the market at 1975, exhibits broad spectrum larvicidal activities against caterpillars (*Pieris brassicae*), mosquitoes (*Aedes aegypti*), houseflies (*Musca domestica*), desert locust (*Schistocera gregaria*) and cotton strainers (*Dysdercus superstitiosus*) [3]. In the past few years, we and our co-workers have designed and synthesized several series of benzoylphenylureas (**A** and **B**, Figure 1) and most compounds exhibited excellent insecticidal activities against oriental armyworm (*Mythimna separata*) or mosquito (*Culex pipiens pallens*) [4,5,6,7,8]. Interestingly, compounds **C** and **D** (Figure 1) with a 2,2,2-trifluoroethyl group (R = CH_2_CF_3_) substituent, displayed much better insecticidal activities against oriental armyworm than other derivatives.

**Figure 2:**
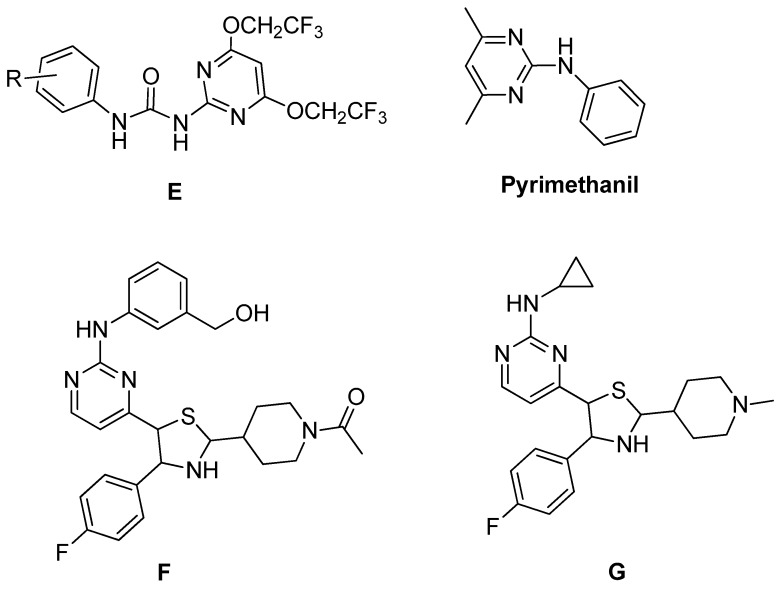
Chemical structures of compounds with pyrimidine groups.

At the same time, a class of phenylurea compounds (**E**, Figure 2) containing a pyrimidine ring attracted our attention. When bearing two 2,2,2-trifluoroethyl groups on the pyrimidine ring most of these compounds have good insecticidal activity performance against second-instar corn earworm (*Helicoverpa zea*) and beet armyworm (*Spodoptera exigua*) at 50 µg mL^−1^. 

More importantly, the 2-aminopyrimidinyl unit is commonly found in the chemical structure of antifungal compounds. For example, the antifungal activity of commercial pyrimethanil (Figure 2) against *Alternaria kikuchiana Tanaka* was 100% at 0.1 µg mL^−1^ [9,10,11,12]. Compound **F** (EC_50_ = 1.03 µg mL^−1^) exhibited better antifungal activity against *Phytophthora capsici* than dimethomorph (EC_50_ = 4.26 µg mL^−1^) (Figure 2) [13]. Compound **G** (Figure 2) with excellent antifungal activity (EC_50_ = 0.84 µg mL^−1^) against *Phytophthora capsici* was discovered in 2011 by Nam et al. [14].

**Figure 3:**
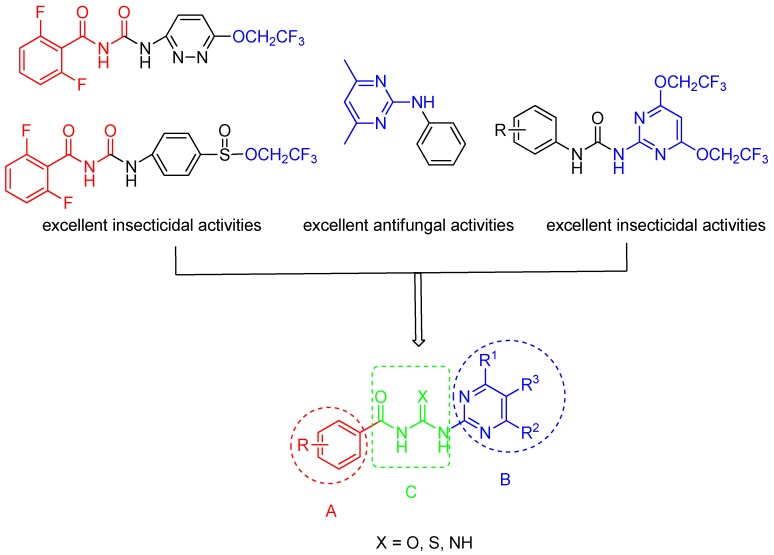
Design strategies of the target benzoylpyrimidinylurea derivatives.

Based on our previous works [4,5,6,7,8] and the literature [9,10,11,12,13,14], in order to find new pesticides with powerful insecticidal and antifungal properties, we were interested in introducing pyrimidine group into the benzoylurea backbone to design a series of benzoylpyrimidinylurea derivatives (Figure 3). Therefor target compounds **1**–**38** were synthesized and their insecticidal and antifungal activities were evaluated. At the same time, the aquatic toxicity of compounds **7** and **25** were evaluated by a zebrafish embryo acute toxicity test (OECD TG 236).

## 2. Results

### 2.1. Synthesis

**Scheme 1:**
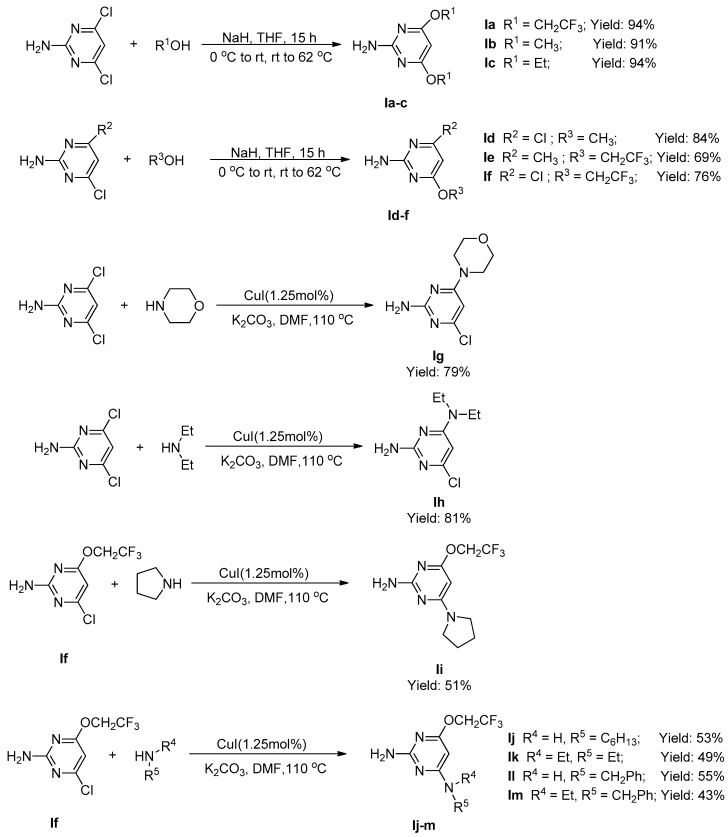
Synthetic route for substituted 2-aminopyrimidines **Ia**–**c**, **Id**–**f** and **Ig**–**m**.

**Scheme 2:**
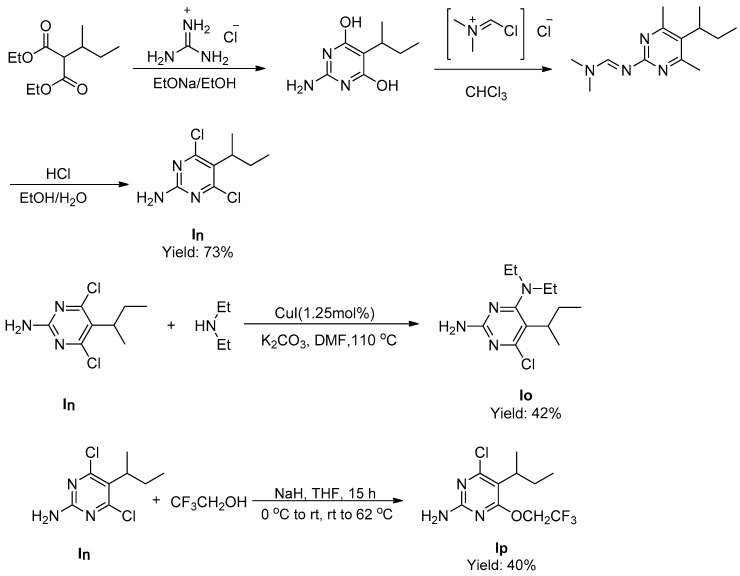
Synthetic route for substituted 2-aminopyrimidines **In**, **Io** and **Ip**.

In order to comprehensively analyze the structure-activity relationship of compounds, the chemical structures of the benzoylpyrimidinylureas was separated into three units, namely, the benzoyl ring (**A**), pyrimidine ring (**B**) and urea bridge (**C**) (Figure 3). Substituted 2-aminopyrimidines **Ia**–**p** were synthesized and used as starting materials for the synthesis of the target compounds (Scheme 1 and Scheme 2).

**Scheme 3:**
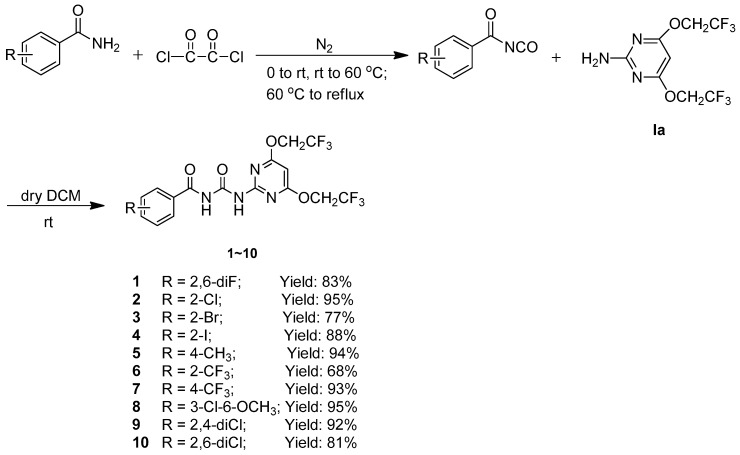
Synthetic route for compounds **1**–**10**.

In a first series of compounds, intermediate **Ia** was retained and the benzoyl moiety was altered resulting in compounds **1** to **10** (Scheme 3).

**Scheme 4:**
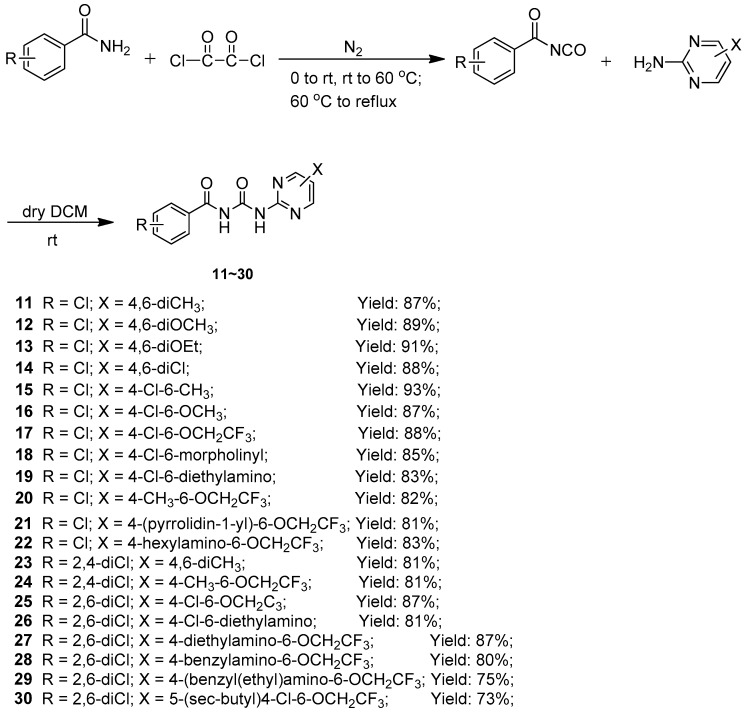
Synthetic route for compounds **11**–**30**.

Secondly, different substituents were changed on the pyrimidine ring and the compounds **11**–**30** with substituted 2-aminopyrimidines were obtained (Scheme 4).

**Scheme 5:**
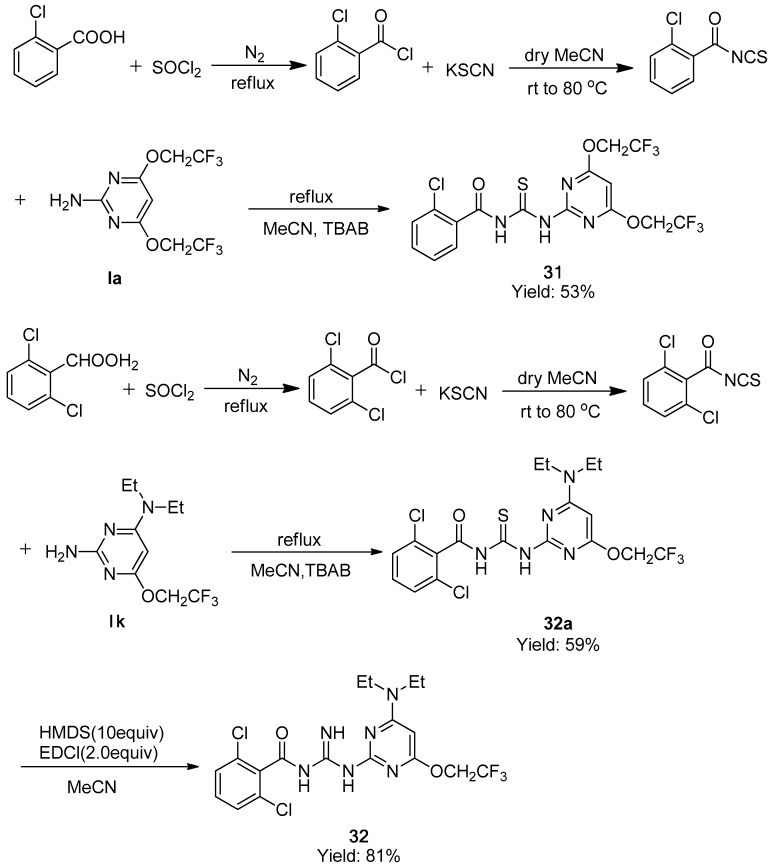
Synthetic route for compounds **31** and **32**.

Thirdly, we replaced the oxygen atom on the urea bridge with sulfur or nitrogen to gain compounds **31** and **32** (Scheme 5).

**Scheme 6:**
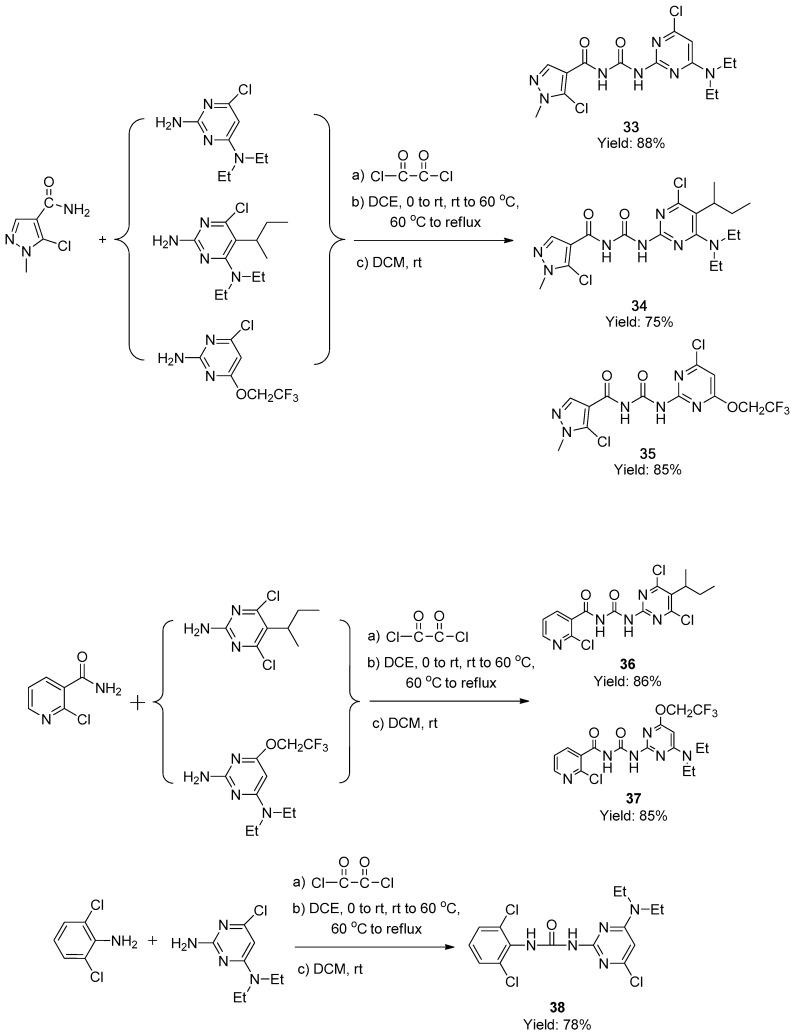
Synthetic route for compounds **33**–**38**.

Finally, the benzoyl ring was exchanged by a six- or five-membered nitrogen heterocycles to compare the effects between benzene ring and heterocycles on the bioactivity of the compounds, therefor compounds **33**–**37** were synthesized (Scheme 6). To illustrate the role of the carbonyl group of the benzoyl group in the biological activity of the target compounds, compound **38** was synthesized from 2,6-dichloroaniline (Scheme 6). All reactions gave excellent yields and the NMR data of compounds proved the proposed structures. Among them, compounds with trifluoromethyl groups have a characteristic quartet peak with ^13^C-^19^F couplings in the NMR spectra. The spectra are shown in Figures S1–S101 in the Supporting Information.

### 2.2. Bioassays

#### 2.2.1. Toxicity against Mosquito (*Culex pipiens pallens*)

Table 1 shows the larvicidal activities of the compounds **1**–**25**, **31**, **37** and fipronil against mosquito. The bioassay results indicated that eighteen compounds (**1**–**7**, **9**–**10**, **13**–**14**, **16**, **18**, **20**, **22**–**24**, **31**) exhibited more than 50% larvicidal activities against mosquito and eleven of them reached 100% at 10 µg mL^−1^, especially, compound **7** which showed 100% mortality even at 0.25 µg mL^−1^. By comparing the chemical structure of compounds **2** (2-Cl), **3** (2-Br) and **4** (2-I), we found that the larvicidal activitives decreased (100%, 60% mortality at 2 µg mL^−1^, 50% mortality at 10 µg mL^−1^ respectively) as the radius of the halogen atom at the 2-position on the benzoyl ring increased. Next, we found that when the 2,6-position on the benzoyl ring was substituted by two halogens or electron-rich groups, it was not conducive to larvicidal activity, for example, the larva mortalities of compounds **8** (2-OCH_3_), **9** (2,4-di-Cl) and **10** (2,6-di-Cl) were 20% at 10 µg mL^−1^, 40% at 2 µg mL^−1^ and 20% at 5 µg mL^−1^ respectively. 

However, electron-withdrawing groups in the *para* position enhanced the bioactivity of the compounds, for example, the larva mortality of compound **7** (4-CF_3_) reached 100% at 0.25 µg mL^−1^. When the 4,6-position on the pyrimidine ring were substituted with haloalkoxyl, alkoxy or alkyl groups, the larvicidal activity decreased successively, at the same time the longer the alkoxy chain, the larvicidal activity was better. For example, the mortalities of compounds **11** (4,6-di-CH_3_), **12** (4,6-di-OCH_3_), **13** (4,6-di-OEt) and **2** (4,6-di-OCH_2_CF_3_) were 10% at 10 µg mL^−1^, 20% at 10 µg mL^−1^, 70% at 2 µg mL^−1^, 100% at 0.5 µg mL^−1^, respectively. Similarly, the structure-activity relationships of compounds **2** and **14** told us that a trifluoroethoxy group was more helpful to increase the mortality activities than s halogen group. Furthormore it was easy to find that the urea bridge unit was important to the larvicidal activity. For example compound **31** containing a thiourea unit (50% mortality at 1 µg mL^−1^) exhibited lower larvicidal activity than compound **2** (100% mortality at 0.5 µg mL^−1^). Finally, when a pyridine ring (compound **37**) was employed instead of a benzene ring (compound **2**), the larvicidal activities were reduced greatly (15% mortality of compound **37** at 10 µg mL^−1^ and 100% mortality of compound **2** at 0.5 µg mL^−1^). Therefore, it was concluded that electron-withdrawing groups (such as a trifuloromethyl group) on the *para* position of the benzoyl ring, alkoxy groups (especially haloalkoxyl groups, such as a trifluoroethoxy group) and a urea bridge unit favored the larvicidal activities.

#### 2.2.2. Stomach Toxicity against Oriental Armyworm (*Mythimna separata*)

The bioactivity results indicated that most compounds exhibited certain insecticidal activities against oriental armyworm as listed in Table 1. Firstly, we can easily see from compounds **2** (2-Cl) and **5** (4-Cl) or **6** (2-CF_3_) and **7** (4-CF_3_) and their larva moralities (5%, 65%, 15% and 65% at 600 µg mL^−1^ respectively) that *para*-substituents of the benzoyl ring result in better insecticidal activities than *ortho*-substituents. Secondly, it was figured out that both the radius and electronegativity of the halogen atom at the 2-position on the benzoyl ring influenced the larvicidal activities, for example, compound **3** (2-Br, 100% mortality at 200 µg mL^−1^) was a more effective insecticidal agent than compound **4** (2-I, 70% mortality at 600 µg mL^−1^) which exhibited much better larvicidal activity than compound **2** (2-Cl, 5% mortality at 600 µg mL^−1^). At the same time, it also pointed out that chlorine atom on the pyrimidine ring was beneficial to improve the insecticidal activity of compounds, for example, the larva mortalities of compounds **14** (4,6-Cl), **15** (4-Cl, 6-CH_3_) and **16** (4-Cl, 6-OCH_3_) were 50%, 30%, 5% at 600 µg mL^−1^ respectively. Interestingly, when we replaced the oxygen atom on the urea bridge with a sulfur atom, the insecticidal activities of the compounds increased greatly, as seen by comparing the insecticidal activities of compound **2** (5% mortality of at 600 µg mL^−1^) and compound **31** (100% mortality at 600 µg mL^−1^).

#### 2.2.3. In Vitro Antifungal Activity

The fungicidal results are listed in Table 2. Most of the derivatives showed antifungal activity against 14 kinds of phytopathogenic fungi. We first determined the antifungal activities of compounds **2** (2-Cl), **3** (2-Br), **4** (2-I), **5** (4-Cl), **6** (2-CF_3_), **7** (4-CF_3_) and **8** (2-OCH_3_, 5-Cl) and we found that *ortho*-halogen substitutents on the benzoyl ring were beneficial for antifungal activity. Among them, compound **2** was better than the others. At this moment, our design strategy was divided into two parts. One was to maintain 2-chloro group on the benzoyl ring and a series of differently substituted compounds (such as compounds **11**–**22**) at the 4,6-position on the pyrimidine ring were synthesized. The other was that we continued to explore the effect of the number of halogen atoms on the benzoyl ring for the antifungal activity, followed by the synthesis of compounds with different substitutions at the 4,6-position on pyrimidine ring, such as compounds **1**, **9**, **10** and **23**–**30**.

From the first part of strategy, we reached three important conclusions. Firstly, compounds with alkoxy groups had better antifungal activity than those bearing an alkyl group, and if the hydrogen atom on the alkoxy group was substituted with a halogen atom, the antifungal activity of compounds would be further enhanced. For example, the antifungal activity order of compounds **2**, **11**, **12** and **13** was compound **2** (4,6-di-OCH_2_CF_3_) > compound **13** (4,6-di-OEt) > compound **12** (4,6-di-OCH_3_) > compound **11** (4,6-di-CH_3_) (Table 2). Secondly, chlorine atoms were beneficial to increase the antifungal activity of the compounds. For example, the antifungal activity order of compounds **14**, **15** and **16** was compound **14** (4,6-Cl) > compound **15** (4-Cl, 6-CH_3_) > compound **16** (4-Cl, 6-OCH_3_) (Table 2).

Thirdly, when the 4,6-positions of the pyrimidine ring were substituted by a chlorine atom and trifluoroethoxy group (such as compound **17**), the antifungal activity was weakened instead of enhanced. Therefore, we further fixed the 4-position of the pyrimidine ring with a chlorine atom or trifluoroethoxy group, and a series of compounds with nitrogen-substituted group at 6-position of the pyrimidine ring were synthesized, such as compounds **18** (4-Cl, 6-morpholine), **19** (4-Cl, 6-*N*’,*N*’-diethyl), **21** (4-OCH_2_CF_3_, 6-pyrrolidinyl) and **22** (4-OCH_2_CF_3_, 6-*N*’-hexyl) (Table 2). Fortunately, compound **19** exhibited broad-spectrum fungicidal activity (>60% inhibitory activities against 13 phytopathogenic fungi), which was better than the inhibitory activities of commercial pyrimethanil (>50% against eight phytopathogenic fungi, Table 2).

As another part of our strategy, we found that the antifungal activity of **10** (2,6-Cl on the benzoyl ring) is superior to compounds **9** (2,4-Cl on the benzoyl ring, Table 2). Compound **25** exhibited broad-spectrum fungicidal activity (>50% inhibitory activities against 13 phytopathogenic fungi). Especially notable was the inhibition activity of compound **25** against *Rhizoctonia cerealis*, *Phytophthora capsica*, *Botrytis cinereal* and *Physalospora piricola.* reached 94.9%, 90.7%, 89.7% and 84.2% at 50 µg mL^−1^ respectively, which was comparable to commercial pyrimethanil (Table 2). Unlike the low activity of compound **17** (2-Cl), the broad-spectrum antifungal activity of compound **25** indicated that 2,6-dichloro substitution on the benzoyl ring was very important for the antifungal activity. However, when we replaced the ethyl group on the nitrogen with benzyl group (compound **29** compared with compound **27**) or the 5-position of the pyrimidine ring was substituted with 2-butyl group (compound **30**), the antifungal activity decreased significantly (Table 2). This result indicated that groups with large steric hindrance were not conducive to antifungal activity.

In the investigation of the urea bridge unit, a benzoylthiourea derivative (compound **31**) and a benzoylguanidine derivative (compound **32**) displayed lower antifungal activities than compound **2** and compound **27**, respectively (Table 2). Although the phenylurea compound **38** also showed better antifungal activity (>50% inhibitory activities against 10 phytopathogenic fungi), its antifungal activity was lower and its antifungal spectrum smaller than those of compound **19**. Finally, because of the broad spectrum and powerful antifungal activity of compounds **19** and **25**, the values of EC_50_ for compounds **19** and **25** against *Sclerotinia sclerotiorum*, *Botrytis cinereal*, *Physalospora piricola*, *Rhizoctonia cerealis* were determined and are listed in Table 3.

#### 2.2.4. In Vivo Antifungal Bioassay against *Sclerotinia sclerotiorum*

The protective effets of compounds **19** and **25** and carbendazim (positive control) against *S. sclerotiorum* on detached *Brassica oleracea* L. leaves were evaluated. The results showed that both compounds **19** and **25** exhibited protective activity on leaves of *Brassica oleracea* L. (Table 4). 

This protective effect was enhanced with the increasing concentration of the compounds (experimental photos shown in Figure S102 in the Supporting Information). For example, the values of the protective effect for compound **19** were 55.7% at 3 mg mL^−1^, 35.2% at 1.5 mg mL^−1^, 34.1% at 0.5 mg mL^−1^ and the protective effect values for compound **25** were 83.0% at 3 mg mL^−1^, 35.2% at 1.5 mg mL^−1^, 20.5% at 0.5 mg mL^−1^ (Table 4). Interestingly, although compound **19** has better in vitro antifungal activity than compound **25**, it appears that compound **25** has a better protective effect.

#### 2.2.5. Zebrafish Embryo Toxicity Assay

The fish embryo acute toxicity test (FET) with zebrafish embryos, which is a general model in ecotoxicology and toxicology had been established in our labs [15,16]. The mortality in the positive control was 40% greater than 30% and survival in the negative control was 100%. As shown in Table 5, compound **7** has low toxicity to zebrafish embryos (LC_50_ = 378.387 μg mL^−1^), which was what we like to see because of its excellent larvicidal activity to mosquito. At the same time, compound **25** which exhibited broad-spectrum antifungal activity also possesses low toxicity to zebrafish embryos (LC_50_ = 21.668 μg mL^−1^). Fortunately, both compounds **7** and **25** did not affect zebrafish embryo hatching, induce the pericardial cysts and produce abnormality (such as malformation and coagulation) during the zebrafish embryo acute toxicity test.

## 3. Materials and Methods 

### 3.1. Instruments

^1^H-NMR and ^13^C-NMR spectra were obtained at 500/126 MHz using a Bruker Avance III 500 spectrometer (Bruker Daltonics, Bremen, Germany) in CDCl_3_ or DMSO-*d*_6_ solution with tetramethylsilane as the internal standard. Chemical shift values (*δ*) are given in ppm. HRMS data were obtained on FTICR-MS (Ionspec 7.0 T, Lebrilla League, Davis, CA, USA) high resolution mass spectrometer and LQC Advantage MAX multi-stage ion mass spectrometer (Agilent Technologies Inc., CA, USA). The melting points were determined on an X-4 binocular microscope melting point apparatus (Beijing Tech Instruments Co., Beijing, China) and are uncorrected. Yields were not optimized.

### 3.2. General Synthesis

All anhydrous solvents were dried and purified by standard techniques. Chemicals (analytical grade) were purchased from Aladdin (Shanghai, China).

### 3.3. General Synthesis Procedure for Substituted 2-Aminopyrimidines ***Ia***–***c***

Intermediates **Ia**–**c** were prepared according to the literature [17]. A solution of NaH (0.35 g, 14.6 mmol) in dry THF (12 mL) was cooled to 0 °C under N_2_, then a substituted alcohol (15.2 mmol) was added dropwise. The mixture was stirred for 15 min while maintaining the temperature at 0 °C. Next, 2-amino-4,6-dichloropyrimidine (1.0 g, 6.1 mmol) was added to the solution. The reaction was continued at 62 °C for 15 h. Then the mixture was cooled to ambient temperature, and quenched with 1 mL of 1 M hydrochloric acid solution. The mixture was diluted with EtOAc (20 mL), washed twice with a saturated NaHCO_3_ solution (20 mL) and brine (20 mL), dried with anhydrous Na_2_SO_4_ and evaporated in vacuo. Finally, the residue was purified by silica gel column chromatography (EtOAc/petroleum ether) to afford compounds **Ia**–**c**.

*4,6-Bis(2,2,2-trifluoroethoxy)pyrimidin-2-amine* (**Ia**). Yellow oil, yield = 94%. ^1^H-NMR (CDCl_3_) δ 5.58 (s, 1H, ArH), 4.96 (s, 2H, NH_2_), 4.57 (q, *J* = 8.6 Hz, 4H, OCH_2_CF_3_).

*4,6-Dimethoxypyrimidin-2-amine* (**Ib**). White solid, mp 87–89 °C, yield = 91%. ^1^H-NMR (CDCl_3_) δ 5.46 (s, 1H, ArH), 5.09 (s, 2H, NH_2_), 3.84 (s, 6H, OCH_3_).

*4,6-Diethoxypyrimidin-2-amine* (**Ic**). White solid, mp 174–176 °C, yield = 94%. ^1^H-NMR (CDCl_3_) δ 5.42 (s, 1H, ArH), 4.95 (s, 2H, NH_2_), 4.23 (q, *J* = 7.1 Hz, 4H, OCH_2_CH_3_), 1.35 (t, *J* = 7.1 Hz, 6H, OCH_2_CH_3_).

### 3.4. General Synthesis Procedure for Substituted 2-Aminopyrimidines ***Id***–***f***

Intermediates **Id**–**f** were prepared by a method similar to that for intermediates **Ia**–**c**. A solution of NaH (5.8 mmol) in dry THF (12 mL) was cooled to 0 °C under N_2_, then a substituted alcohol (6.1 mmol) was added dropwise. The mixture was stirred for 15 min while maintaining the temperature at 0 °C. Next, 2-amino-4-chloro-6- substituted-pyrimidine (5.8 mmol) was added to the solution. The reaction was continued at 62 °C for 15 h. Then the mixture was cooled to ambient temperature, and quenched with 1 mL of 1 M hydrochloric acid solution. The mixture was diluted with EtOAc (20 mL), washed twice with a saturated NaHCO_3_ solution (20 mL) and brine (20 mL), dried with anhydrous Na_2_SO_4_ and evaporated in vacuo. Finally, the residue was purified by silica gel column chromatography (EtOAc/petroleum ether) to afford compounds **Id**–**f**.

*4-Chloro-6-methoxypyrimidin-2-amine* (**Id**). White solid, mp 165–167 °C, yield = 84%. ^1^H-NMR (CDCl_3_) δ 6.04 (s, 1H, ArH), 5.35–5.14 (m, 2H, NH_2_), 3.81 (s, 3H, OCH_3_).

*4-Methyl-6-(2,2,2-trifluoroethoxy)pyrimidin-2-amine* (**Ie**). White solid, mp 109–111 °C, yield = 69%. ^1^H-NMR (CDCl_3_) δ 6.42 (s, 1H, ArH), 5.50 (s, 2H, NH_2_), 4.60 (q, *J* = 8.4 Hz, 2H, OCH_2_CF_3_), 2.18 (s, 3H, CH_3_).

*4-Chloro-6-(2,2,2-trifluoroethoxy)pyrimidin-2-amine* (**If**). White solid, mp 82–83 °C, yield = 76%. ^1^H-NMR (CDCl_3_) δ 6.23 (s, 1H, ArH), 5.59 (s, 2H, NH_2_), 4.70 (q, *J* = 8.4 Hz, 2H, OCH_2_CF_3_).

### 3.5. General Synthesis Procedure for Substituted 2-Aminopyrimidines ***Ig***–***m***

Intermediates **Ig**–**m** were prepared according to [18]. A suspension of CuI (5.8 mg, 0.03 mmol), K_2_CO_3_ (0.41 g, 2.9 mmol), the corresponding amine (0.21 g, 2.4 mmol) and substituted 2-aminopyrimidines (0.4 g, 2.4 mmol) in 2 mL DMF was stirred for 30 min at room temperature and then the mixture was heated to 110 °C. At the end of the reaction, as monitored by TLC, the reaction mixture was cooled to room temperature and diluted with 20 mL of EtOAc, washed twice with a saturated NaHCO_3_ solution (20 mL) and brine (20 mL), dried with anhydrous MgSO_4_ and evaporated in vacuo. Finally, the residue was purified by silica gel column chromatography to afford compound **Ig**–**m**.

*4-Chloro-6-morpholinopyrimidin-2-amine* (**Ig**). White solid, mp 210–212 °C, yield = 79%. ^1^H-NMR (CDCl_3_) δ 5.94 (s, 1H, ArH), 5.00 (s, 2H, NH_2_), 3.79–3.68 (m, 4H, morpholine), 3.55 (t, *J* = 4.9 Hz, 4H, morpholine).

*6-Chloro-N^4^,N^4^-diethylpyrimidine-2,4-diamine* (**Ih**). White solid, mp 104–106 °C, yield = 81%. ^1^H-NMR (CDCl_3_) δ 5.84 (s, 1H, ArH), 5.04 (s, 2H, NH_2_), 3.42 (s, 4H, CH_2_CH_3_), 1.15 (t, *J* = 7.1 Hz, 6H, CH_2_CH_3_).

*N^4^-(Pyrrolidin-1-yl)-6-(2,2,2-trifluoroethoxy)pyrimidine-2,4-diamine* (**Ii**). White solid, mp 118–120 °C, yield = 51%. ^1^H-NMR (CDCl_3_) δ 5.21 (s, 1H, ArH), 4.82 (s, 2H, NH_2_), 4.67 (q, *J* = 8.7 Hz, 2H, OCH_2_CF_3_), 3.38 (s, 4H, tetrahydropyrrole), 1.93 (d, *J* = 5.9 Hz, 4H, tetrahydropyrrole). ^13^C-NMR (CDCl_3_) δ 168.7, 163.3, 162.0, 124.9, 122.7, 62.0, 61.7, 61.4, 61.1, 46.5, 25.3. HRMS (ESI): *m*/*z* calcd for C_10_H_13_F_3_N_4_O [M + H]^+^: 263.1120, found 263.1136.

*N^4^-Hexyl-6-(2,2,2-trifluoroethoxy)pyrimidine-2,4-diamine* (**Ij**). Yellow oil, yield = 53%. ^1^H-NMR (CDCl_3_) δ 5.24 (s, 1H, ArH), 5.13 (s, 1H, NH), 5.06 (s, 2H, NH_2_), 4.65 (q, *J* = 8.6 Hz, 2H, OCH_2_CF_3_), 3.12 (q, *J* = 6.7 Hz, 2H, N–CH_2_(CH_2_)_4_CH_3_), 1.77–1.42 (m, 2H, N–(CH_2_)_4_CH_2_CH_3_), 1.44–1.11 (m, 6H, N–CH_2_(CH_2_)_3_CH_2_CH_3_), 0.88 (t, *J* = 6.8 Hz, 3H, N–(CH_2_)_5_CH_3_). ^13^C-NMR (CDCl_3_) δ 169.4, 165.6, 162.1, 124.7, 122.5, 62.0, 61.7, 61.5, 61.2, 41.8, 31.5, 29.2, 26.6, 22.6, 14.0. HRMS (ESI): *m*/*z* calcd for C_10_H_19_F_3_N_4_O [M + H]^+^: 293.1589, found 293.1593.

*N^4^,N^4^-Diethyl-6-(2,2,2-trifluoroethoxy)pyrimidine-2,4-diamine* (**Ik**). Yellow oil, yield = 49%. ^1^H-NMR (CDCl_3_) δ 5.32 (s, 1H, ArH), 4.78 (s, 2H, NH_2_), 4.72–4.61 (m, 2H, OCH_2_CF_3_), 3.41 (q, *J* = 7.0 Hz, 4H, CH_2_CH_3_), 1.13 (t, *J* = 7.1 Hz, 6H, CH_2_CH_3_). ^13^C-NMR (CDCl_3_) δ 170.4, 169.3, 163.9, 124.9, 81.7, 62.3, 62.0, 61.7, 61.4, 42.1, 12.9. HRMS (ESI): *m*/*z* calcd for C_10_H_15_F_3_N_4_O [M + H]^+^: 265.1276, found 265.1280.

*N^4^-Benzyl-6-(2,2,2-trifluoroethoxy)pyrimidine-2,4-diamine* (**Il**). Yellow oil, yield = 55%. ^1^H-NMR (CDCl_3_) δ 7.36–7.15 (m, 5H, Ph), 5.66 (s, 1H, ArH), 5.22 (s, 1H, NH), 5.02 (s, 2H, NH_2_), 4.59 (q, *J* = 8.6 Hz, 2H, OCH_2_CF_3_), 4.31 (d, *J* = 6.0 Hz, 2H, PhCH_2_). ^13^C-NMR (CDCl_3_) δ 169.4, 165.6, 162.2, 138.2, 128.7, 127.5, 127.1, 126.9, 124.7, 122.5, 120.3, 62.0, 61.7, 61.4, 61.2, 45.6. HRMS (ESI): *m*/*z* calcd for C_13_H_13_F_3_N_4_O [M + H]^+^: 299.1120, found 299.1119.

*N^4^-Benzyl-N^4^-ethyl-6-(2,2,2-trifluoroethoxy)pyrimidine-2,4-diamine* (**Im**). Yellow oil, yield = 43%. ^1^H-NMR (DMSO-*d*_6_) δ 7.65–7.03 (m, 5H, Ph), 6.23 (s, 2H, NH_2_), 5.31 (s, 1H, ArH), 4.86 (q, *J* = 9.2 Hz, 2H, OCH_2_CF_3_), 4.69 (s, 2H, PhCH_2_), 3.41 (s, 2H, CH_2_CH_3_), 1.04 (t, *J* = 7.0 Hz, 3H, CH_2_CH_3_). ^13^C-NMR (DMSO-*d*_6_) δ 169.6, 165.1, 163.1, 129.4, 127.9, 127.7, 126.1, 123.9, 75.6, 61.3, 61.1, 42.7, 13.4. HRMS (ESI): *m*/*z* calcd for C_15_H_17_F_3_N_4_O [M + H]^+^: 327.1433, found 327.1429.

### 3.6. Synthesis of 5-(sec-Butyl)-4,6-dichloropyrimidin-2-amine (Intermediate ***In***)

Intermediate **In** was prepared according to [19]. Elemental sodium (0.30 g, 13.0 mmol) was added into absolute ethanol (7 mL) under N_2_ while being intensively stirred with a magnetic stirrer. After all the sodium was dissolved and the reaction mixture was cooled to room temperature, guanidine hydrochloride (0.49 g, 5.1 mmol) was added under intensive stirring, followed by the diethyl *sec*-butylmalonate (1.9 g, 4.6 mmol). The reaction mixture was further stirred intensively at room temperature. After another 4 h, absolute ethanol (5 mL) was added and the reaction mixture was refluxed for 1 h. Afterward, ethanol was evaporated on a vacuum rotary evaporator and water (12 mL) was added to the reaction mixture. After stirring, the product was dissolved. The obtained mixture was subsequently neutralized by acetic acid and then this mixture was heated under reflux for 10 min and then cooled to room temperature. This heating and cooling was repeated twice to get a well-filterable solid. The solid was filtered off, washed with water (2 × 50 mL), ethanol (2 × 50 mL), and acetone (2 × 50 mL). The product was dried under high vacuum for 2 days. Subsequently, the aboveobtained solid (4.6 mmol) was suspended under N_2_ in a solution of (chloro-methylene)dimethyl ammonium chloride (4.8 g, 37.1 mmol) in chloroform (19 mL). The reaction mixture was subsequently heated at reflux for 4 h, during which the starting material was completely dissolved. The reaction mixture was cooled to the room temperature, poured into ice and rapidly neutralized with a saturated aqueous NaHCO_3_ solution. The obtained mixture was quickly transferred into a separatory funnel and immediately extracted with chloroform (3 × 20 mL). The organic layers were combined together, dried over MgSO_4_, filtered and concentrated down on a rotary evaporator. This crude residue was dissolved in the mixture of ethanol (9 mL) and 37% aqueous HCl (0.9 mL). The reaction mixture was heated at 50 °C for 2 h. After that, water (14 mL) was added and the reaction mixture was stirred for 10 min. The precipitated product was filtered off and washed with a water/ethanol mixture (*v*/*v* = 1/1, 2 × 5 mL), 5 % aqueous solution of NaHCO_3_ (5 mL). The product was subsequently recrystallized from aqueous ethanol, filtered off, washed with a water/ethanol mixture (*v*/*v* = 1/1, 5 mL) and the solid recrystallized from ethanol to give intermediate **In** as a white solid, mp 157–159 °C, yield = 73%. ^1^H-NMR (CDCl_3_) δ 5.98 (d, *J* = 6.5 Hz, 2H, NH_2_), 3.27 (dq, *J* = 6.9, 3.7, 2.9 Hz, 1H, CH), 1.95–1.75 (m, 1H, CHCH_2_), 1.63 (dddd, *J* = 16.0, 9.0, 4.7, 2.3 Hz, 1H, CHCH_2_), 1.24 (dt, *J* = 7.4, 2.3 Hz, 3H, CHCH_3_), 0.77 (ddd, *J* = 9.1, 5.7, 2.1 Hz, 3H, CHCH_2_CH_3_).

### 3.7. Synthesis of 5-(sec-Butyl)-6-chloro-N^4^,N^4^-diethylpyrimidine-2,4-diamine (Intermediate ***Io***)

Intermediate Io was prepared by a method similar to that used for compounds **Ig**–**m**. Yellow oil, yield = 42%. ^1^H-NMR (DMSO-*d*_6_) δ 6.44 (s, 2H), 3.23 (dd, *J* = 13.7, 6.9 Hz, 2H), 3.05 (dd, *J* = 13.7, 7.0 Hz, 2H), 2.79 (q, *J* = 7.4 Hz, 1H), 1.79–1.61 (m, 2H), 1.31 (d, *J* = 7.1 Hz, 3H), 1.05 (t, *J* = 7.0 Hz, 6H), 0.71 (t, *J* = 7.4 Hz, 3H). ^13^C-NMR (DMSO-*d*_6_) δ 170.8, 160.8, 159.7, 114.1, 45.8, 34.4, 28.3, 19.0, 14.0, 13.6. HRMS (ESI): *m*/*z* calcd for C_12_H_21_ClN_4_ [M + Na]^+^: 279.1352, found 279.1356.

### 3.8. Synthesis of 5-(sec-Butyl)-4-chloro-6-(2,2,2-trifluoroethoxy)pyrimidin-2-amine (Intermediate ***Ip***)

Intermediate **Ip** was prepared by a method similar to that used for compounds **Id**–**f**. White solid, mp 53–54 °C, yield = 40%. ^1^H-NMR (DMSO-*d*_6_) δ 7.00 (s, 2H, NH2), 4.98 (q, *J* = 9.0 Hz, 2H, OCH_2_CF_3_), 3.02 (dt, *J* = 9.3, 6.7 Hz, 1H, CH), 1.76–1.62 (m, 1H, CHCH_2_), 1.56 (ddd, *J* = 13.6, 7.6, 6.4 Hz, 1H, CHCH_2_), 1.18 (d, *J* = 7.1 Hz, 3H, CHCH_3_), 0.75 (t, *J* = 7.4 Hz, 3H, CHCH_2_CH_3_). ^13^C-NMR (DMSO-*d*_6_) δ 167.6, 160.9, 160.7, 109.8, 62.8, 62.5, 62.2, 62.0, 36.5, 27.9, 19.4, 13.3. HRMS (ESI): *m*/*z* calcd for C_10_H_13_ClF_3_N_3_O [M + H]^+^: 284.0777, found 284.0799.

### 3.9. General Synthesis Procedure for Target Compounds ***1***–***30***

Compounds **1**–**30** were synthesized according to [20,21]. Substituted benzamide (5.0 mmol) and 1,2-dichloroethane (10 mL) were added to a 100 mL three-necked flask under N_2_. The reaction mixture was cooled to 0 °C and oxalyl chloride (1.27 g, 10.0 mmol) was added dropwise under stirring, then the mixture was stirred at room temperature for 1 h, left standing at 60 ° C for 3 h and refluxed for 1 h. The solvent and excess oxalyl chloride were evaporated under reduced pressure to give a yellow transparent liquid. Anhydrous dichloromethane (5 mL) was added to the residue, then a substituted 2-aminopyrimidine (2.5 mmol) was added to the system and reacted at room temperature for 12 h. The solvent was removed under reduced pressure and the residue was purified by flash column chromatography on silica gel to give compounds **1**–**30**.

*N-((4,6-bis(2,2,2-Trifluoroethoxy)pyrimidin-2-yl)carbamoyl)-2,6-difluorobenzamide* (**1**). White solid, mp 163–164 °C, yield = 83%. ^1^H-NMR (DMSO-*d*_6_) δ 11.65 (s, 1H, NH), 10.77 (s, 1H, NH), 7.76–7.57 (m, 1H, Ph), 7.26 (t, *J* = 8.3 Hz, 2H, Ph), 6.40 (s, 1H, ArH), 5.07 (q, *J* = 8.9 Hz, 4H, OCH_2_CF_3_). ^13^C-NMR (DMSO-*d*_6_) δ 170.9, 160.6, 156.3, 148.9, 134.2, 125.6, 123.4, 113.2, 113.0, 86.8, 63.7, 63.4, 63.1, 62.8. HRMS (ESI): *m*/*z* calcd for C_16_H_10_F_8_N_4_O_4_ [M + H]^+^: 475.0652, found 475.0641.

*N-((4,6-bis(2,2,2-Trifluoroethoxy)pyrimidin-2-yl)carbamoyl)-2-chlorobenzamide* (**2**). White solid, mp 173–175 °C, yield = 95%. ^1^H-NMR (DMSO-*d*_6_) δ 11.47 (s, 1H, NH), 10.96 (s, 1H, NH), 7.65 (dd, *J* = 7.6, 1.6 Hz, 1H, Ph), 7.61–7.52 (m, 2H, Ph), 7.48 (td, *J* = 7.3, 1.7 Hz, 1H, Ph), 6.39 (s, 1H, ArH), 5.07 (q, *J* = 8.9 Hz, 4H, OCH_2_CF_3_). ^13^C-NMR (DMSO-*d*_6_) δ 170.9, 169.2, 156.3, 149.0, 135.6, 133.1, 130.7, 130.0, 128.2, 125.7, 123.4, 86.8, 63.6, 63.3, 63.0, 62.8. HRMS (ESI): *m*/*z* calcd for C_16_H_11_ClF_6_N_4_O_4_ [M + H]^+^: 473.0451, found 473.0440.

*N-((4,6-bis(2,2,2-Trifluoroethoxy)pyrimidin-2-yl)carbamoyl)-2-bromobenzamide* (**3**). White solid, mp 110–112 °C, yield = 77%. ^1^H-NMR (DMSO-*d*_6_) δ 11.46 (s, 1H, NH), 10.96 (s, 1H, NH), 7.73 (d, *J* = 7.9 Hz, 1H, Ph), 7.62 (dd, *J* = 7.4, 1.7 Hz, 1H, Ph), 7.57–7.42 (m, 2H, Ph), 6.39 (s, 1H, ArH), 5.07 (q, *J* = 8.9 Hz, 4H, OCH_2_CF_3_). ^13^C-NMR (DMSO-*d*_6_) δ 170.8, 170.0, 156.2, 149.0, 137.8, 133.7, 133.0, 129.8, 128.6, 125.6, 119.5, 86.8, 63.6, 63.3, 63.0, 62.7. HRMS (ESI): *m*/*z* calcd for C_16_H_11_BrF_6_N_4_O_4_ [M + Na]^+^: 538.9766, found 538.9769.

*N-((4,6-bis(2,2,2-Trifluoroethoxy)pyrimidin-2-yl)carbamoyl)-2-iodobenzamide* (**4**). White solid, mp 115–116 °C, yield = 88%. ^1^H-NMR (DMSO-*d*_6_) δ 11.42 (s, 1H, NH), 11.01 (s, 1H, NH), 7.95 (dd, *J* = 7.9, 1.0 Hz, 1H, Ph), 7.72–7.38 (m, 2H, Ph), 7.28 (td, *J* = 7.6, 1.9 Hz, 1H, Ph), 6.39 (s, 1H, ArH), 5.07 (q, *J* = 8.9 Hz, 4H, OCH_2_CF_3_). ^13^C-NMR (DMSO-*d*_6_) δ 171.7, 170.9, 156.3, 149.1, 141.7, 140.2, 132.9, 129.2, 129.1, 125.7, 125.4, 93.9, 86.8, 63.6, 63.4, 63.1, 62.8. HRMS (ESI): *m*/*z* calcd for C_16_H_11_F_6_IN_4_O_4_ [M + Na]^+^: 586.9627, found 586.9603.

*N-((4,6-bis(2,2,2-Trifluoroethoxy)pyrimidin-2-yl)carbamoyl)-4-chlorobenzamide* (**5**). White solid, mp 162–163 °C, yield = 94%. ^1^H-NMR (DMSO-*d*_6_) δ 11.42 (s, 1H, NH), 11.22 (s, 1H, NH), 8.02 (d, *J* = 8.5 Hz, 2H, Ph), 7.60 (d, *J* = 8.6 Hz, 2H, Ph), 6.36 (s, 1H, ArH), 5.07 (q, *J* = 8.9 Hz, 4H, OCH_2_CF_3_). ^13^C-NMR (DMSO-*d*_6_) δ 170.8, 168.0, 156.4, 149.4, 139.1, 132.1, 131.1, 129.6, 125.6, 86.4, 63.6, 63.3, 63.0, 62.8. HRMS (ESI): *m*/*z* calcd for C_16_H_11_ClF_6_N_4_O_4_ [M + H]^+^: 473.0451, found 473.0442.

*N-((4,6-bis(2,2,2-Trifluoroethoxy)pyrimidin-2-yl)carbamoyl)-2-(trifluoromethyl)benzamide* (**6**). White solid, mp 51–53 °C, yield = 68%. ^1^H-NMR (DMSO-*d*_6_) δ 11.55 (s, 1H, NH), 10.91 (s, 1H, NH), 7.88 (d, *J* = 7.7 Hz, 1H, Ph), 7.85–7.71 (m, 3H, Ph), 6.40 (s, 1H, ArH), 5.07 (q, *J* = 8.9 Hz, 4H, OCH_2_CF_3_). ^13^C-NMR (DMSO-*d*_6_) δ 170.8, 170.0, 156.3, 149.0, 134.8, 133.5, 131.9, 129.3, 127.4, 127.1, 126.8, 126.5, 126.3, 125.6, 123.4, 86.7, 63.6, 63.3, 63.0, 62.8. HRMS (ESI): *m*/*z* calcd for C_17_H_11_F_9_N_4_O_4_ [M + H]^+^: 507.0715, found 507.0711.

*N-((4,6-bis(2,2,2-Trifluoroethoxy)pyrimidin-2-yl)carbamoyl)-4-(trifluoromethyl)benzamide* (**7**). White solid, mp 167–169 °C, yield = 93%. ^1^H-NMR (DMSO-*d*_6_) δ 11.61 (s, 1H, NH), 11.15 (s, 1H, NH), 8.17 (d, *J* = 8.1 Hz, 2H, Ph), 7.90 (d, *J* = 8.1 Hz, 2H, Ph), 6.38 (s, 1H, ArH), 5.07 (q, *J* = 8.9 Hz, 4H, OCH_2_CF_3_). ^13^C-NMR (DMSO-*d*_6_) δ 170.9, 167.9, 156.4, 149.3, 137.4, 133.7, 133.5, 130.1, 126.5, 126.5, 125.8, 125.6, 86.5, 63.7, 63.4, 63.1, 62.8. HRMS (ESI): *m*/*z* calcd for C_17_H_11_F_9_N_4_O_4_ [M + H]^+^: 507.0715, found 507.0713.

*N-((4,6-bis(2,2,2-Trifluoroethoxy)pyrimidin-2-yl)carbamoyl)-5-chloro-2-methoxybenzamide* (**8**). White solid, mp 164–166 °C, yield = 95%. ^1^H-NMR (DMSO-*d*_6_) δ 11.04 (s, 1H, NH), 10.98 (s, 1H, NH), 7.65 (d, *J* = 2.7 Hz, 1H, Ph), 7.61 (dd, *J* = 8.9, 2.8 Hz, 1H, Ph), 7.23 (d, *J* = 8.9 Hz, 1H, Ph), 6.39 (s, 1H, ArH), 5.06 (q, *J* = 8.9 Hz, 4H, OCH_2_CF_3_), 3.89 (s, 3H, CH_3_). ^13^C-NMR (DMSO-*d*_6_) δ 170.9, 167.3, 156.6, 156.3, 148.9, 133.9, 130.1, 125.4, 125.1, 115.3, 86.7, 63.5, 63.3, 63.0, 62.7, 57.5. HRMS (ESI): *m*/*z* calcd for C_17_H_13_ClF_6_N_4_O_5_ [M + H]^+^: 503.0557, found 503.0535.

*N-((4,6-bis(2,2,2-Trifluoroethoxy)pyrimidin-2-yl)carbamoyl)-2,4-dichlorobenzamide* (**9**). White solid, mp 109–110 °C, yield = 92%. ^1^H-NMR (DMSO-*d*_6_) δ 11.49 (s, 1H, NH), 10.88 (s, 1H, NH), 7.79 (d, *J* = 1.9 Hz, 1H, Ph), 7.68 (d, *J* = 8.2 Hz, 1H, Ph), 7.58 (dd, *J* = 8.2, 2.0 Hz, 1H, Ph), 6.40 (s, 1H, ArH), 5.06 (q, *J* = 8.9 Hz, 4H, OCH_2_CF_3_). ^13^C-NMR (DMSO-*d*_6_) δ 170.8, 168.3, 156.3, 149.0, 136.9, 134.6, 132.0, 131.4, 130.3, 128.5, 125.6, 86.8, 63.6, 63.3, 63.0. HRMS (ESI): *m*/*z* calcd for C_16_H_10_Cl_2_F_6_N_4_O_4_ [M + Na]^+^: 528.9881, found 528.9866.

*N-((4,6-bis(2,2,2-Trifluoroethoxy)pyrimidin-2-yl)carbamoyl)-2,6-dichlorobenzamide* (**10**). White solid, mp 62–64 °C, yield = 81%. ^1^H-NMR (DMSO-*d*_6_) δ 11.68 (s, 1H, NH), 10.80 (s, 1H, NH), 7.82–7.39 (m, 3H, Ph), 6.41 (s, 1H, ArH), 5.08 (q, *J* = 8.9 Hz, 4H, OCH_2_CF_3_). ^13^C-NMR (DMSO-*d*_6_) δ 170.9, 156.2, 148.8, 131.4, 129.2, 127.8, 125.6, 123.4, 121.2, 100.5, 86.9, 63.7, 63.4, 63.1, 62.9. HRMS (ESI): *m*/*z* calcd for C_16_H_10_Cl_2_F_6_N_4_O_4_ [M + Na]^+^: 528.9881, found 528.9866.

*2-Chloro-N-((4,6-dimethylpyrimidin-2-yl)carbamoyl)benzamide* (**11**). White solid, mp 186–188 °C, yield = 87%. ^1^H-NMR (DMSO-*d*_6_) δ 12.51 (s, 1H, NH), 10.61 (s, 1H, NH), 7.64 (dd, *J* = 7.6, 1.6 Hz, 1H, Ph), 7.61–7.52 (m, 2H, Ph), 7.47 (td, *J* = 7.3, 1.5 Hz, 1H, Ph), 6.98 (s, 1H, ArH), 2.33 (s, 6H, Ar-CH_3_). ^13^C-NMR (DMSO-*d*_6_) δ 168.8, 167.5, 157.6, 149.8, 136.3, 132.9, 130.8, 130.6, 129.8, 128.3, 116.0, 24.2. HRMS (ESI): *m*/*z* calcd for C_14_H_13_ClN_4_O_2_ [M + Na]^+^: 327.0625, found 327.0629.

*2-Chloro-N-((4,6-dimethoxypyrimidin-2-yl)carbamoyl)benzamide* (**12**). White solid, mp 155–157 °C, yield = 89%. ^1^H-NMR (DMSO-*d*_6_) δ 11.87 (s, 1H, NH), 10.67 (s, 1H, NH), 7.62 (dd, *J* = 7.6, 1.6 Hz, 1H, Ph), 7.59–7.50 (m, 2H, Ph), 7.46 (td, *J* = 7.3, 1.5 Hz, 1H, Ph), 5.97 (s, 1H, ArH), 3.82 (s, 6H, Ar-OCH_3_). ^13^C-NMR (DMSO-*d*_6_) δ 172.4, 168.4, 156.9, 149.4, 136.3, 132.8, 130.6, 130.5, 129.7, 128.3, 85.1, 55.2. HRMS (ESI): *m*/*z* calcd for C_14_H_13_ClN_4_O_4_ [M + Na]^+^: 359.0523, found 359.0524.

*2-Chloro-N-((4,6-diethoxypyrimidin-2-yl)carbamoyl)benzamide* (**13**). White solid, mp 113–115 °C, yield = 91%. ^1^H-NMR (DMSO-*d*_6_) δ 11.92 (s, 1H, NH), 10.61 (s, 1H, NH), 7.65 (dd, *J* = 7.6, 1.5 Hz, 1H, Ph), 7.57 (qd, *J* = 8.1, 4.1 Hz, 2H, Ph), 7.52–7.44 (m, 1H, Ph), 5.87 (s, 1H, ArH), 4.20 (q, *J* = 7.0 Hz, 4H, OCH_2_CH_3_), 1.26 (t, *J* = 7.1 Hz, 6H, OCH_2_CH_3_). ^13^C-NMR (DMSO-*d*_6_) δ 171.9, 167.9, 156.9, 149.4, 136.3, 135.8, 132.8, 130.6, 129.7, 128.3, 85.3, 64.5, 63.6, 15.2, 15.0. HRMS (ESI): *m*/*z* calcd for C_16_H_17_ClN_4_O_4_ [M + Na]^+^: 387.0836, found 387.0851.

*2-Chloro-N-((4,6-dichloropyrimidin-2-yl)carbamoyl)benzamide* (**14**). White solid, mp 156–158 °C, yield = 88%. ^1^H-NMR (DMSO-*d*_6_) δ 11.52 (s, 1H, NH), 11.24 (s, 1H, NH), 7.69 (s, 1H, ArH), 7.65 (dd, *J* = 7.6, 1.5 Hz, 1H, Ph), 7.57 (qd, *J* = 8.1, 1.7 Hz, 2H, Ph), 7.49 (td, *J* = 7.2, 1.9 Hz, 1H, Ph). ^13^C-NMR (DMSO-*d*_6_) δ 169.0, 162.5, 157.4, 148.8, 133.1, 130.8, 130.8, 130.0, 128.3, 117.0. HRMS (ESI): *m*/*z* calcd for C_12_H_7_Cl_3_N_4_O_2_ [M + Na]^+^: 366.9532, found 366.9549.

*2-Chloro-N-((4-chloro-6-methylpyrimidin-2-yl)carbamoyl)benzamide* (**15**). White solid, mp 126–128 °C, yield = 93%. ^1^H-NMR (DMSO-*d*_6_) δ 11.89 (s, 1H, NH), 10.98 (s, 1H, NH), 7.65 (dd, *J* = 7.6, 1.6 Hz, 1H, Ph), 7.63–7.51 (m, 2H, Ph), 7.47 (td, *J* = 7.3, 1.5 Hz, 1H, Ph), 7.32 (s, 1H, ArH), 2.40 (s, 3H, CH_3_). ^13^C-NMR (DMSO-*d*_6_) δ 171.4, 169.1, 168.3, 161.4, 157.5, 149.2, 138.1, 131.5, 130.5, 129.6, 127.9, 116.4, 24.2. HRMS (ESI): *m*/*z* calcd for C_13_H_10_Cl_2_N_4_O_2_ [M + Na]^+^: 347.0079, found 347.0064.

*2-Chloro-N-((4-chloro-6-methoxypyrimidin-2-yl)carbamoyl)benzamide* (**16**). White solid, mp 166–167 °C, yield = 87%. ^1^H-NMR (DMSO-*d*_6_) δ 11.64 (s, 1H, NH), 10.98 (s, 1H, NH), 7.65 (d, *J* = 7.5 Hz, 1H, Ph), 7.56 (d, *J* = 8.5 Hz, 2H, Ph), 7.48 (t, *J* = 7.3 Hz, 1H, Ph), 6.82 (s, 1H, ArH), 3.91 (s, 3H, OCH_3_). ^13^C-NMR (DMSO-*d*_6_) δ 171.9, 168.6, 160.8, 157.2, 149.0, 135.7, 132.9, 130.6, 129.9, 128.2, 102.6, 55.7. HRMS (ESI): *m*/*z* calcd for C_13_H_10_Cl_2_N_4_O_3_ [M + Na]^+^: 363.0028, found 363.0013.

*2-Chloro-N-((4-chloro-6-(2,2,2-trifluoroethoxy)pyrimidin-2-yl)carbamoyl)benzamide* (**17**). White solid, mp 144–146 °C, yield = 88%. ^1^H-NMR (DMSO-*d*_6_) δ 11.51 (s, 1H, NH), 11.07 (s, 1H, NH), 7.65 (dd, *J* = 7.5, 1.5 Hz, 1H, Ph), 7.62–7.51 (m, 2H, Ph), 7.48 (td, *J* = 7.3, 1.7 Hz, 1H, Ph), 7.09 (s, 1H, ArH), 5.10 (q, *J* = 8.8 Hz, 2H, OCH_2_CF_3_). ^13^C-NMR (DMSO-*d*_6_) δ 170.0, 169.1, 161.8, 156.9, 148.9, 135.6, 133.1, 130.8, 130.0, 128.2, 125.5, 123.3, 103.0, 63.8, 63.5, 63.2, 62.9. HRMS (ESI): *m*/*z* calcd for C_14_H_9_Cl_2_F_3_N_4_O_3_ [M + Na]^+^: 430.9901, found 430.9887.

*2-Chloro-N-((4-chloro-6-morpholinopyrimidin-2-yl)carbamoyl)benzamide* (**18**). White solid, mp 147–149 °C, yield = 85%. ^1^H-NMR (DMSO-*d*_6_) δ 11.78 (s, 1H, NH), 10.59 (s, 1H, NH), 7.62 (dd, *J* = 7.6, 1.6 Hz, 1H, Ph), 7.59–7.50 (m, 2H, Ph), 7.46 (td, *J* = 7.3, 1.5 Hz, 1H, Ph), 6.71 (s, 1H, ArH), 3.58 (dd, *J* = 23.7, 4.9 Hz, 8H, morpholine). ^13^C-NMR (DMSO-*d*_6_) δ 168.2, 163.6, 157.0, 149.3, 136.1, 132.9, 130.7, 130.6, 129.8, 128.3, 97.9, 66.6, 60.7. HRMS (ESI): *m*/*z* calcd for C_16_H_15_Cl_2_N_5_O_3_ [M + Na]^+^: 418.0450, found 418.0441.

*2-Chloro-N-((4-chloro-6-(diethylamino)pyrimidin-2-yl)carbamoyl)benzamide* (**19**). white solid, mp 151–153 °C, yield = 83%. ^1^H-NMR (DMSO-*d*_6_) δ 11.97 (s, 1H, NH), 10.46 (s, 1H, NH), 7.60 (dd, *J* = 7.6, 1.6 Hz, 1H, Ph), 7.58–7.49 (m, 2H, Ph), 7.45 (td, *J* = 7.3, 1.6 Hz, 1H, Ph), 6.49 (s, 1H, ArH), 3.44 (s, 4H, CH_2_CH_3_), 1.19–0.91 (m, 6H, CH_2_CH_3_). ^13^C-NMR (DMSO-*d*_6_) δ 168.1, 162.1, 159.4, 157.2, 149.6, 136.5, 132.7, 130.5, 130.5, 129.7, 128.3, 97.1, 43.3, 13.4. HRMS (ESI): *m*/*z* calcd for C_16_H_17_Cl_2_N_5_O_2_ [M + Na]^+^: 404.0657, found 404.0642.

*2-Chloro-N-((4-methyl-6-(2,2,2-trifluoroethoxy)pyrimidin-2-yl)carbamoyl)benzamide* (**20**). White solid, mp 56–58 °C, yield = 82%. ^1^H-NMR (DMSO-*d*_6_) δ 12.13 (s, 1H, NH), 10.79 (s, 1H, NH), 7.72–7.63 (m, 1H, Ph), 7.63–7.52 (m, 2H, Ph), 7.49 (td, *J* = 7.3, 1.6 Hz, 1H, Ph), 6.71 (s, 1H, ArH), 5.07 (q, *J* = 8.9 Hz, 2H, OCH_2_CF_3_), 2.34 (s, 3H, CH_3_). ^13^C-NMR (DMSO-*d*_6_) δ 171.4, 170.3, 169.3, 168.1, 161.4, 157.0, 149.4, 136.0, 132.9, 130.7, 129.9, 128.2, 102.1, 62.8, 62.5, 62.2, 61.9, 24.1. HRMS (ESI): *m*/*z* calcd for C_15_H_12_ClF_3_N_4_O_3_ [M + Na]^+^: 411.0448, found 411.0442.

*2-Chloro-N-((4-(pyrrolidin-1-yl)-6-(2,2,2-trifluoroethoxy)pyrimidin-2-yl)carbamoyl)benzamide* (**21**). White solid, mp 121–122 °C, yield = 81%. ^1^H-NMR (DMSO-*d*_6_) δ 12.55 (s, 1H, NH), 10.28 (s, 1H, NH), 7.61 (d, *J* = 7.4 Hz, 1H, Ph), 7.58–7.50 (m, 2H, Ph), 7.46 (td, *J* = 7.3, 1.7 Hz, 1H, Ph), 5.62 (s, 1H, ArH), 4.97 (q, *J* = 9.0 Hz, 2H, OCH_2_CF_3_), 3.25 (s, 2H, tetrahydropyrrole), 3.02 (s, 2H, tetrahydropyrrole), 1.97–1.82 (m, 2H, tetrahydropyrrole), 1.77–1.60 (m, 2H, tetrahydropyrrole). ^13^C-NMR (DMSO-*d*_6_) δ 168.7, 167.3, 161.8, 156.7, 149.8, 136.9, 132.5, 130.5, 130.3, 129.4, 128.3, 125.9, 81.1, 62.2, 61.9, 61.6, 47.3, 25.7. HRMS (ESI): *m*/*z* calcd for C_18_H_17_ClF_3_N_5_O_3_ [M + H]^+^: 444.1050, found 444.1047.

*2-Chloro-N-((4-(hexylamino)-6-(2,2,2-trifluoroethoxy)pyrimidin-2-yl)carbamoyl)benzamide* (**22**). White solid, mp 126–128 °C, yield = 83%. ^1^H-NMR (DMSO-*d*_6_) δ 12.40 (s, 1H, NH), 10.28 (s, 1H, NH), 7.85–7.36 (m, 5H, Ph), 5.62 (s, 1H, ArH), 4.93 (q, *J* = 9.0 Hz, 2H, OCH_2_CF_3_), 3.05 (s, 2H, N–CH_2_(CH_2_)_4_CH_3_), 1.46–1.07 (m, 8H, N–CH_2_(CH_2_)_4_CH_3_), 0.86 (t, *J* = 6.8 Hz, 3H, N(CH_2_)_5_CH_3_). ^13^C-NMR (DMSO-*d*_6_) δ 168.0, 165.1, 157.1, 151.8, 149.8, 136.7, 132.6, 130.5, 129.6, 128.3, 125.9, 123.6, 82.2, 62.1, 61.8, 41.3, 32.0, 29.6, 27.0, 23.1, 14.9. HRMS (ESI): *m*/*z* calcd for C_20_H_23_ClF_3_N_5_O_3_ [M + Na]^+^: 496.1339, found 496.1357.

*2,4-Dichloro-N-((4,6-dimethylpyrimidin-2-yl)carbamoyl)benzamide* (**23**). White solid, mp 185–186 °C, yield = 81%. ^1^H-NMR (DMSO-*d*_6_) δ 12.50 (s, 1H, NH), 10.60 (s, 1H, NH), 7.77 (d, *J* = 2.0 Hz, 1H, Ph), 7.67 (d, *J* = 8.2 Hz, 1H, Ph), 7.57 (dd, *J* = 8.2, 2.0 Hz, 1H, Ph), 6.99 (d, *J* = 2.7 Hz, 1H, ArH), 2.35 (s, 6H, CH_3_). ^13^C-NMR (DMSO-*d*_6_) δ 168.8, 167.0, 157.6, 149.8, 136.5, 135.4, 131.8, 131.1, 130.3, 128.5, 116.1, 24.3. HRMS (ESI): *m*/*z* calcd for C_14_H_12_Cl_2_N_4_O_2_ [M + Na]^+^: 361.0235, found 361.0208.

*2,4-Dichloro-N-((4-methyl-6-(2,2,2-trifluoroethoxy)pyrimidin-2-yl)carbamoyl)benzamide* (**24**). White solid, mp 155–157 °C, yield = 81%. ^1^H-NMR (DMSO-*d*_6_) δ 12.14 (s, 1H, NH), 10.77 (s, 1H, NH), 7.76 (d, *J* = 2.0 Hz, 1H, Ph), 7.70 (d, *J* = 8.3 Hz, 1H, Ph), 7.64–7.54 (m, 1H, Ph), 6.71 (s, 1H, ArH), 5.07 (q, *J* = 8.9 Hz, 2H, OCH_2_CF_3_), 2.37 (s, 3H, CH_3_). ^13^C-NMR (DMSO-*d*_6_) δ 170.4, 169.3, 167.5, 157.1, 149.5, 136.7, 135.0, 132.0, 131.2, 130.3, 128.5, 125.7, 102.2, 62.9, 62.6, 62.3, 62.0, 24.2. HRMS (ESI): *m*/*z* calcd for C_15_H_11_Cl_2_F_3_N_4_O_3_ [M + Na]^+^: 445.0058, found 445.0064.

*2,6-Dichloro-N-((4-chloro-6-(2,2,2-trifluoroethoxy)pyrimidin-2-yl)carbamoyl)benzamide* (**25**). White solid, mp 125–126 °C, yield = 87%. ^1^H-NMR (-DMSO-*d*_6_) δ 11.64 (s, 1H, NH), 10.90 (s, 1H, NH), 7.59 (d, *J* = 8.9 Hz, 2H, Ph), 7.56–7.48 (m, 1H, Ph), 7.12 (s, 1H, ArH), 5.11 (q, *J* = 8.9 Hz, 2H, OCH_2_CF_3_). ^13^C-NMR (DMSO-*d*_6_) δ 170.0, 165.0, 161.8, 156.8, 148.7, 137.7, 132.1, 131.9, 129.0, 125.4, 103.2, 63.6, 63.3, 63.0. HRMS (ESI): *m*/*z* calcd for C_14_H_8_Cl_3_F_3_N_4_O_3_ [M + Na]^+^: 464.9512, found 464.9502.

*2,6-Dichloro-N-((4-chloro-6-(diethylamino)pyrimidin-2-yl)carbamoyl)benzamide* (**26**). White solid, mp 169–171 °C, yield = 81%. ^1^H-NMR (DMSO-*d*_6_) δ 12.42 (s, 1H, NH), 10.44 (s, 1H, NH), 7.61–7.47 (m, 3H, Ph), 6.54 (s, 1H, ArH), 3.46 (s, 4H, CH_2_CH_3_), 1.12 (s, 6H, CH_2_CH_3_). ^13^C-NMR (DMSO-*d*_6_) δ 165.0, 164.0, 160.9, 158.5, 136.7, 131.1, 130.9, 128.1, 96.2, 42.6, 25.8, 12.4. HRMS (ESI): *m*/*z* calcd for C_16_H_16_Cl_3_N_5_O_2_ [M + H]^+^: 416.0448, found 416.0444.

*2,6-Dichloro-N-((4-(diethylamino)-6-(2,2,2-trifluoroethoxy)pyrimidin-2-yl)carbamoyl)benzamide* (**27**). White solid, mp 140–142 °C, yield = 87%. ^1^H-NMR (DMSO-*d*_6_) δ 12.84 (s, 1H, NH), 10.22 (s, 1H, NH), 8.60–6.40 (m, 3H, Ph), 5.84 (s, 1H, ArH), 4.96 (q, *J* = 9.0 Hz, 2H, OCH_2_CF_3_), 3.46 (s, 4H, CH_2_CH_3_), 1.12 (s, 6H, CH_2_CH_3_). ^13^C-NMR (DMSO-*d*_6_) δ 169.2, 168.2, 163.3, 156.7, 149.8, 138.1, 131.5, 130.6, 129.7, 127.9, 80.4, 62.6, 62.3, 62.0, 61.7, 43.2, 13.4. HRMS (ESI): *m*/*z* calcd for C_18_H_18_Cl_2_F_3_N_5_O_3_ [M + Na]^+^: 502.0636, found 502.0635.

*N-((4-(Benzylamino)-6-(2,2,2-trifluoroethoxy)pyrimidin-2-yl)carbamoyl)-2,6-dichlorobenzamide* (**28**). White solid, mp 76–78 °C, yield = 80%. ^1^H-NMR (DMSO-*d*_6_) δ 12.46 (d, *J* = 369.0 Hz, 1H, NH), 10.27 (s, 1H, NH), 8.25 (s, 1H, Ar-NH), 7.82–7.40 (m, 3H, Ph), 7.42–7.14 (m, 5H, Ph), 5.70 (s, 1H, ArH), 4.93 (q, *J* = 9.0 Hz, 2H, OCH_2_CF_3_), 4.39 (s, 2H, N–CH_2_Ph). ^13^C-NMR (DMSO-*d*_6_) δ 164.0, 155.9, 151.0, 148.6, 138.7, 136.7, 131.1, 130.8, 129.9, 128.3, 128.0, 127.9, 127.0, 126.1, 124.8, 122.6, 120.4, 81.6, 61.6, 61.3, 61.0, 60.7, 44.2, 30.6, 25.7. HRMS (ESI): *m*/*z* calcd for C_21_H_16_Cl_2_F_3_N_5_O_3_ [M + Na]^+^: 536.0480, found 536.0485.

*N-((4-(Benzyl(ethyl)amino)-6-(2,2,2-trifluoroethoxy)pyrimidin-2-yl)carbamoyl)-2,6-dichlorobenzamide* (**29**). White solid, mp 61–62 °C, yield = 75%. ^1^H-NMR (DMSO-*d*_6_) δ 12.72 (s, 1H, NH), 10.28 (s, 1H, NH), 7.71–7.08 (m, 8H, Ph), 5.85 (s, 1H, ArH), 4.95 (d, *J* = 9.0 Hz, 2H, OCH_2_CF_3_), 4.74 (s, 2H, N–CH_2_Ph), 3.41 (d, *J* = 52.6 Hz, 2H, CH_2_CH_3_), 1.08 (s, 3H, CH_2_CH_3_). ^13^C-NMR (DMSO-*d*_6_) δ 169.3, 167.5, 166.0, 165.2, 164.7, 156.6, 156.3, 133.7, 131.1, 129.8, 129.5, 129.0, 128.9, 128.1, 125.8, 123.6, 81.1, 62.5, 62.2, 61.9, 60.7, 30.9, 15.0. HRMS (ESI): *m*/*z* calcd for C_23_H_20_Cl_2_F_3_N_5_O_3_ [M + H]^+^: 542.0974, found 542.0971.

*N-((5-(Sec-butyl)-4-chloro-6-(2,2,2-trifluoroethoxy)pyrimidin-2-yl)carbamoyl)-2,6-dichlorobenzamide* (**30**). White solid, mp 37–38 °C, yield = 73%. ^1^H-NMR (DMSO-*d*_6_) δ 11.64 (s, 1H, NH), 10.81 (s, 1H, NH), 7.76–7.39 (m, 3H, Ph), 5.16 (q, *J* = 8.8 Hz, 2H, OCH_2_CF_3_), 3.21 (dt, *J* = 9.2, 6.8 Hz, 1H, CH), 1.90–1.74 (m, 1H, CHCH_2_CH_3_), 1.68 (dt, *J* = 13.8, 6.7 Hz, 1H, CHCH_2_CH_3_), 1.29 (d, *J* = 7.1 Hz, 3H, CHCH_3_), 0.83 (t, *J* = 7.4 Hz, 3H, CHCH_2_CH_3_). ^13^C-NMR (DMSO-*d*_6_) δ 167.7, 166.7, 160.4, 154.0, 148.7, 131.2, 129.1, 125.6, 123.4, 118.3, 63.9, 63.6, 63.3, 63.0, 31.6, 27.4, 18.6, 13.2. HRMS (ESI): *m*/*z* calcd for C_18_H_16_Cl_3_F_3_N_4_O_3_ [M + Na]^+^: 521.0138, found 521.0157.

### 3.10. Synthesis of N-((4,6-bis(2,2,2-Trifluoroethoxy)pyrimidin-2-yl)carbamothioyl)-2-chlorobenzamide *(**31**)*

Compound **31** was prepared by the method given in [22]. 2-Chlorobenzoic acid (5 mmol) and thionyl chloride (10 mL) were added to a 100 mL three-necked flask under N_2_. The reaction mixture was refluxed for 3 h. The excess thionyl chloride was evaporated under reduced pressure to give a colorless transparent liquid. Anhydrous acetonitrile (5 mL) was added to the residue, then KSCN (5 mmol) was added to the system and reacted at 80 °C for 1 h. The reaction mixture was cooled to the room temperature, filtered and neutralized with triethylamine. Then intermediate **Ia** (5 mmol) and TBAB (10 mmol) were added into reaction mixture. The mixture was heated to reflux for 4 h. When the reaction was completed, the reaction mixture was cooled to room temperature, diluted with 20 mL of EtOAc, washed twice with brine (20 mL), dried with anhydrous Na_2_SO_4_ and evaporated in vacuo. Finally, the residue was purified by silica gel column chromatography to afford compound **31** as a yellow solid, mp 93–95 °C, yield = 53%. ^1^H-NMR (DMSO-*d*_6_) δ 12.84 (s, 1H, NH), 12.45 (s, 1H, NH), 7.71–7.62 (m, 1H, Ph), 7.56 (dd, *J* = 6.5, 1.7 Hz, 2H, Ph), 7.51–7.42 (m, 1H, Ph), 6.51 (s, 1H, ArH), 5.11 (q, *J* = 8.9 Hz, 4H, OCH_2_CF_3_). ^13^C-NMR (DMSO-*d*_6_) δ 178.3, 170.8, 168.3, 156.2, 135.2, 133.3, 131.1, 130.7, 130.3, 128.2, 125.6, 123.4, 88.2, 63.9, 63.6, 63.3, 63.0. HRMS (ESI): *m*/*z* calcd for C_16_H_11_ClF_6_N_4_O_3_S [M + Na]^+^: 511.0042, found 511.0015.

### 3.11. Synthesis of 2,6-Dichloro-N-((4-(diethylamino)-6-(2,2,2-trifluoroethoxy)pyrimidin-2-yl)carbamothioyl)-benzamide *(**32a**)*

Compound **32a** was synthesized by a method similar to that used for compound **31**. Yellow solid, mp 42–43 °C, yield = 59%. ^1^H-NMR (DMSO-*d*_6_) δ 12.84 (s, 1H, NH), 10.22 (s, 1H, NH), 7.93–7.14 (m, 3H, Ph), 5.84 (s, 1H, ArH), 4.96 (q, *J* = 9.0 Hz, 2H, OCH_2_CF_3_), 3.46 (s, 4H, CH_2_CH_3_), 1.12 (s, 6H, CH_2_CH_3_). ^13^C-NMR (DMSO-*d*_6_) δ 169.2, 168.2, 163.2, 156.7, 149.8, 132.5, 131.5, 130.6, 129.7, 127.9, 80.4, 62.6, 62.3, 62.0, 61.7, 43.2, 13.4. HRMS (ESI): *m*/*z* calcd for C_18_H_18_Cl_2_F_3_N_5_O_2_S [M + Na]^+^: 518.0408, found 518.0407.

### 3.12. Synthesis of 2,6-dichloro-N-(N-(4-(Diethylamino)-6-(2,2,2-trifluoroethoxy)pyrimidin-2-yl)-carbamimidoyl)benzamide *(**32**)*

Compound **32** was synthesized by the method described in [23]. A solution of compound **32a** (0.37 g, 0.75 mmol) and EDCI (0.28 g, 1.49 mmol) was stirred at 0 °C for 30 min and hexamethyldisilazide (HMDS, 1.2 g, 7.4 mmol) was added. After the mixture was stirred for 3 h under 0 °C, the mixture was raised to room temperature and stood for 24 h. The organic layer was diluted with EtOAc (20 mL), washed twice with a saturated NaHCO_3_ solution (20 mL) and brine (20 mL), dried with anhydrous Na_2_SO_4_ and evaporated in vacuo. Finally, the residue was purified by silica gel column chromatography to afford compound **32** as a white solid, mp 166–168 °C, yield = 81%. ^1^H-NMR (DMSO-*d*_6_) δ 11.25 (s, 1H, NH), 9.56 (s, 1H, NH), 9.48 (s, 1H, NH), 7.46 (d, *J* = 8.1 Hz, 2H, Ph), 7.35 (dd, *J* = 8.8, 7.5 Hz, 1H, Ph), 5.85 (s, 1H, ArH), 4.98 (q, *J* = 9.0 Hz, 2H, OCH_2_CF_3_), 3.69–3.40 (m, 4H, CH_2_CH_3_), 1.13 (t, *J* = 7.0 Hz, 6H, CH_2_CH_3_). ^13^C-NMR (DMSO-*d*_6_) δ 176.3, 169.0, 163.2, 160.1, 157.5, 140.6, 131.0, 130.6, 129.0, 128.9, 126.0, 123.7, 80.8, 62.7, 62.4, 62.2, 43.5, 13.6. HRMS (ESI): *m*/*z* calcd for C_18_H_19_Cl_2_F_3_N_6_O_2_ [M + Na]^+^: 501.0796, found 501.0791.

### 3.13. Synthesis of the Target Compounds ***33–38***

Compounds **33**–**38** were prepared by a method similar to that used for compounds **1**–**30**.

*5-Chloro-N-((4-chloro-6-(diethylamino)pyrimidin-2-yl)carbamoyl)-1-methyl-1H-pyrazole-4-carboxamide* (**33**). White solid, mp 96–97 °C, yield = 88%. ^1^H-NMR (DMSO-*d*_6_) δ 11.27 (s, 1H, NH), 10.73 (s, 1H, NH), 8.30 (s, 1H, ArH), 6.50 (s, 1H, ArH), 3.86 (s, 3H, N–CH_3_), 3.43 (s, 4H, CH_2_CH_3_), 1.11 (t, *J* = 7.0 Hz, 6H, CH_2_CH_3_). ^13^C-NMR (DMSO-*d*_6_) δ 162.7, 162.0, 159.0, 157.1, 149.7, 140.2, 131.8, 113.0, 97.3, 42.9, 37.6, 13.6. HRMS (ESI): *m*/*z* calcd for C_14_H_17_Cl_2_N_7_O_2_ [M + H]^+^: 386.0899, found 386.0929.

*N-((5-(sec-Butyl)-4-chloro-6-(diethylamino)pyrimidin-2-yl)carbamoyl)-5-chloro-1-methyl-1H-pyrazole-4-carboxamide* (**34**). White solid, mp 159–161 °C, yield = 75%. ^1^H-NMR (DMSO-*d*_6_) δ 11.10 (s, 1H, NH), 10.87 (s, 1H, NH), 8.34 (s, 1H, ArH), 3.86 (s, 3H, N-CH_3_), 3.23 (dq, *J* = 14.0, 7.0 Hz, 2H, CHCH_2_CH_3_), 2.79 (q, *J* = 7.3 Hz, 1H, CH), 1.78 (dtd, *J* = 15.2, 13.6, 7.3 Hz, 2H, CH_2_CH_3_), 1.39 (d, *J* = 7.2 Hz, 3H, CHCH_3_), 1.22 (t, *J* = 2.8 Hz, 2H, CH_2_CH_3_), 1.15 (t, *J* = 7.0 Hz, 6H, CH_2_CH_3_), 0.73 (t, *J* = 7.4 Hz, 3H, CHCH_2_CH_3_). ^13^C-NMR (DMSO-*d*_6_) δ 169.4, 162.3, 158.9, 153.5, 149.7, 140.2, 131.9, 119.1, 112.7, 45.7, 37.5, 35.2, 27.9, 18.4, 13.8, 13.4. HRMS (ESI): *m*/*z* calcd for C_18_H_25_Cl_2_N_7_O_2_ [M + Na]^+^: 464.1344, found 464.1363.

*5-Chloro-N-((4-chloro-6-(2,2,2-trifluoroethoxy)pyrimidin-2-yl)carbamoyl)-1-methyl-1H-pyrazole-4-carboxamide* (**35**). White solid, mp 181–183 °C, yield = 85%. ^1^H-NMR (DMSO-*d*_6_) δ 11.40 (s, 1H, NH), 11.10 (s, 1H, NH), 8.37 (s, 1H, ArH), 7.07 (s, 1H, ArH), 5.11 (q, *J* = 8.9 Hz, 2H, OCH_2_CF_3_), 3.86 (s, 3H, N–CH_3_). ^13^C-NMR (DMSO-*d*_6_) δ 169.9, 162.8, 161.8, 156.8, 149.4, 140.3, 132.2, 112.3, 102.9, 63.5, 63.2, 62.9, 37.5. HRMS (ESI): *m*/*z* calcd for C_12_H_9_Cl_2_F_3_N_6_O_3_ [M + H]^+^: 434.9963, found 434.9975.

*N-((5-(sec-Butyl)-4,6-dichloropyrimidin-2-yl)carbamoyl)-2-chloronicotinamide* (**36**). White solid, mp 139–140 °C, yield = 86%. ^1^H-NMR (DMSO-*d*_6_) δ 11.49 (s, 1H, NH), 10.99 (s, 1H, NH), 8.55 (dd, *J* = 4.9, 1.9 Hz, 1H, ArH), 8.09 (dd, *J* = 7.6, 1.9 Hz, 1H, ArH), 7.58 (dd, *J* = 7.6, 4.8 Hz, 1H, ArH), 3.40 (dt, *J* = 9.0, 7.1 Hz, 1H, CH), 2.01–1.85 (m, 1H, CHCH_2_CH_3_), 1.73 (dt, *J* = 13.9, 7.1 Hz, 1H, CHCH_2_CH_3_), 1.33 (d, *J* = 7.2 Hz, 3H, CHCH_3_), 0.81 (t, *J* = 7.4 Hz, 3H, CHCH_2_CH_3_). ^13^C-NMR (DMSO-*d*_6_) δ 167.9, 154.5, 152.1, 148.9, 146.8, 139.1, 132.4, 129.8, 124.0, 37.1, 27.1, 18.2, 13.3. HRMS (ESI): *m*/*z* calcd for C_15_H_14_Cl_3_N_5_O_2_ [M + Na]^+^: 424.0111, found 424.0112.

*2-Chloro-N-((4-(diethylamino)-6-(2,2,2-trifluoroethoxy)pyrimidin-2-yl)carbamoyl)nicotinamide* (**37**). White solid, mp 124–126 °C, yield = 85%. ^1^H-NMR (DMSO-*d*_6_) δ 12.33 (s, 1H, NH), 10.30 (s, 1H, NH), 8.53 (dd, *J* = 4.9, 1.9 Hz, 1H, ArH), 8.06 (dd, *J* = 7.6, 1.9 Hz, 1H, ArH), 7.55 (dd, *J* = 7.6, 4.9 Hz, 1H, ArH), 5.84 (s, 1H, ArH), 4.97 (q, *J* = 9.0 Hz, 2H, OCH_2_CF_3_), 3.47 (s, 4H, CH_2_CH_3_), 1.11 (t, *J* = 7.0 Hz, 6H, CH_2_CH_3_). ^13^C-NMR (DMSO-*d*_6_) δ 169.2, 167.3, 163.1, 156.7, 151.5, 146.5, 138.6, 133.5, 125.9, 124.0, 80.5, 62.6, 62.3, 62.1, 61.8, 43.4, 13.5. HRMS (ESI): *m*/*z* calcd for C_17_H_18_ClF_3_N_6_O_3_ [M + Na]^+^: 469.0979, found 469.0966.

*1-(4-Chloro-6-(diethylamino)pyrimidin-2-yl)-3-(2,6-dichlorophenyl)urea* (**38**). White solid, mp 98–99 °C, yield = 78%. ^1^H-NMR (DMSO-*d*_6_) δ 10.90 (s, 1H, NH), 10.11 (s, 1H, NH), 7.58 (d, *J* = 8.1 Hz, 2H, Ph), 7.37 (t, *J* = 8.1 Hz, 1H, Ph), 6.47 (s, 1H, ArH), 3.33 (s, 4H, CH_2_CH_3_), 2.50 (s, 6H, CH_2_CH_3_). ^13^C-NMR (DMSO-*d*_6_) δ 161.3, 158.9, 157.5, 151.8, 133.9, 132.8, 129.5, 129.0, 95.6, 43.1, 12.8. HRMS (ESI): *m*/*z* calcd for C_15_H_16_Cl_3_N_5_O [M + H]^+^: 388.0499, found 388.0488.

### 3.14. Insecticidal Biological Assay

All bioassays were performed on representative test organisms reared in the laboratory. The bioassay was repeated at 25 ± 1 °C according to statistical requirements. Assessments were made on a dead/alive basis and mortality rates were corrected using Abbott’s formula [24]. Evaluations are based on a percentage scale of 0–100 in which 0 = no activity and 100 = total kill.

#### 3.14.1. Toxicity against Mosquito (*Culex pipiens pallens*)

The toxicities of compounds **1**–**25**, **31** and **37** against mosquito were evaluated according to the reported procedure [25,26]. One milliliter of different concentrated dilutions of each compound was added to 99 mL of water to obtain different concentrations of tested solution. Then 20 fourth-instar mosquito larvae were put into the solution. Percentage mortalities were evaluated 8 day after treatment. For comparative purposes, fipronil was tested under the same conditions, and each test was performed in triplicate.

#### 3.14.2. Stomach Toxicity against Oriental Armyworm (*Mythimna separata*)

The stomach toxicities of compounds **1**–**25**, **31**, **37** and the contrast fipronil against oriental armyworm were evaluated by foliar application using the reported procedure [27]. For the foliar armyworm tests, individual corn leaves were placed on moistened pieces of filter paper in Petri dishes. The leaves were then sprayed with the test solution and allowed to dry. The dishes were infested with 10 fourth-instar Oriental armyworm larvae. Percentage mortalities were evaluated 3 days after treatment. Each treatment was performed three times

### 3.15. In Vitro Antifungal Bioassay

The antifungal activities were screened and evaluated by the poison plate technique [28]. All final compounds were dissolved in DMF (0.1 mL) before mixing with potato dextrose agar (PDA; 9.9 mL). The compounds were tested at a concentration of 50 μg mL^−1^. All fungi were cultivated in PDA at 27 ± 1 °C for 4 days to make new mycelium for the identification of antifungal activity. Then, mycelia dishes of approximately 5 mm diameter were cut from the culture medium. A mycelium was obtained using a germ-free inoculation needle and inoculated in the middle of the PDA plate aseptically. The inoculated plates were incubated at 27 ± 1 °C for 5 days. DMF in sterile distilled water served as the negative control, whereas hymexazol served as the positive control. Each treatment condition consisted of three replicates. Radial growth of the fungal colonies was measured, and the data were statistically analyzed. Inhibitory effects of the test compounds in vitro on these fungi were calculated by the formula I (%) = [(*C* − *T*)/(*C* − 0.5)] × 100, where *C* represents the diameter of fungal growth on untreated PDA, *T* represents the diameter of fungi on treated PDA, and *I* represents the inhibition rate.

### 3.16. In Vivo Antifungal Bioassay against Sclerotinia sclerotiorum

The requisite amounts of compounds **19** and **25** was disolved in sterile Tween 80 (0.1%, *v*/*v*) solution to give different concentration test solution (500, 1500 and 3000 μg mL^−1^). For protective activity assay [29], fresh leaves were sprayed with these solution (10 mL for each leave) until liquid flowed on surface at 24 h before inoculation. A colonized mycelial plug (5 mm in diameter) from a 5-day-old PDA culture of carbendazim-resistant *Sclerotinia sclerotiorum* was placed on the surface of fresh leaves. Inoculated leaves were placed at 25 °C with 80% relative humidity for disease development. After 5 days, the average lesion diameter was determined by measuring each lesion in two perpendicular directions. The lengths of the long and short axes were averaged and disease control efficacy was calculated as follows: disease control efficacy (%) = (lesion diameter in the water control-lesion diameter in the treatment)/lesion diameter in the water control × 100 [30].

### 3.17. Zebrafish Embryo Toxicity Assay

Zebrafish wild-type AB (*Danio rerio AB*) was introduced from the National Zebrafish Center (Wuhan, China). The zebrafish for experiment was cultured by the five-layer zebrafish culture system of Beijing Aisheng Technology Development Co., Ltd. (Beijing, China) to the fifth generation. Animals were housed in feeding system (pH 6.5–7.5, oxygen content >85%, water temperture 28 °C, conductivity 500 ± 50 μs cm^−1^, Light-dark ratio 14 h/10 h). Natural mortality during feeding was less than 1%. Fish were fed twice a day with *Artemia salina*, the total weight of feeding was 5 ± 1% of zebrafish body weight. For this experiment, a sexually mature 8-month zebrafish were used. Males and females were maintained separately until the night before the spawning at a ratio of 1:2 and the light-dark ratio was controlled at 14 h/10 h. Embryos were obtained by naturally mate [31].

Toxicological analyses were based on approved standard OECD TG 236: Fish Embryo Toxicity (FET) Test [32]. 20 embryos were distributed in each of different concentrations of the compounds to be analyzed, a positive control (3,4-dichloroaniline), a negative control (water) and a solvent control (10% Hank’s solution). All of the treatments were cared at 26 °C ± 1 °C, light/dark cycle of 14/10 h. The entire experiment was carried out for 96 h. All of the data were analyzed by SPSS 19.0 (IBM Corporation, New York, NY, USA), LC_50_ was corrected taking into account control mortality with Abbott’s formula [15].

## 4. Conclusions

In summary, a series of novel benzoylpyrimidinylurea derivatives were designed and synthesized. Although we did not find any compounds which possessed both insecticidal and antifungal as efficient as we expected, compounds **7** and **25** still exhibited excellent larvicidal activity against mosquito (*Culex pipiens pallens*) and broad-spectrum antifungal activity against fourteen phytopathogenic fungi, respectively. High efficiency, low toxicity and environmentally friendly pesticides are consistent with the requirements of sustainable agricultural development. Therefore, compounds **7** and **25** with low toxicity to zebrafish will be potential lead compounds to develop green mosquitocides and broad-spectrum fungicides.

## Figures and Tables

**Table 1:** Larvicidal Activities against Oriental Armyworm and Mosquito of Compounds **1**–**25**, **31**, **37**.

Compd.	Larvicidal Activity (%)										
	Oriental Armyworm (µg mL^−1^)				Mosquito (µg mL^−1^)						
	600	200	100		10	5	2	1	0.5	0.25	0.1
**1**	45	- ^a^	-		100	100	60	-	-	-	-
**2**	5	-	-		100	100	100	100	100	20	-
**3**	100	100	60		100	100	60	-	-	-	-
**4**	70	-	-		50	-	-	-	-	-	-
**5**	65	-	-		50	-	-	-	-	-	-
**6**	15	-	-		100	100	20	-	-	-	-
**7**	65	-	-		100	100	100	100	100	100	20
**8**	10	-	-		20	-	-	-	-	-	-
**9**	100	100	40		100	100	40	-	-	-	-
**10**	40	-	-		100	20	-	-	-	-	-
**11**	20	-	-		10	-	-	-	-	-	-
**12**	5	-	-		20	-	-	-	-	-	-
**13**	60	-	-		100	100	70	-	-	-	-
**14**	50	-	-		100	100	60	-	-	-	-
**15**	30	-	-		20	-	-	-	-	-	-
**16**	5	-	-		100	0	-	-	-	-	-
**17**	30	-	-		5	-	-	-	-	-	-
**18**	35	-	-		70	-	-	-	-	-	-
**19**	30	-	-		10	-	-	-	-	-	-
**20**	10	-	-		80	-	-	-	-	-	-
**21**	20	-	-		5	-	-	-	-	-	-
**22**	30	-	-		65	-	-	-	-	-	-
**23**	15	-	-		60	-	-	-	-	-	-
**24**	20	-	-		65	-	-	-	-	-	-
**25**	40	-	-		30	-	-	-	-	-	-
**31**	100	20	-		100	100	100	50	-	-	-
**37**	20	-	-		15	-	-	-	-	-	-
Fipronil	-	-	-		100	100	100	100	100	100	100

^a^—no test data.

**Table 2:** Fungicidal Activity of Compounds **1**–**38** against Phytopathogenic Fungi at 50 µg mL^−1^.

Compd.	Inhibition Rate (%)													
	*A.S* ^a^	*F.G*	*P.I*	*P.C*	*S.S*	*B.C*	*R.S*	*F.C*	*C.H*	*P.P*	*R.C*	*B.M*	*W.A*	*F.M*
**1**	16.7	3.4	5.9	17.2	10.7	7.7	6.2	11.6	16.7	26.8	11.6	16.3	12.5	27.3
**2**	16.7	44.8	35.3	41.9	62.5	34.6	35.8	41.9	46.7	91.1	89.5	55.1	47.5	57.6
**3**	55.6	56.0	57.1	36.8	53.5	44.8	38.5	53.6	47.8	62.8	94.9	63.3	55.6	50.0
**4**	44.4	68.0	57.1	31.6	41.9	37.9	30.8	50.0	47.8	81.4	93.2	76.7	63.0	45.5
**5**	33.3	16.0	7.1	15.8	10.5	24.1	11.5	21.4	21.7	41.9	69.5	16.7	22.2	13.6
**6**	16.7	37.9	23.5	38.7	44.6	34.6	35.8	34.9	43.3	57.1	83.7	51.0	45.0	48.5
**7**	12.5	10.3	23.5	25.8	10.7	11.5	12.3	16.3	30.0	44.6	23.3	16.3	22.5	21.2
**8**	16.7	41.4	17.6	32.3	14.3	11.5	6.2	14.0	16.7	26.8	17.4	14.3	27.5	18.2
**9**	33.3	40.0	28.6	31.6	53.5	27.6	32.7	39.3	47.8	76.7	88.1	70.0	37.0	50.0
**10**	55.6	56.0	50.0	31.6	58.1	51.7	32.7	57.1	52.2	58.1	94.9	66.7	51.9	68.2
**11**	44.4	24.0	10.7	10.5	11.6	27.6	17.3	21.4	17.4	51.2	67.8	20.0	25.9	13.6
**12**	44.4	36.0	14.3	26.3	18.6	13.8	9.6	25.0	30.4	46.5	62.7	33.3	33.3	13.6
**13**	38.9	28.0	28.6	26.3	16.3	27.6	19.2	25.0	30.4	97.7	69.5	40.0	48.1	22.7
**14**	44.4	24.0	28.6	31.6	20.9	58.6	7.7	14.3	43.5	60.5	71.2	30.0	22.2	18.2
**15**	33.3	36.0	21.4	68.4	16.3	44.8	11.5	10.7	17.4	51.2	67.8	20.0	25.9	13.6
**16**	27.8	20.0	21.4	26.3	14.0	10.3	11.5	21.4	17.4	55.8	67.8	20.0	33.3	18.2
**17**	38.9	16.0	21.4	26.3	10.5	20.7	21.2	17.9	30.4	32.6	47.5	20.0	33.3	13.6
**18**	33.3	32.0	7.1	15.8	11.6	20.7	9.6	14.3	30.4	51.2	62.7	20.0	33.3	22.7
**19**	66.7	64.0	64.3	73.7	83.7	75.9	46.2	60.7	60.9	88.0	93.2	70.0	66.7	63.6
**20**	55.6	36.0	42.9	26.3	14.0	10.3	11.5	39.3	47.8	76.7	84.7	66.7	59.3	50.0
**21**	33.3	31.6	36.4	27.8	19.6	47.6	16.0	37.1	40.0	44.9	87.3	50.0	44.4	50.0
**22**	22.2	31.6	18.2	19.4	53.6	23.8	33.3	37.1	30.0	51.0	87.3	44.4	29.6	46.2
**23**	27.8	12.0	14.3	26.3	10.5	10.3	9.6	7.1	26.1	67.4	59.3	3.3	37.0	18.2
**24**	11.1	12.0	10.7	26.3	11.6	17.2	9.6	25.0	30.4	67.4	71.2	13.3	14.8	9.1
**25**	55.6	44.0	57.1	52.6	90.7	89.7	57.7	60.7	69.6	84.2	94.9	66.7	59.3	63.6
**26**	44.4	31.6	54.5	61.1	82.1	47.6	30.9	48.6	60.0	51.0	92.7	55.6	48.1	53.8
**27**	22.2	31.6	27.3	19.4	26.8	23.8	18.5	34.3	35.0	65.3	89.1	47.2	40.7	53.8
**28**	16.7	18.4	27.3	27.8	53.6	14.3	6.2	14.3	5.0	6.1	58.2	22.2	14.8	26.9
**29**	11.1	5.3	4.5	27.8	17.9	19.0	6.2	31.4	25.0	26.5	72.7	27.8	14.8	30.8
**30**	31.3	45.5	21.1	27.3	33.3	12.5	37.9	16.7	45.5	56.4	66.2	28.9	21.4	27.3
**31**	8.3	15.8	20.7	24.0	5.4	31.0	6.6	8.3	9.5	13.2	27.3	29.6	7.4	17.9
**32**	11.1	21.1	27.3	22.2	23.2	14.3	6.2	17.1	15.0	8.2	56.4	19.4	18.5	30.8
**33**	31.3	27.3	31.6	40.9	29.4	18.8	22.7	36.7	50.0	89.7	78.4	44.7	21.4	40.9
**34**	43.8	31.8	15.8	4.5	33.3	18.8	18.2	10.0	27.3	66.7	20.3	31.6	14.3	18.2
**35**	12.5	27.3	15.8	4.5	11.8	18.8	12.1	6.7	18.2	46.2	37.8	15.8	10.7	18.2
**36**	37.5	36.4	31.6	27.3	49.0	31.3	30.3	56.7	50.0	79.5	78.4	68.4	57.1	54.5
**37**	44.4	44.7	50.0	55.6	81.7	47.6	34.6	54.3	40.0	22.4	89.1	61.1	59.3	65.4
**38**	44.4	47.4	50.0	55.6	37.5	71.4	67.9	65.7	65.0	42.9	98.2	63.9	70.4	61.5
Pyrimethanil	75.0	36.4	21.1	90.9	100	87.5	93.9	28.0	94.4	96.2	95.7	22.2	23.8	25.0

^a^*A.S*: *Alternaria solani*; *F.G*: *Fusarium graminearum*; *P.I*: *Phytophthora infestans*; *S.S*: *Sclerotinia sclerotiorum*; *B.C*: *Botrytis cinereal*; *R.S*: *Rhizoctonia solani*; *F.O*: *Fusarium oxysporum f.sp.cucumerinum*; *C.A*: *Cercospora arachidicola*; *P.P*: *Physalospora piricola*; *R.C*: *Rhizoctonia cerealis*; *B.M*: *Bipolaris maydis*; *W.A*: *Watermelon-anthracnose*; *F.M*: *Fusarium moniliforme*; *P.C*: *Phytophthora capsica*.

**Table 3:** Inhibitory Effects of Compounds **19** and **25** against Phytopathogenic Fungi.

Compd.	Phytopathogens	EC_50_ (μg mL^−1^)	Toxic Regression Eq	R ^b^
**19**	*S.S* ^a^	10.268	y = −2.048 + 2.025x	0.9757
	*P.P*	15.891	y = −3.842 + 3.198x	0.9778
	*R.C*	14.744	y = −4.221 + 3.612x	0.9844
	*B.C*	17.175	y = −1.600 + 1.295x	0.9762
**25**	*S.S*	7.072	y = −1.436 + 1.691x	0.9752
	*P.P*	4.604	y = −1.128 + 1.701x	0.9597
	*R.C*	13.231	y = −3.657 + 3.260x	0.9695
	*B.C*	7.050	y = −2.941 + 3.467x	0.9618

^a^*S.S*: *Sclerotinia sclerotiorum*; *B.C*: *Botrytis cinereal*; *P.P*: *Physalospora piricola*; *R.C*: *Rhizoctonia cerealis*; ^b^ R: regression coefficient.

**Table 4:** In vivo Protective Effects of Compounds **19** and **25** against *Sclerotinia sclerotiorum*.

Compd.	Concn (μg mL^−1^)	Protection Effect (%)
**19**	3000	55.7
	1500	35.2
	500	34.1
**25**	3000	83.0
	1500	35.2
	500	20.5
Carbendazim	500	100

**Table 5:** The Zebrafish Embryo Acute Toxicity of Compounds **7** and **25**.

Compd.	Time (h)	LC_50_ (μg mL^−1^)	Toxic Regression Eq	R	95% Confidence Limits
**7**	96	378.387	y = −4.378 + 1.698x	0.9617	325.159–369.971
**25**	24	38.187	y = −2.193 + 1.387x	0.8718	23.899–67.569
	48	30.435	y = −2.142 + 1.444x	0.9154	18.065–48.014
	72	26.886	y = −2.274 + 1.591x	0.9508	16.271–39.781
	96	21.668	y = −2.131 + 1.595x	0.9762	11.777–31.651

## Supplementary Materials

The following are available online. Figures S1–S101.
