# Supplementary material for: Investigation of Novel Pesticides with Insecticidal and Antifungal Activities: Design, Synthesis and SAR Studies of Benzoylpyrimidinylurea Derivatives
Source: Molecules. 2018 Aug 31;23(9):2203. doi: 10.3390/molecules23092203 (PMC6225173; doi:10.3390/molecules23092203)
Supplement: Supplementary file 1 [file molecules-23-02203-s001.pdf]

Electronic Supporting Information (ESI) for:

Investigation of novel pesticides with insecticidal and antifungal activity: design, synthesis and SAR studies of novel benzoyl pyrimidinylurea derivatives

Content	Page
^{1}H-NMR and ^{13}C-NMR Spectra for compounds **Iap**, **32a**, **1-38**	2-52
Photos for Protective activity of compounds agains *Sclerotinia sclerotiorum* on leaves of *Brassica olerace*	53

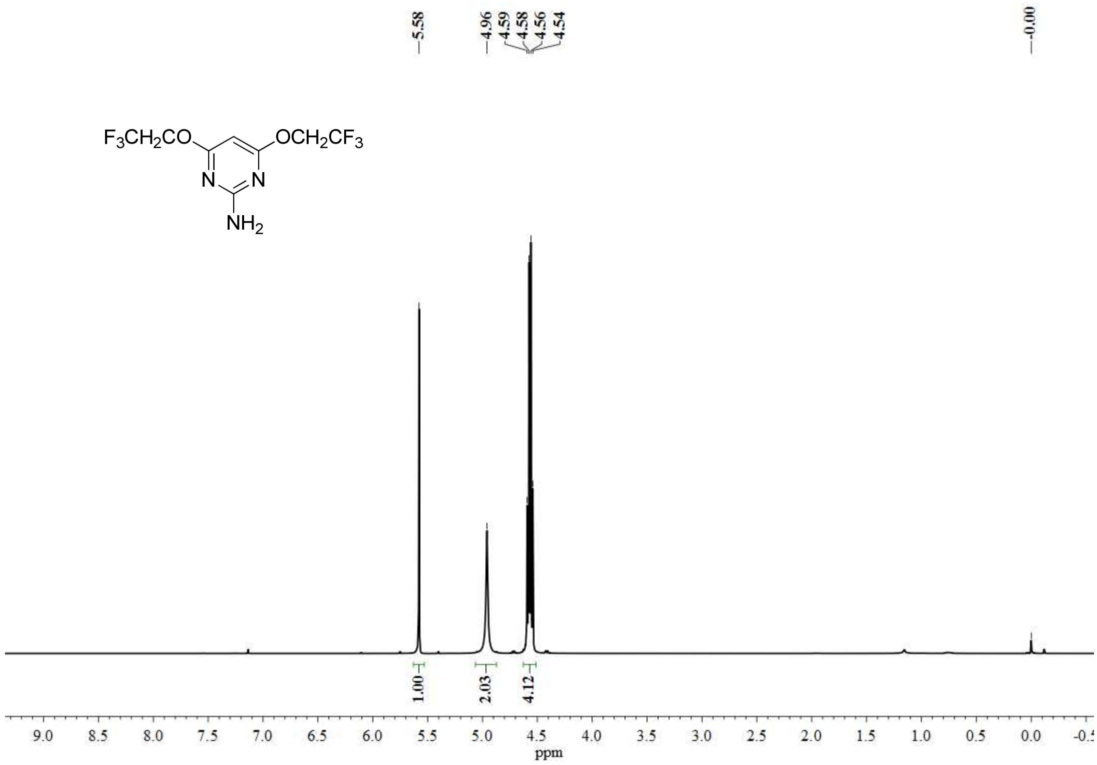

Figure S1 ^{1}H NMR spectra of compound Ia

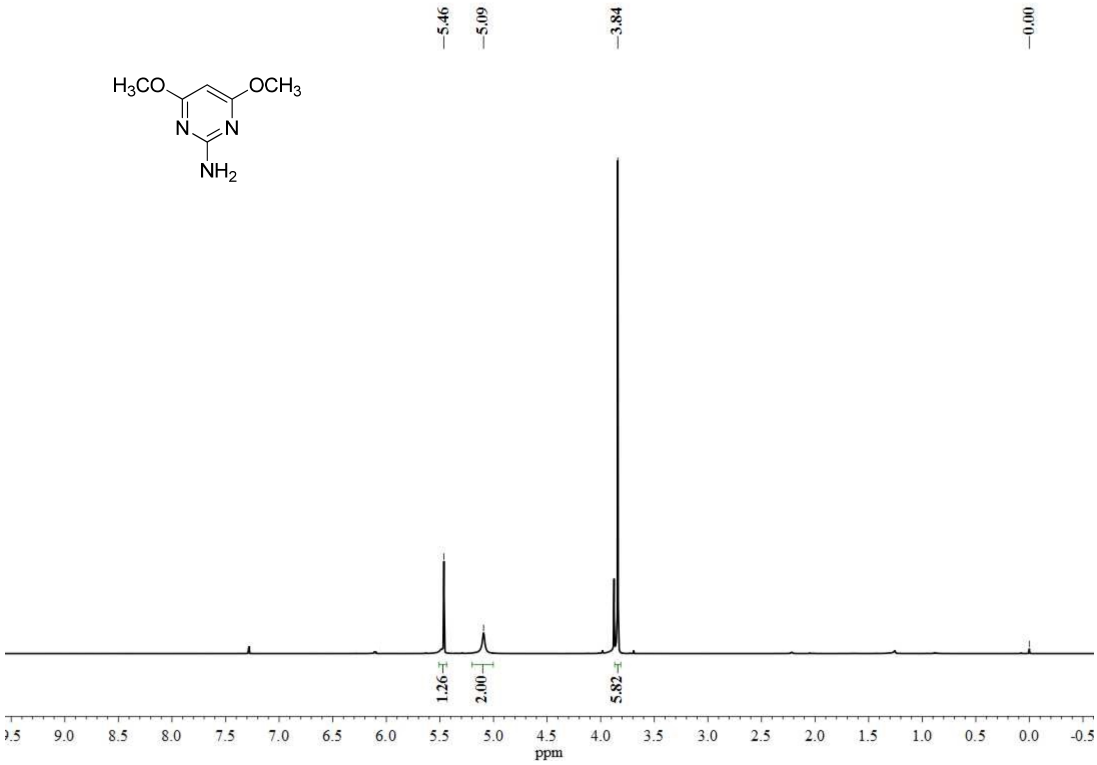

Figure S2 ^{1}H NMR spectra of compound Ib

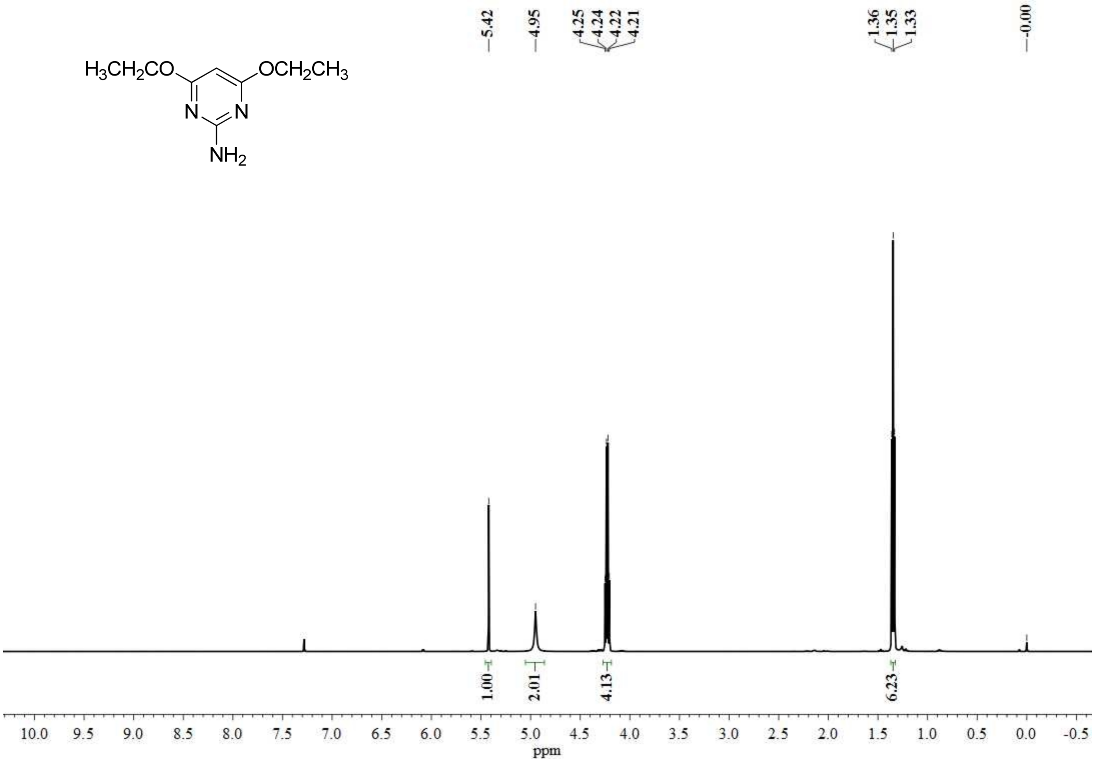

Figure S3 ^{1}H NMR spectra of compound Ic

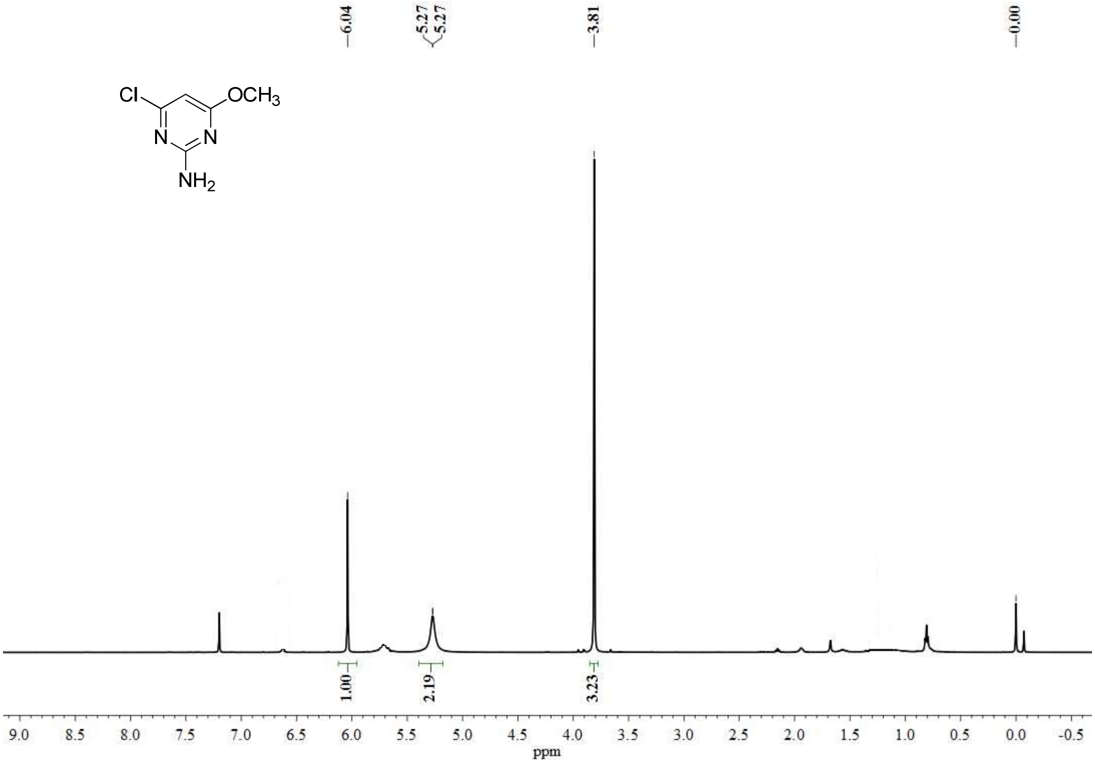

Figure S4 ^{1}H NMR spectra of compound Id

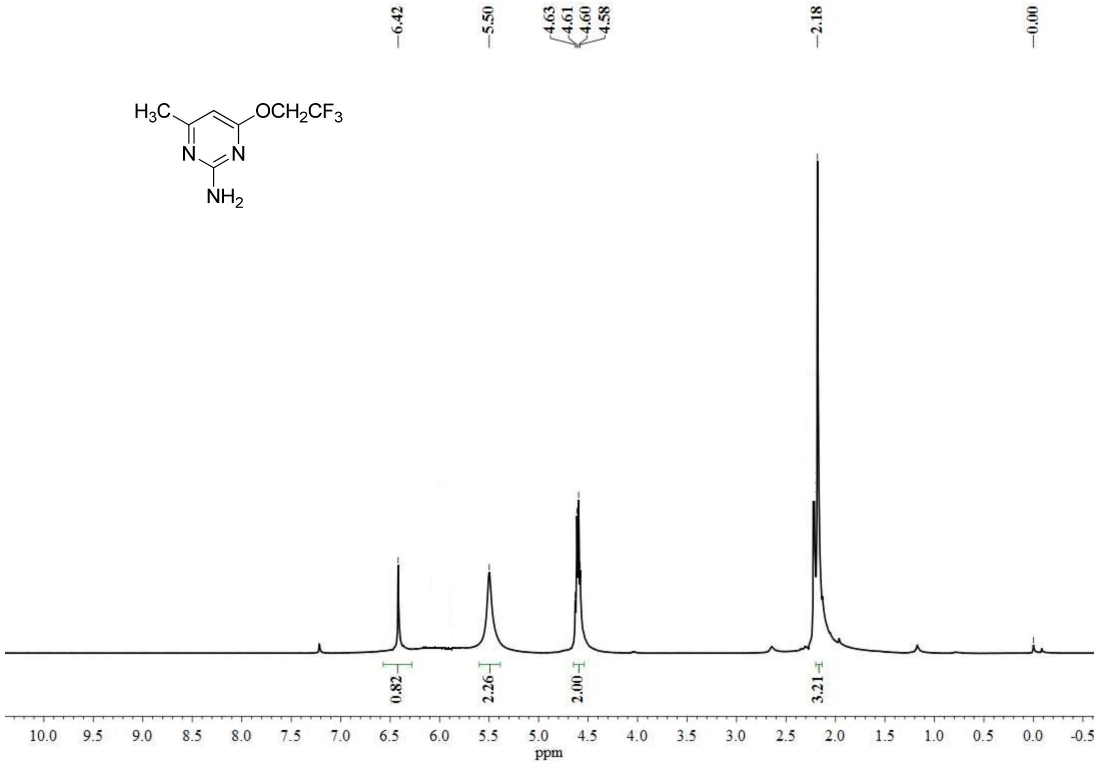

Figure S5 ^{1}H NMR spectra of compound Ie

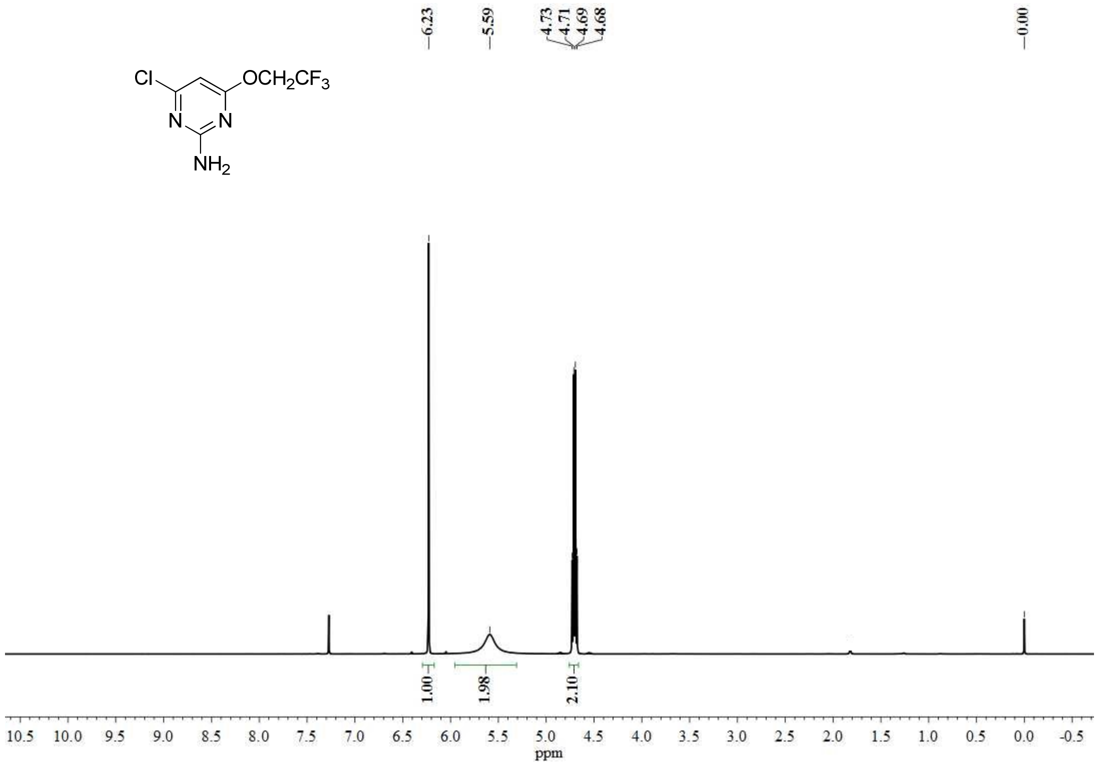

Figure S6 ^{1}H NMR spectra of compound If

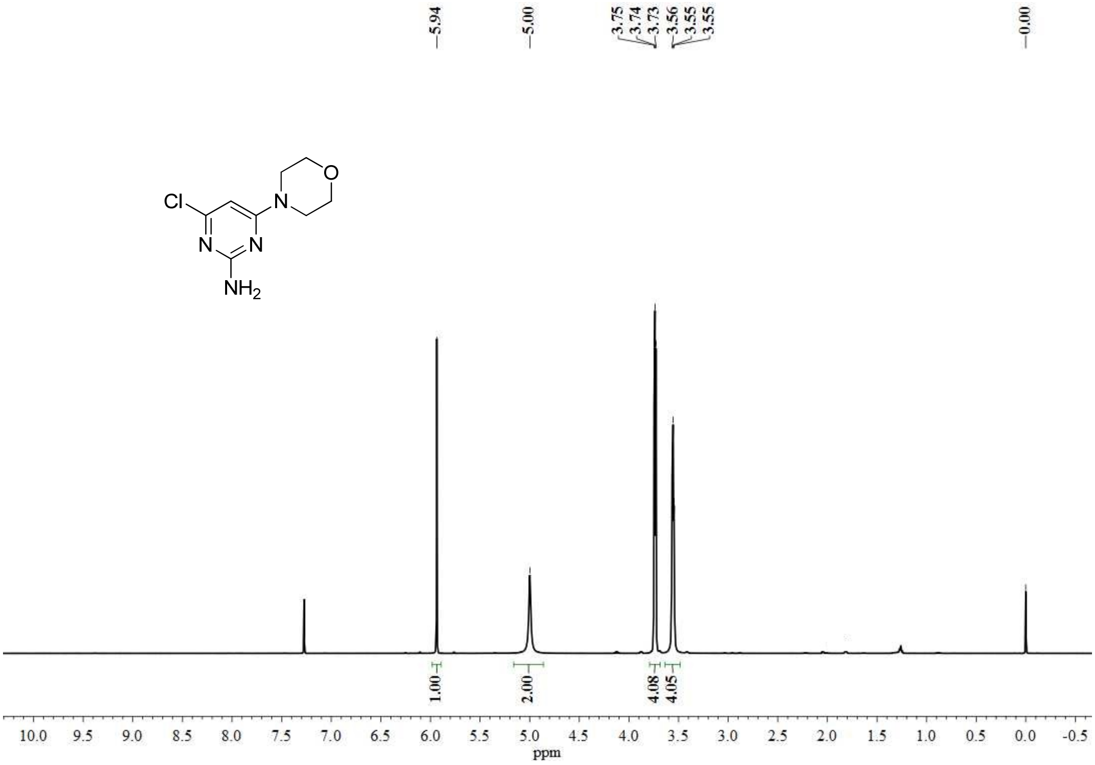

Figure S7 ^{1}H NMR spectra of compound Ig

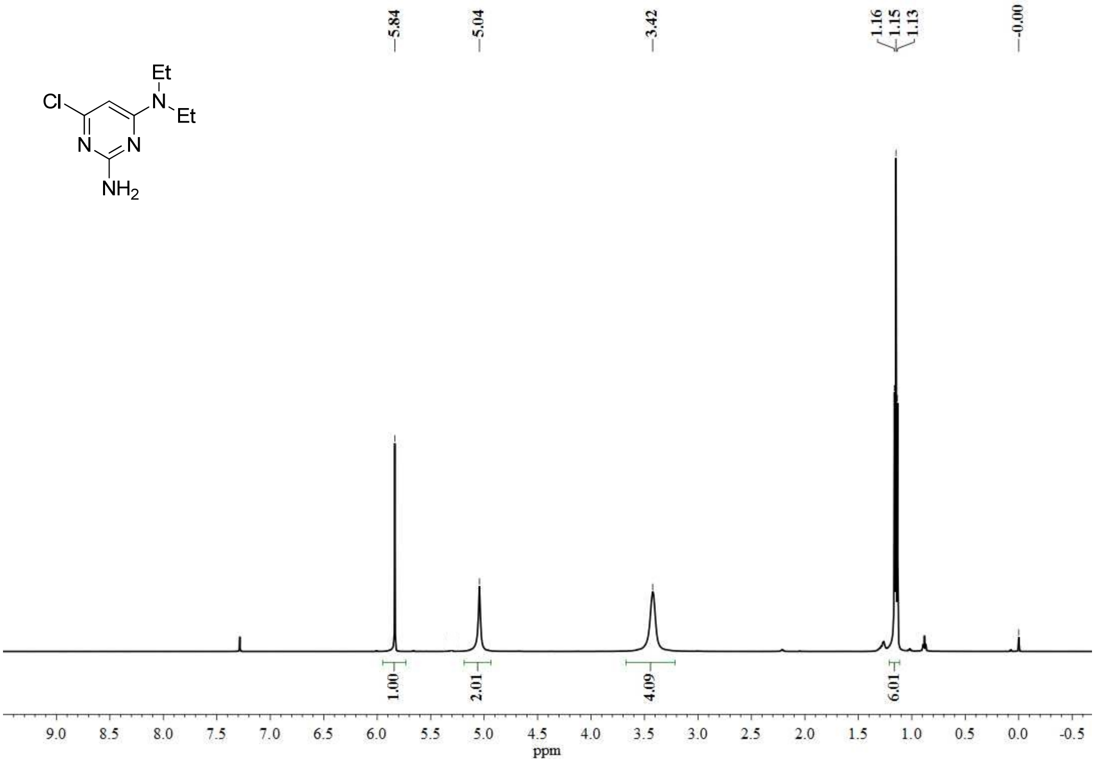

Figure S8 ^{1}H NMR spectra of compound Ih

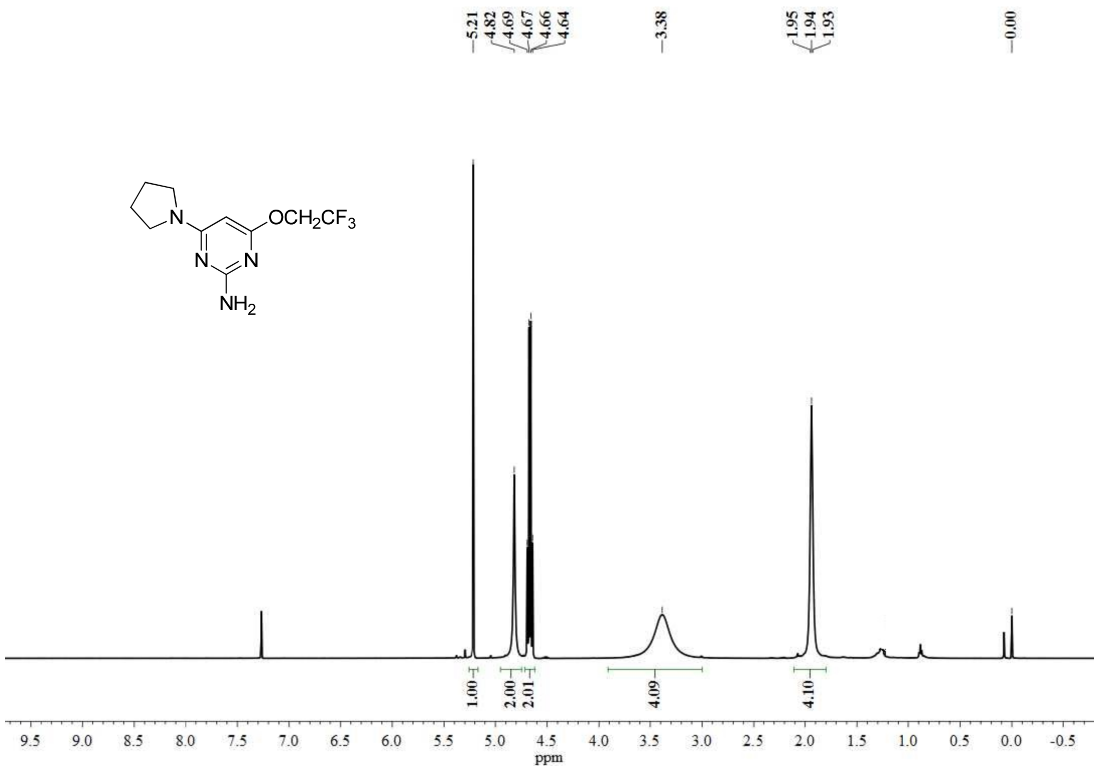

Figure S9 ^{1}H NMR spectra of compound Ii

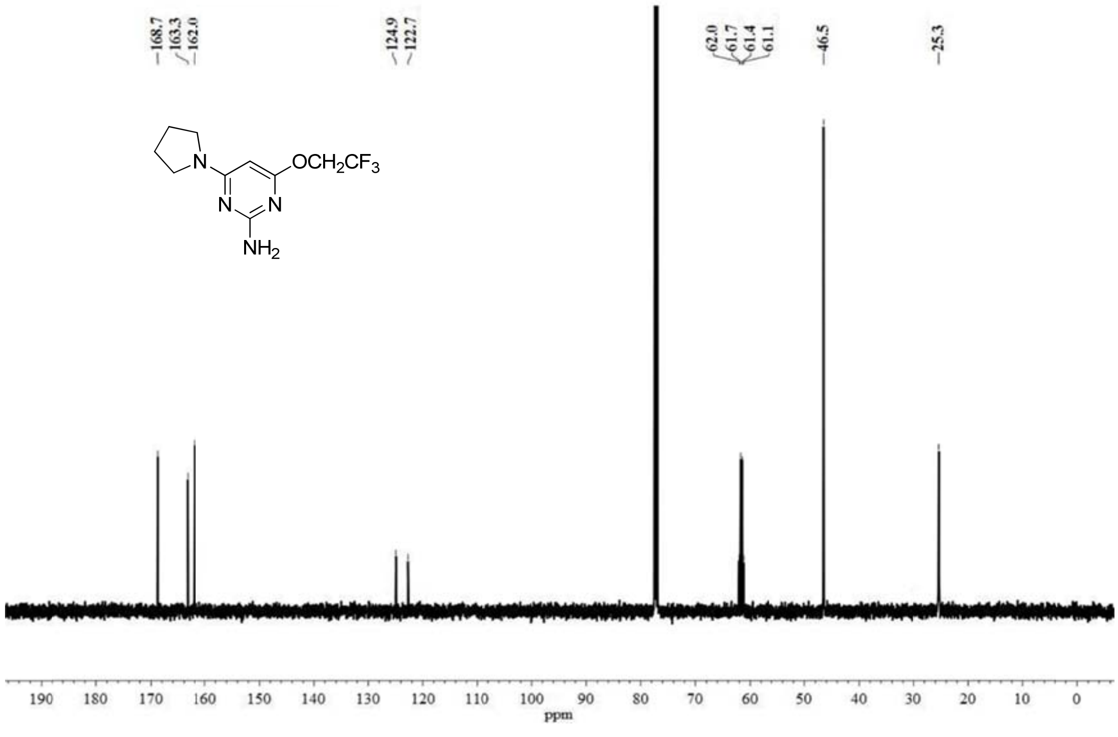

Figure S10 ^{13}C NMR spectra of compound Ii

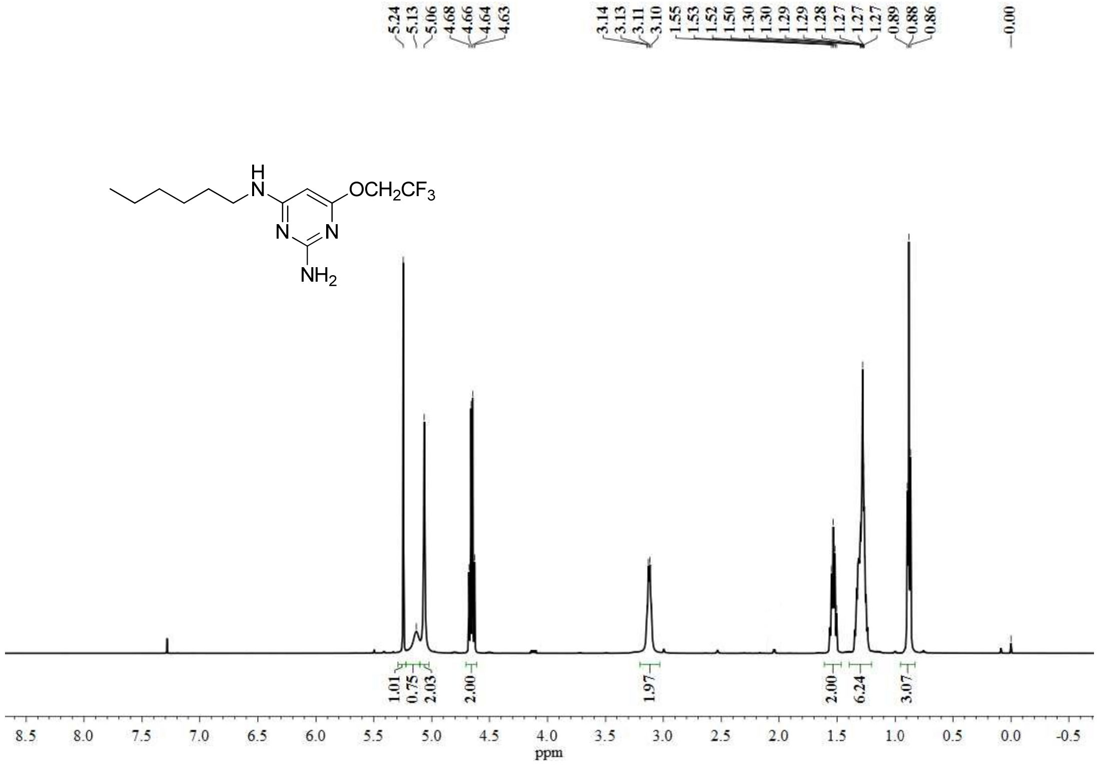

Figure S11 ^{1}H NMR spectra of compound Ij

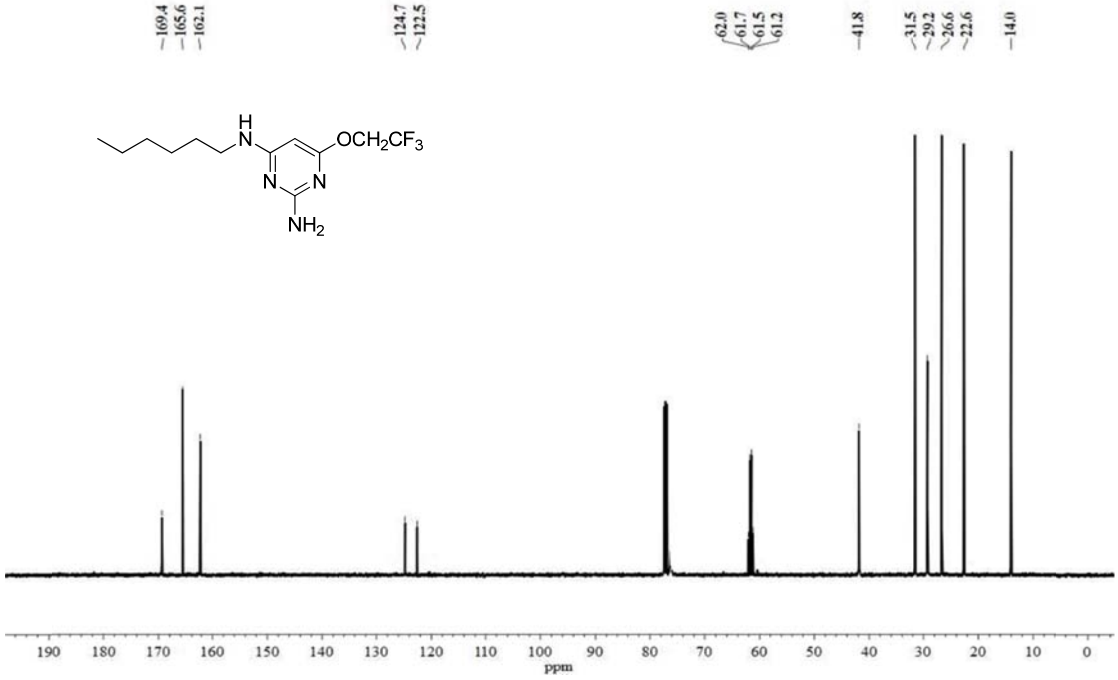

Figure S12 ^{13}C NMR spectra of compound Ij

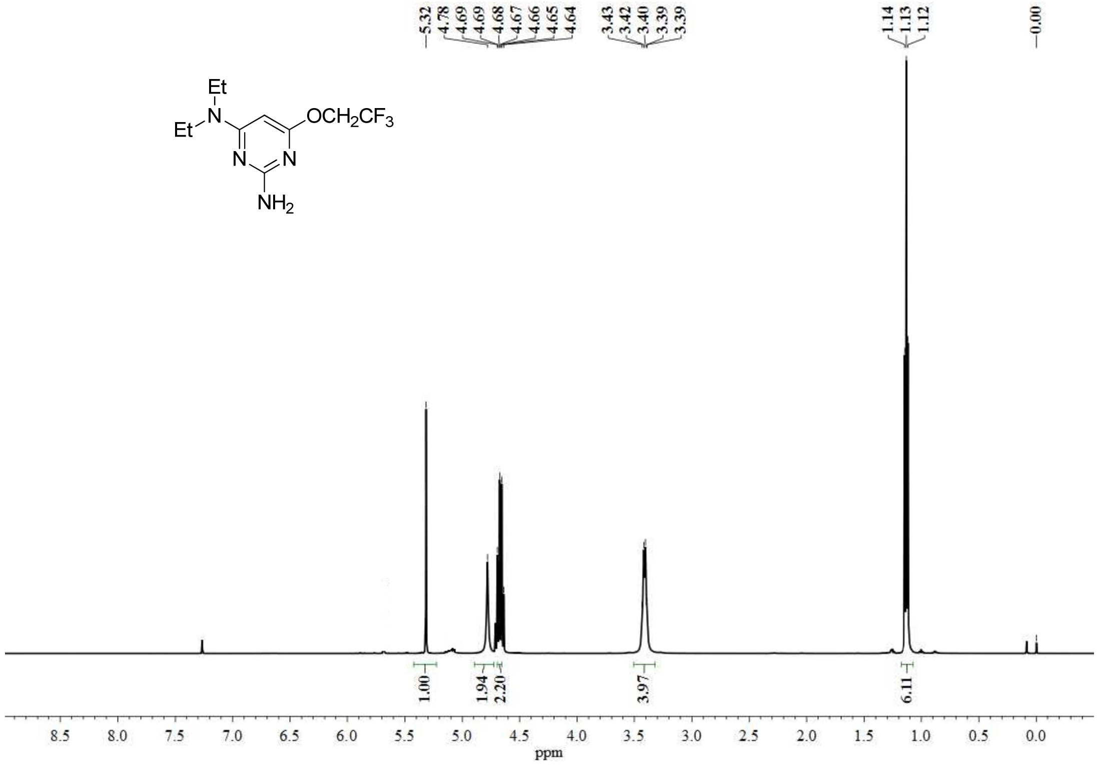

Figure S13 ^{1}H NMR spectra of compound Ik

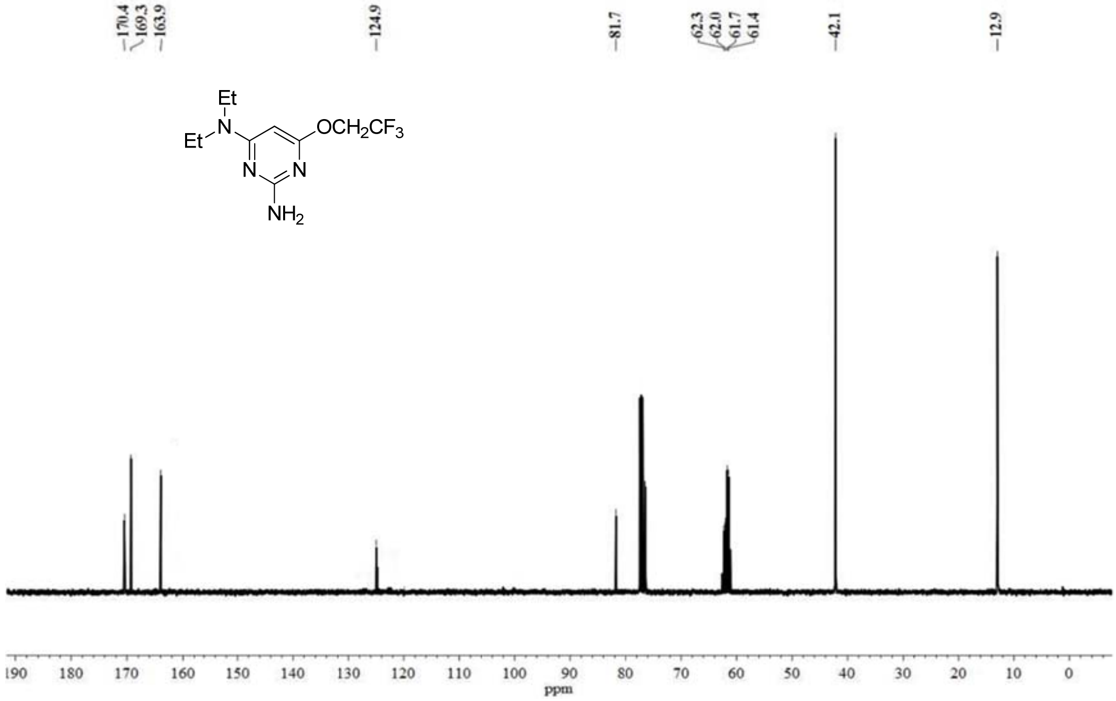

Figure S14 ^{13}C NMR spectra of compound Ik

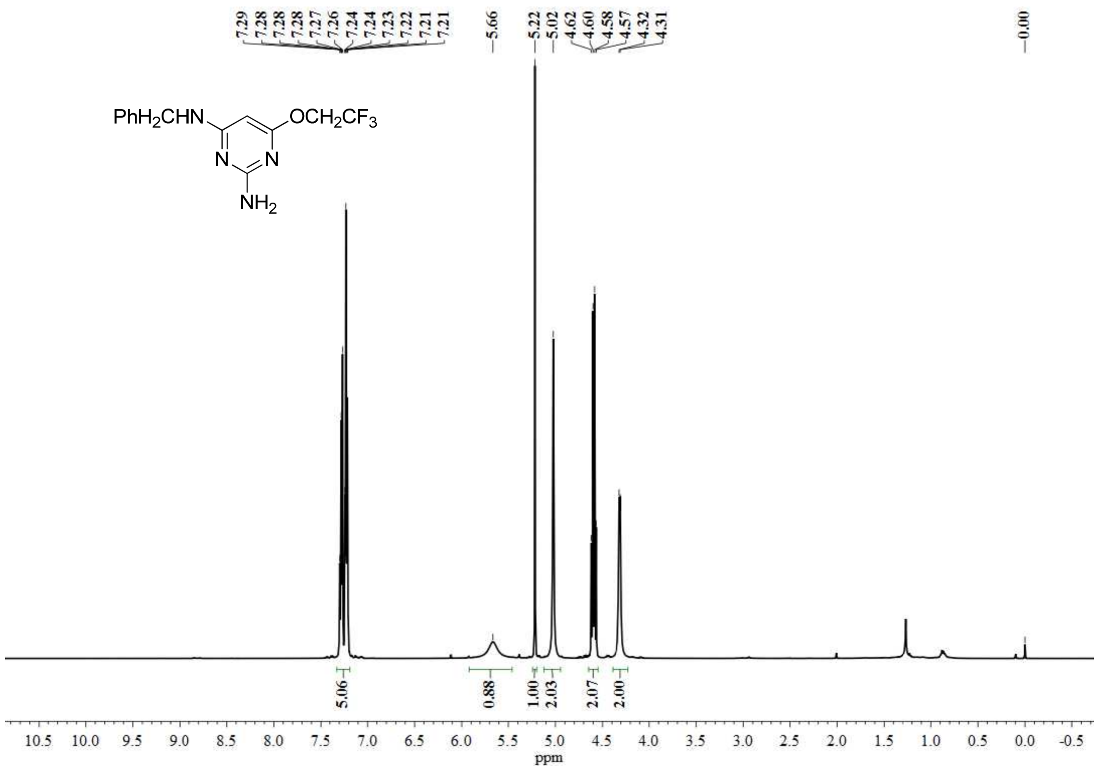

Figure S15 ^{1}H NMR spectra of compound Il

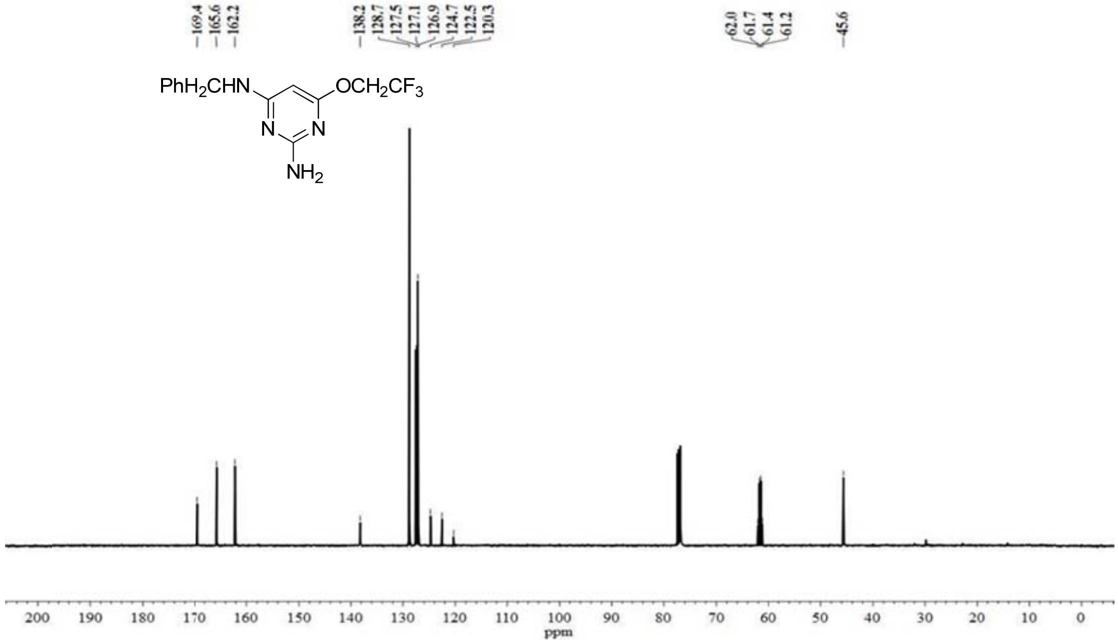

Figure S16 ^{13}C NMR spectra of compound Il

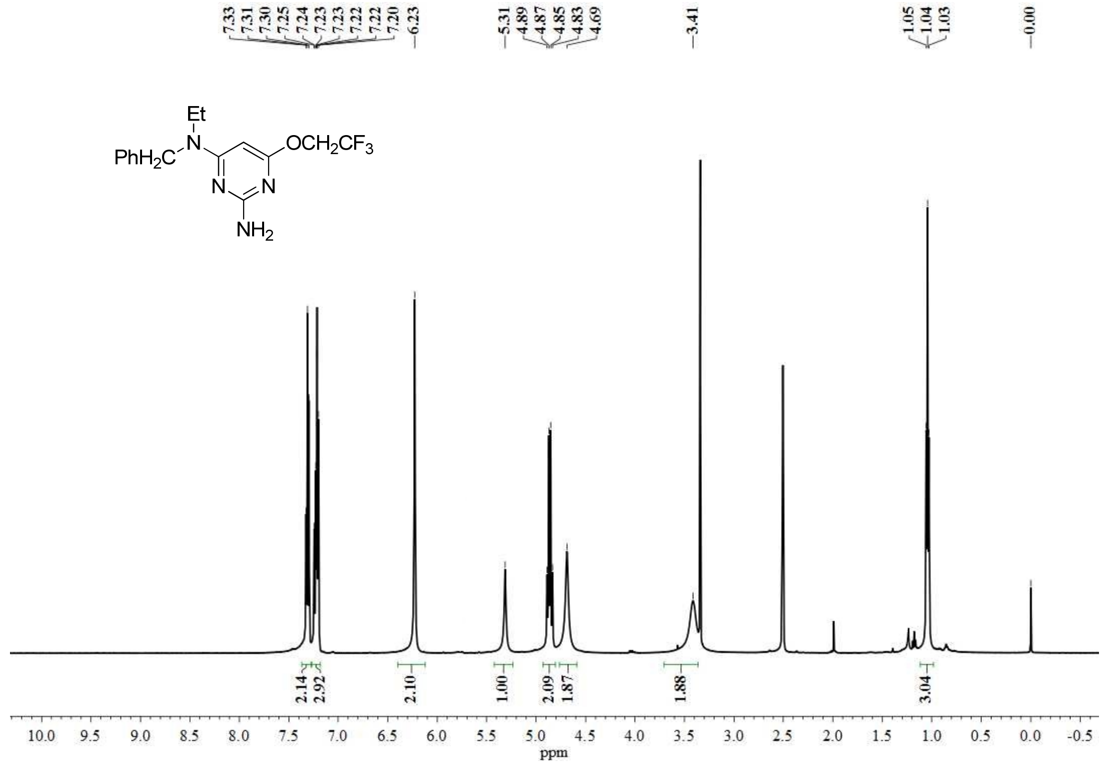

Figure S17 ^{1}H NMR spectra of compound Im

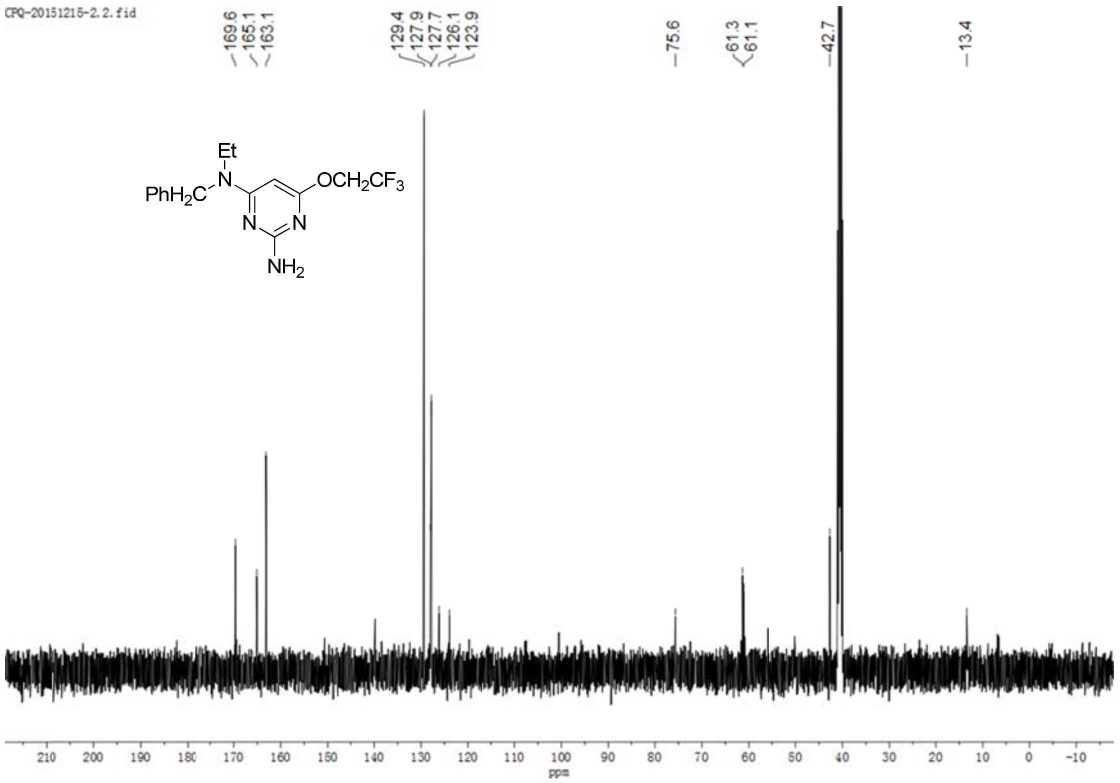

Figure S18 ^{13}C NMR spectra of compound Im

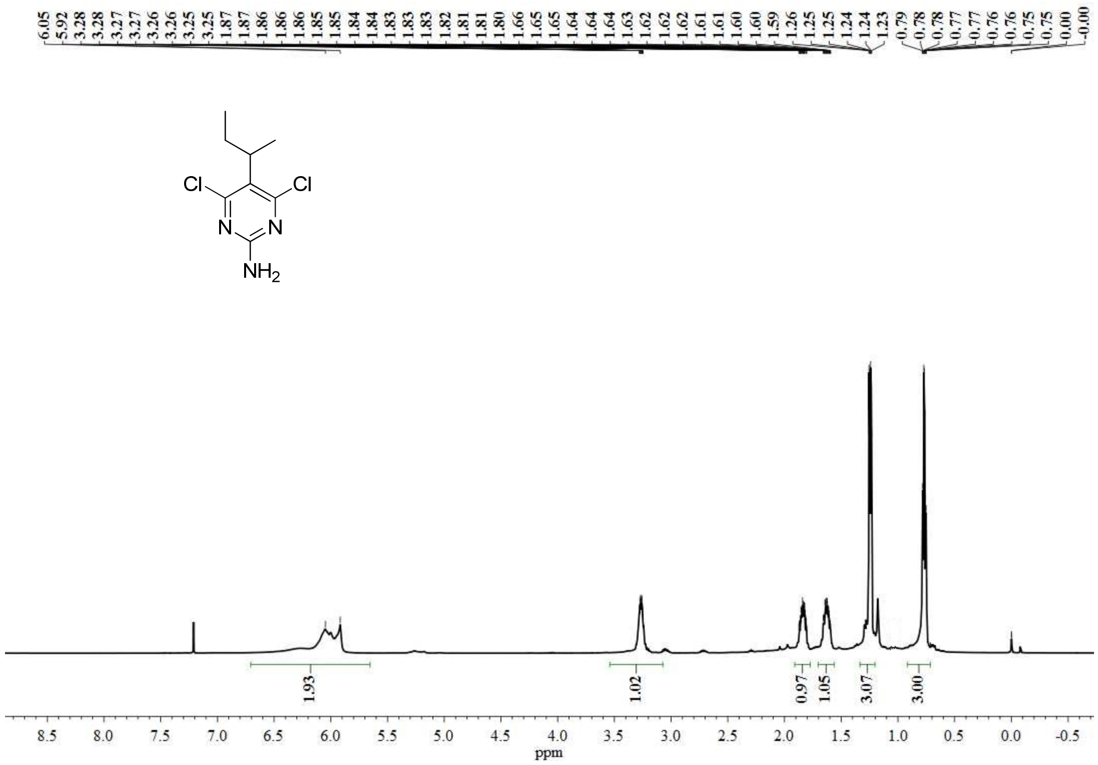

Figure S19 ^{1}H NMR spectra of compound In

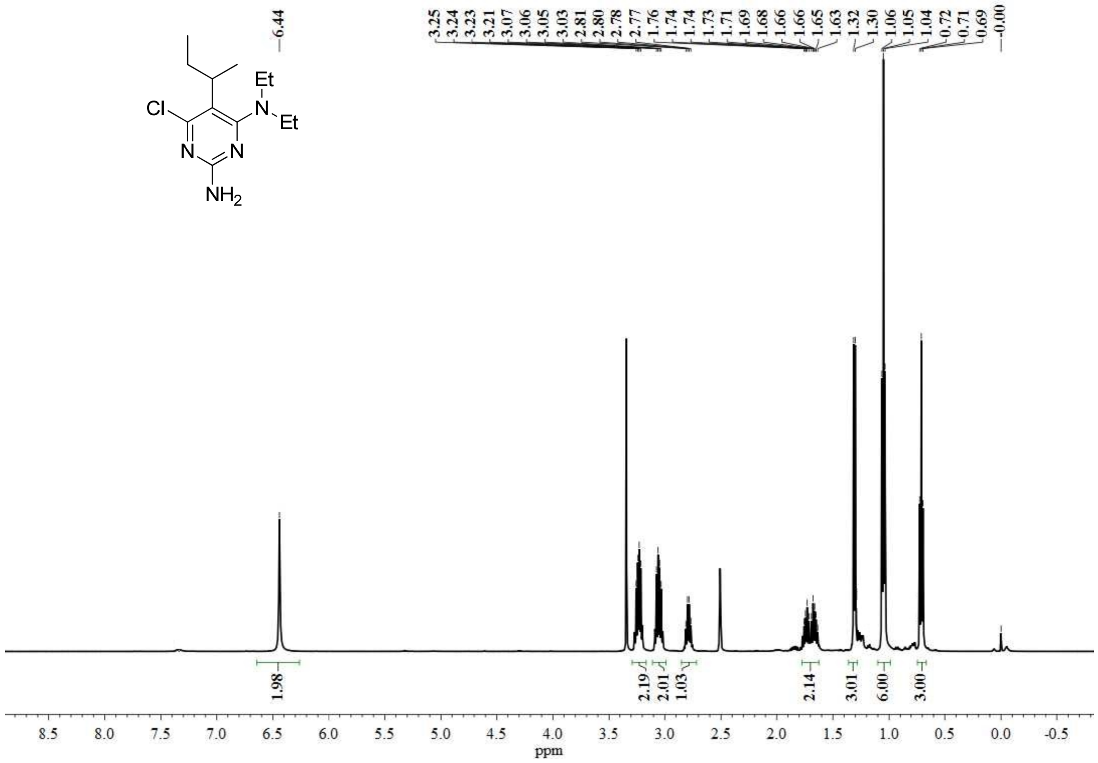

Figure S20 ^{1}H NMR spectra of compound Io

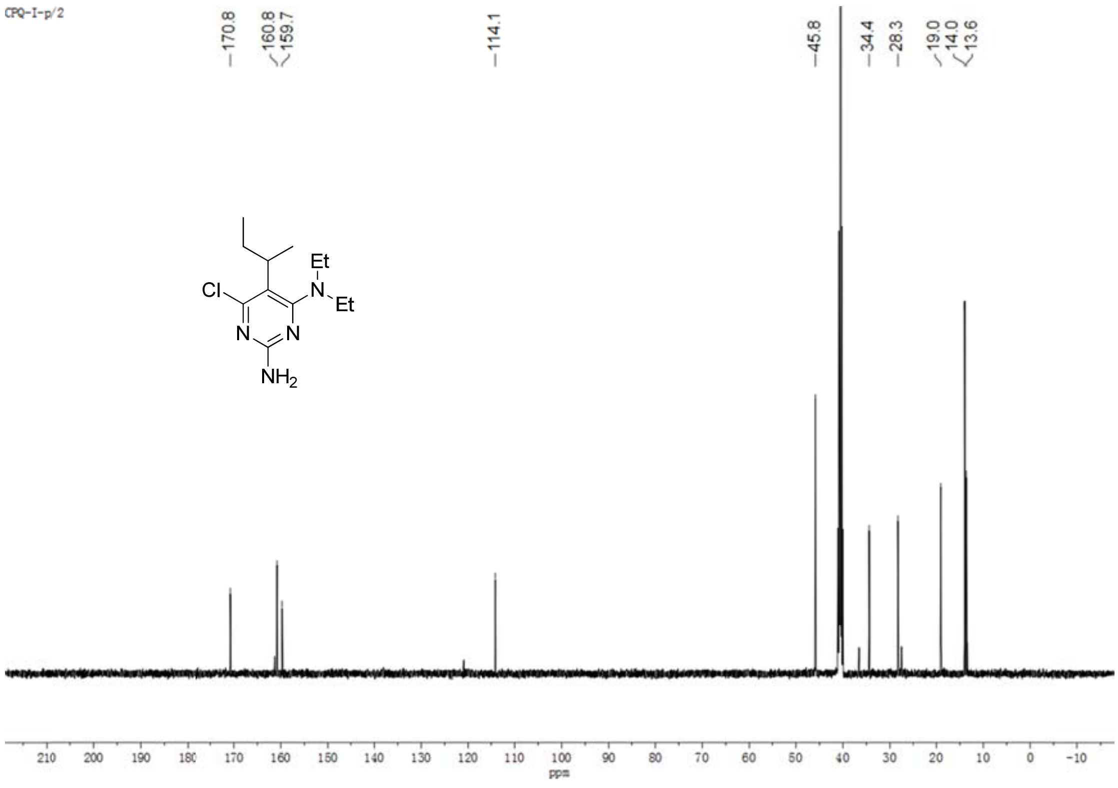

Figure S21 ^{13}C NMR spectra of compound Io

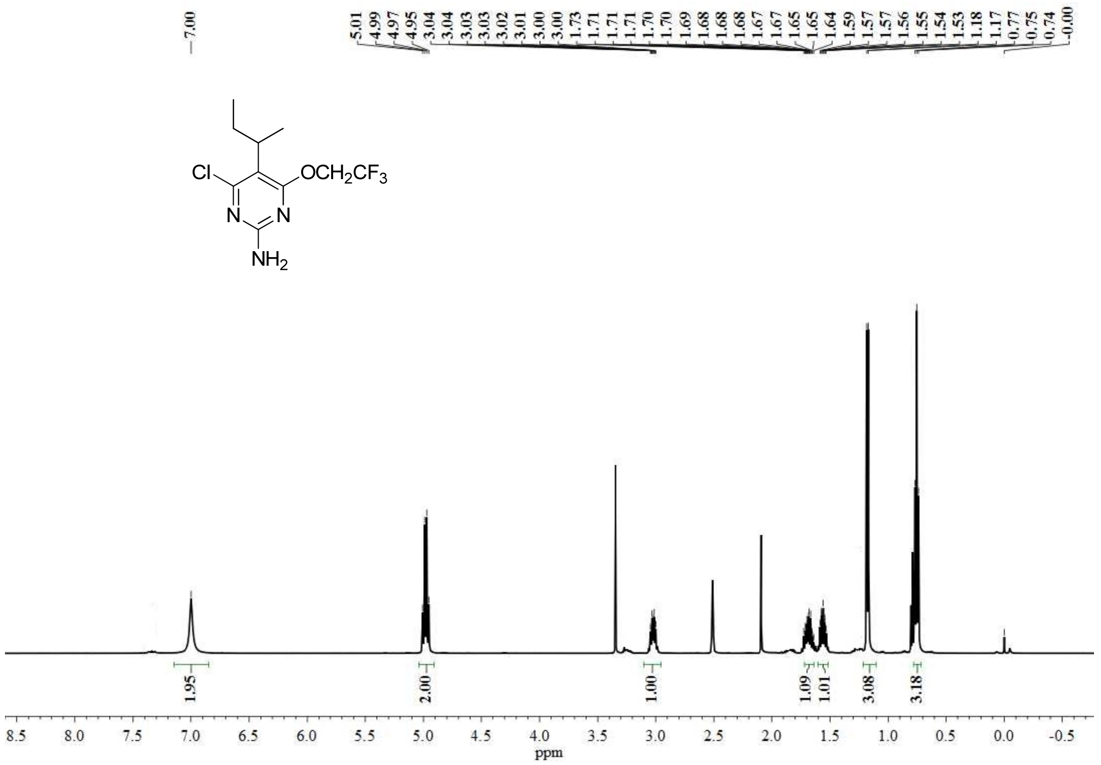

Figure S22 ^{1}H NMR spectra of compound Ip

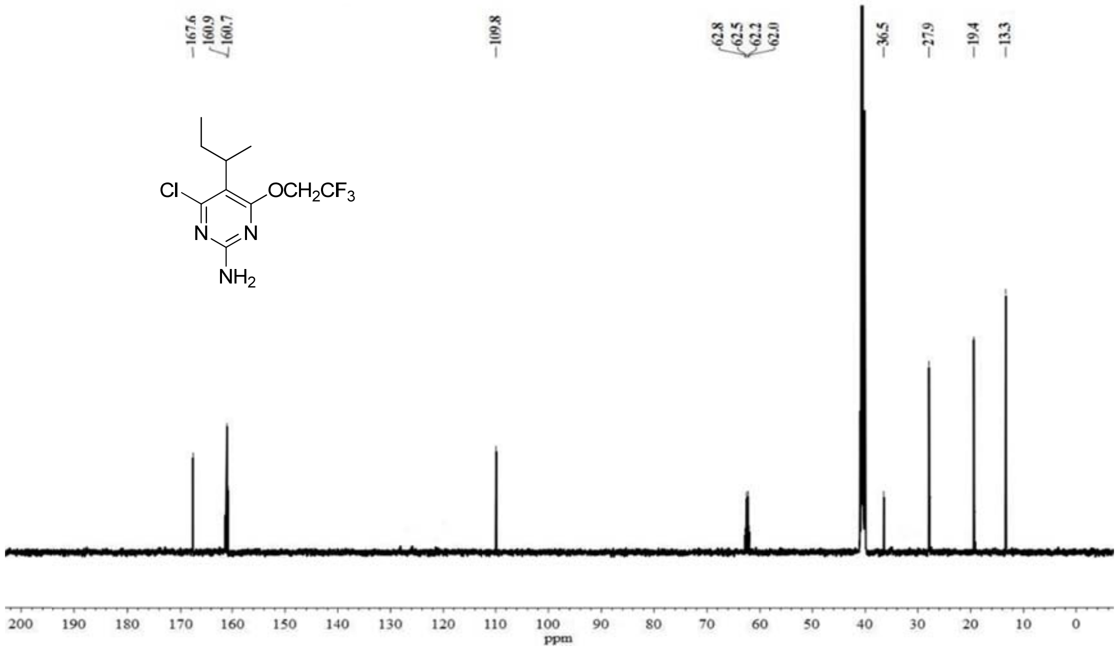

Figure S23 ^{13}C NMR spectra of compound Ip

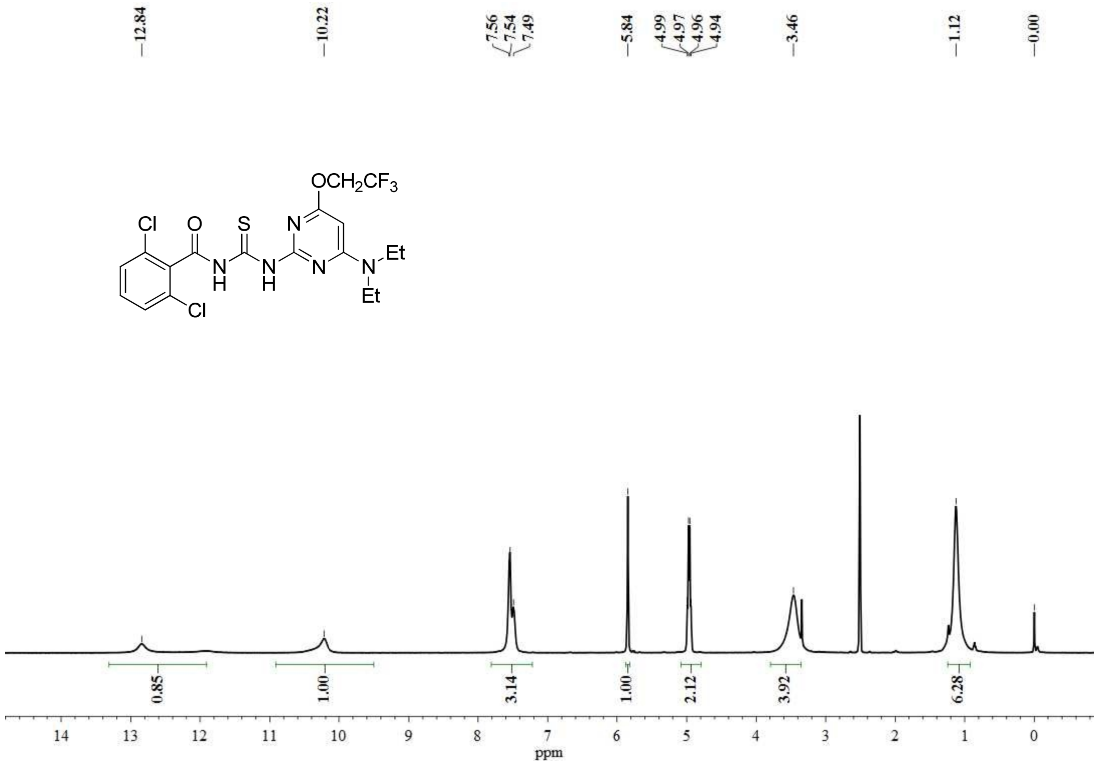

Figure S24 ^{1}H NMR spectra of compound 32a

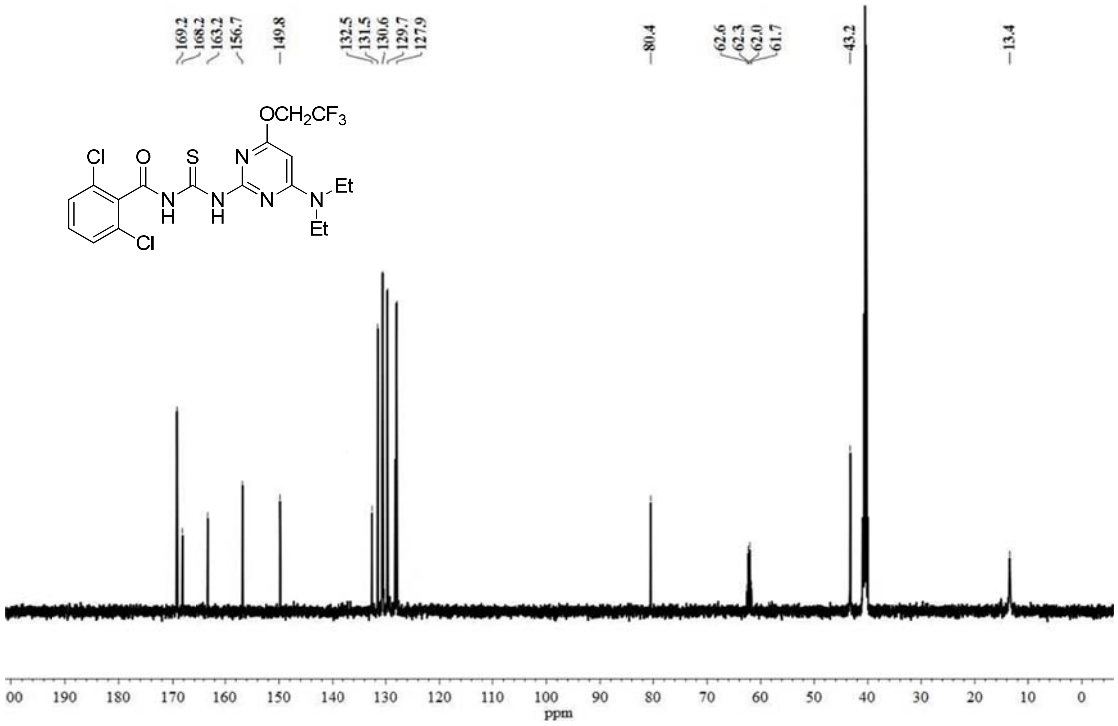

Figure S25 ^{13}C NMR spectra of compound 32a

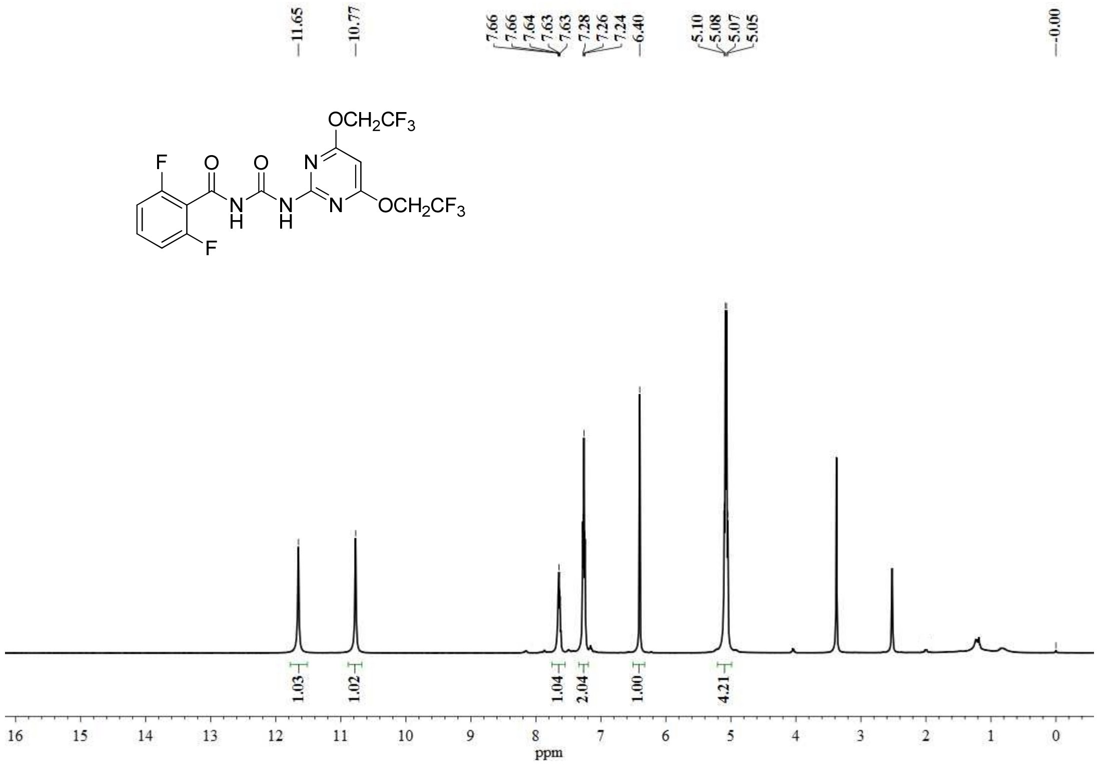

Figure S26 ^{1}H NMR spectra of compound 1

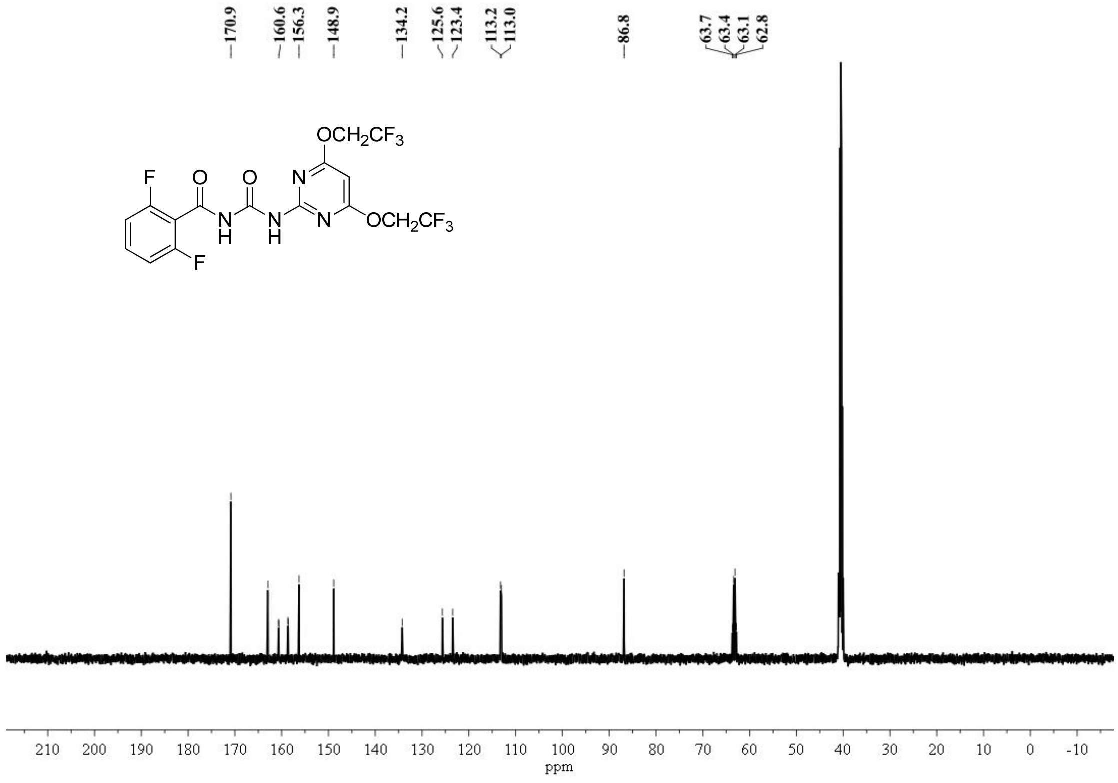

Figure S27 ^{13}C NMR spectra of compound 1

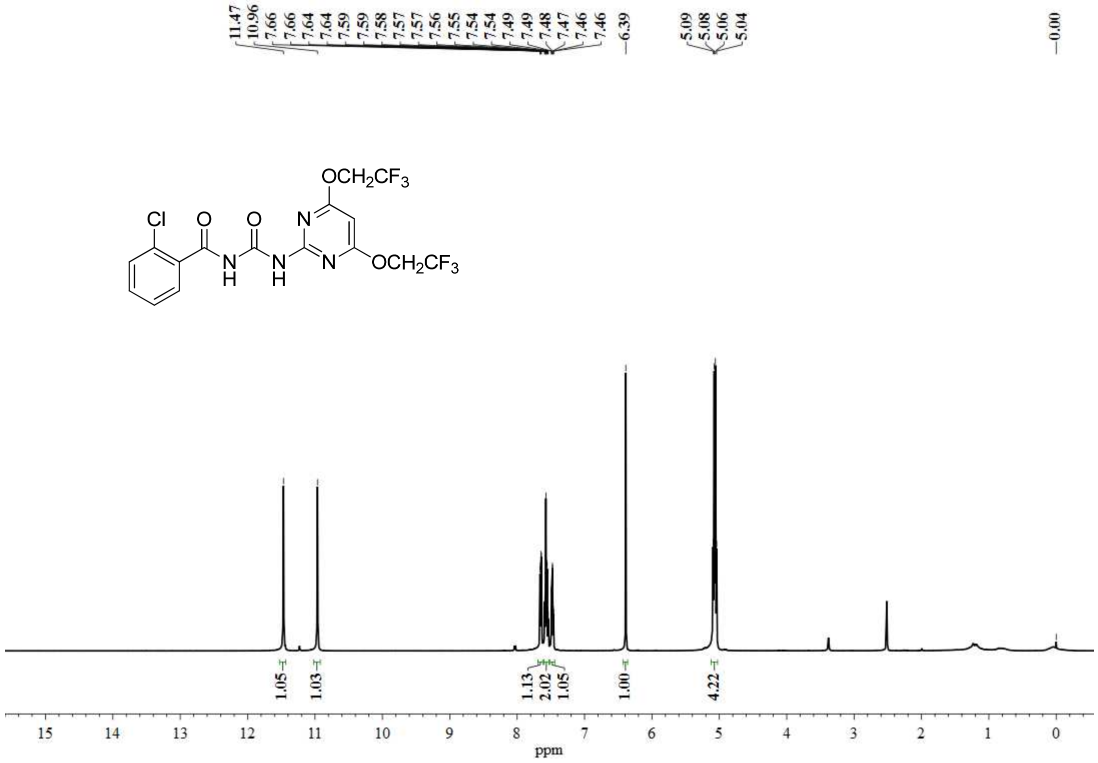

Figure S28 ^{1}H NMR spectra of compound 2

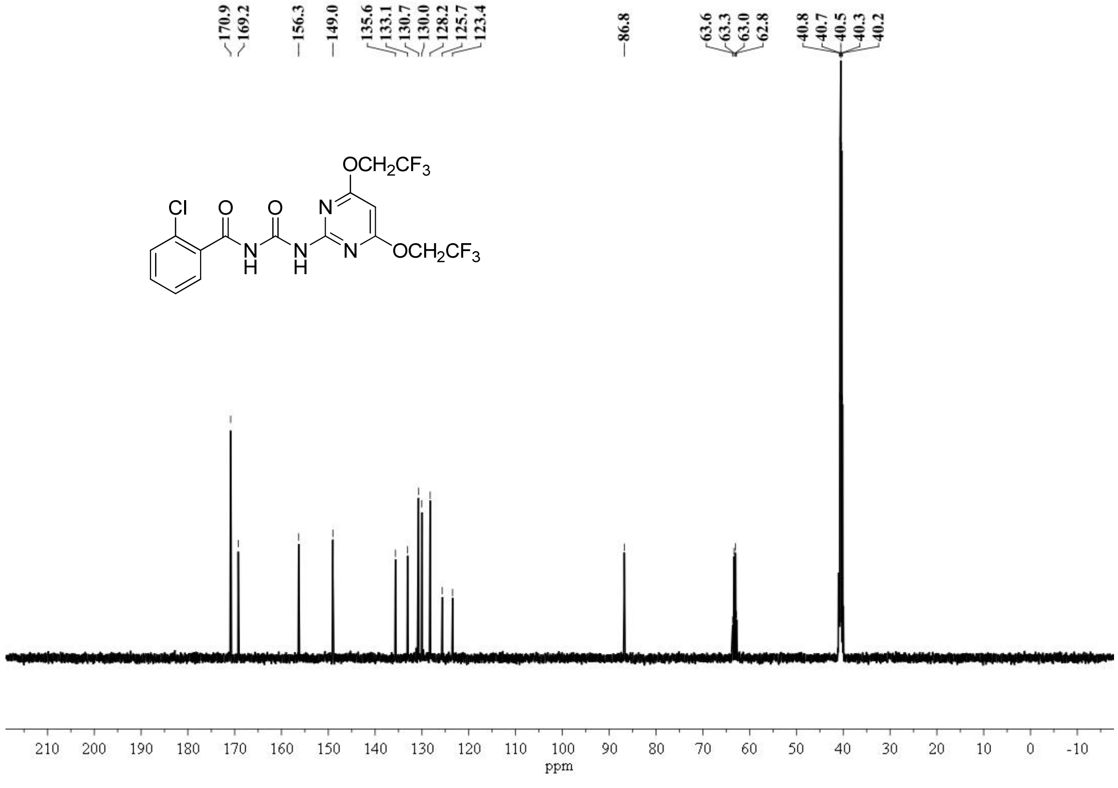

Figure S29 ^{13}C NMR spectra of compound 2

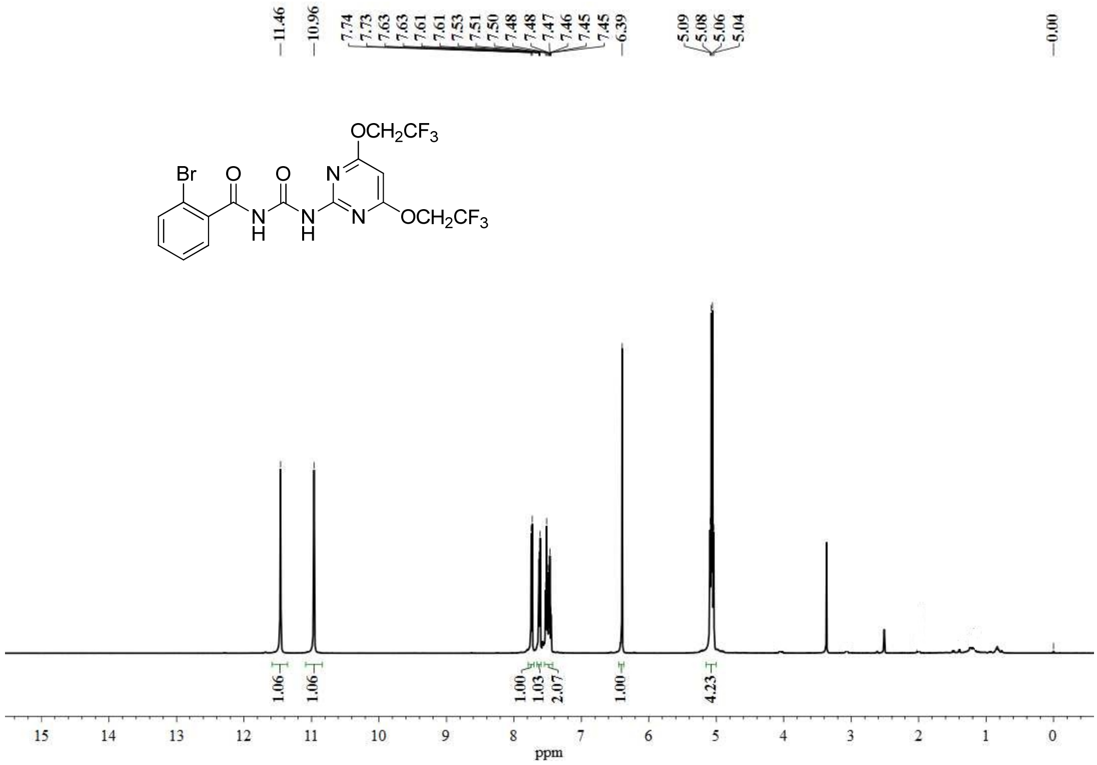

Figure S30 ^{1}H NMR spectra of compound 3

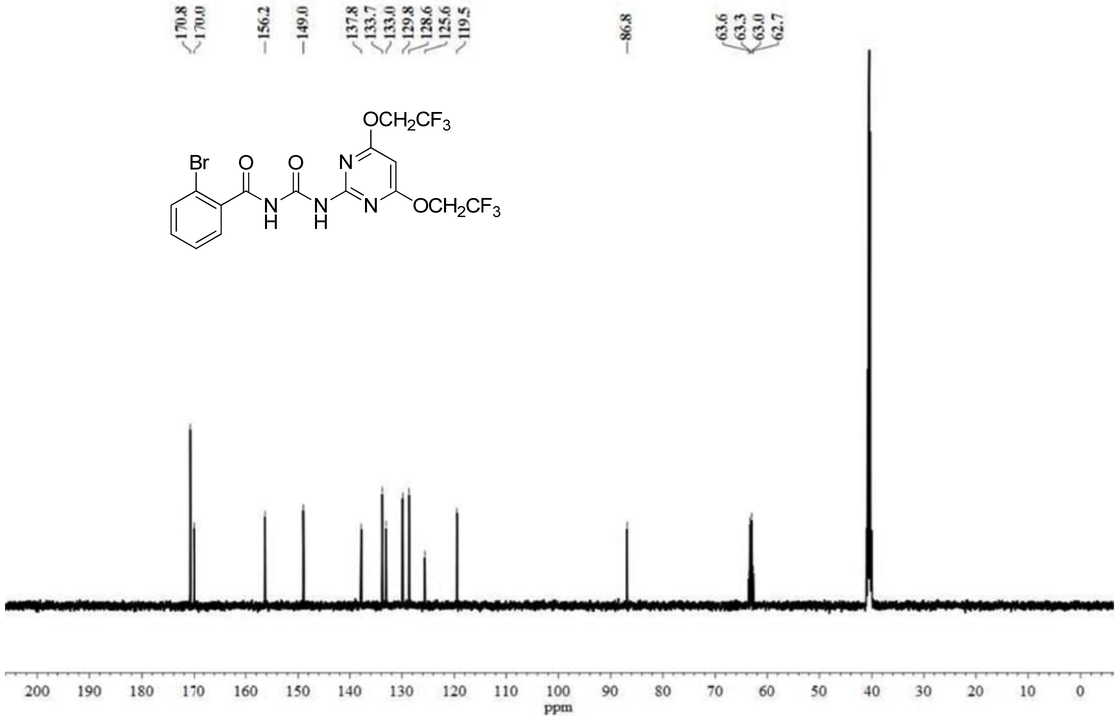

Figure S31 ^{13}C NMR spectra of compound 3

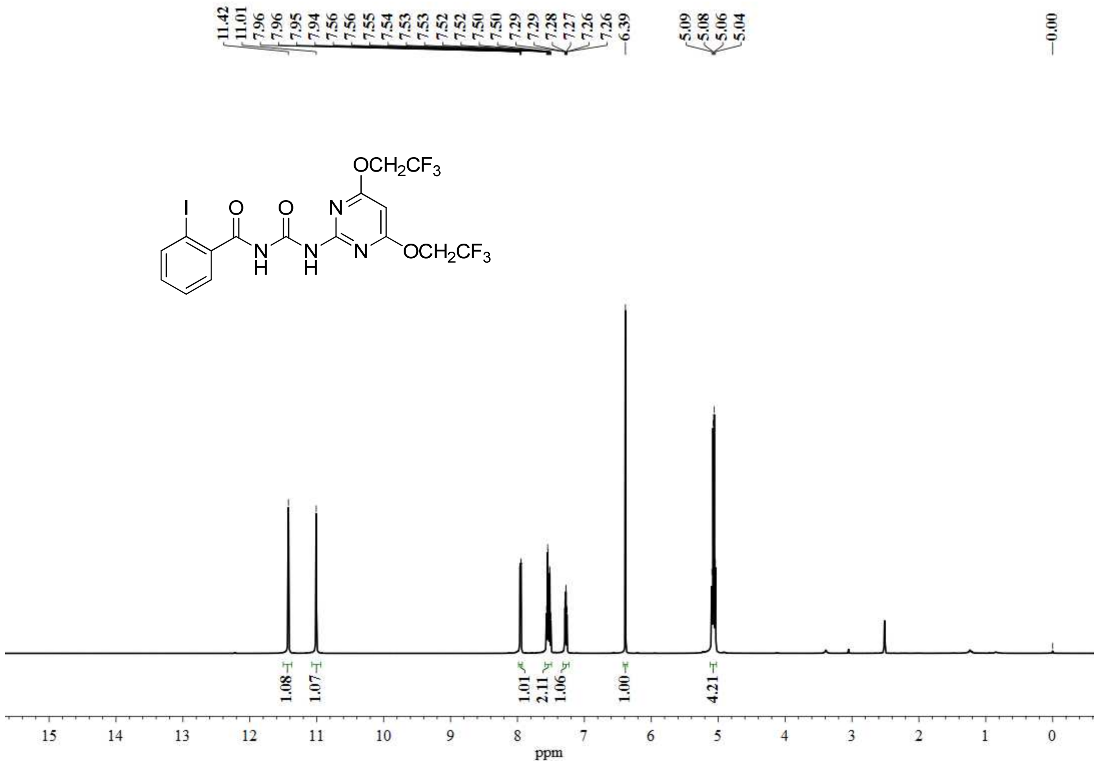

Figure S32 ^{1}H NMR spectra of compound 4

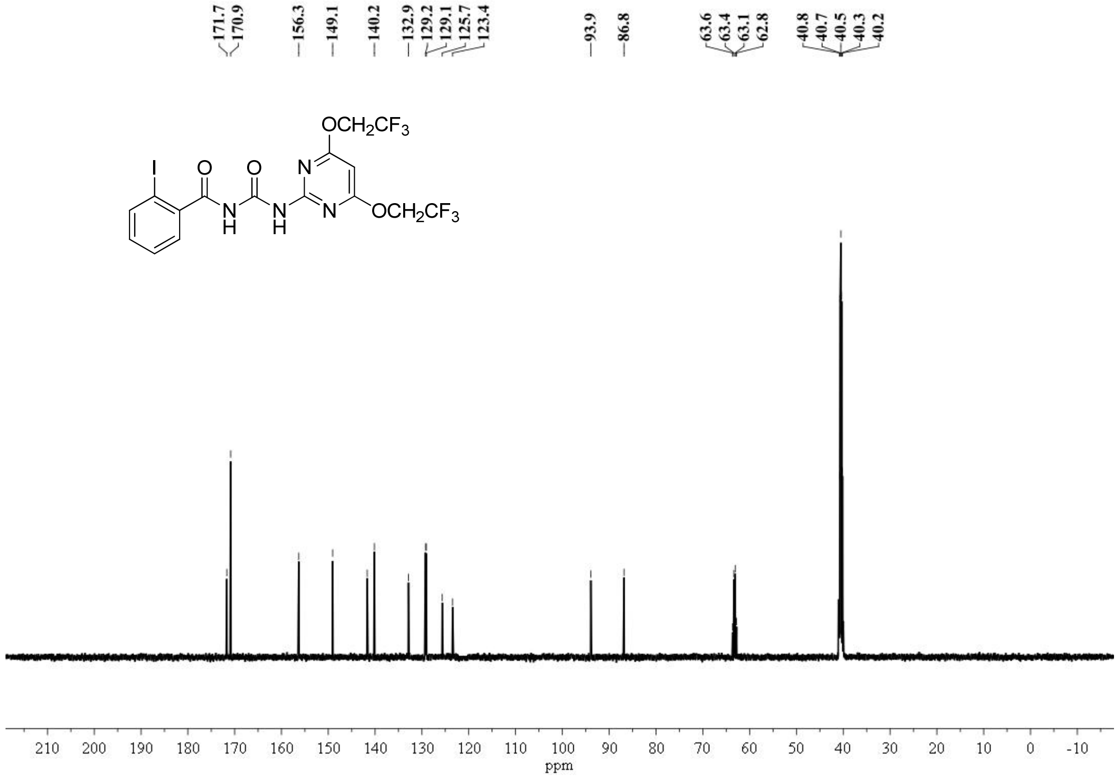

Figure S33 ^{13}C NMR spectra of compound 4

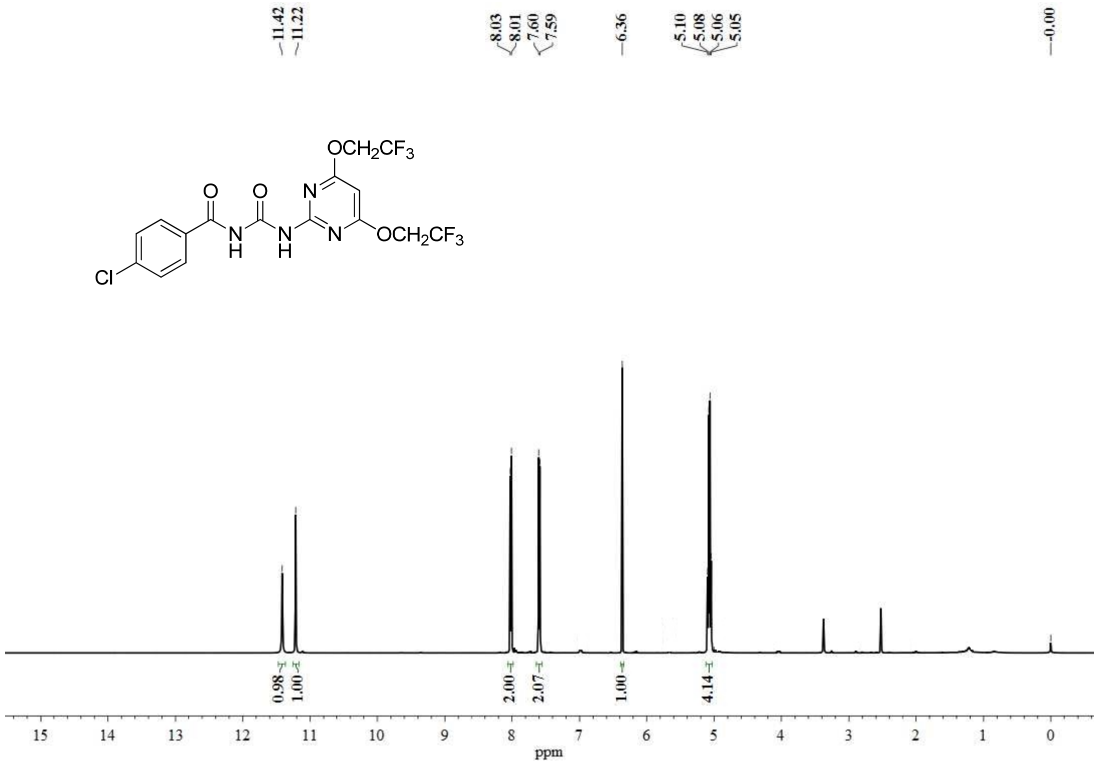

Figure S34 ^{1}H NMR spectra of compound 5

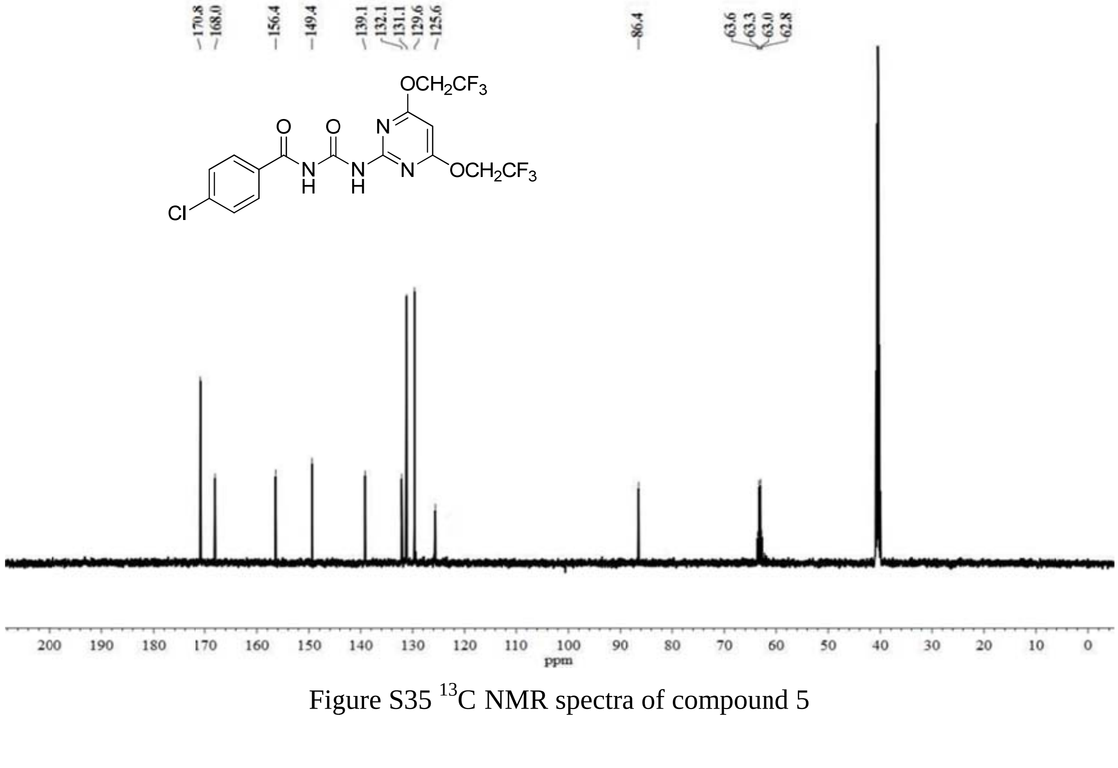

Figure S35 ^{13}C NMR spectra of compound 5

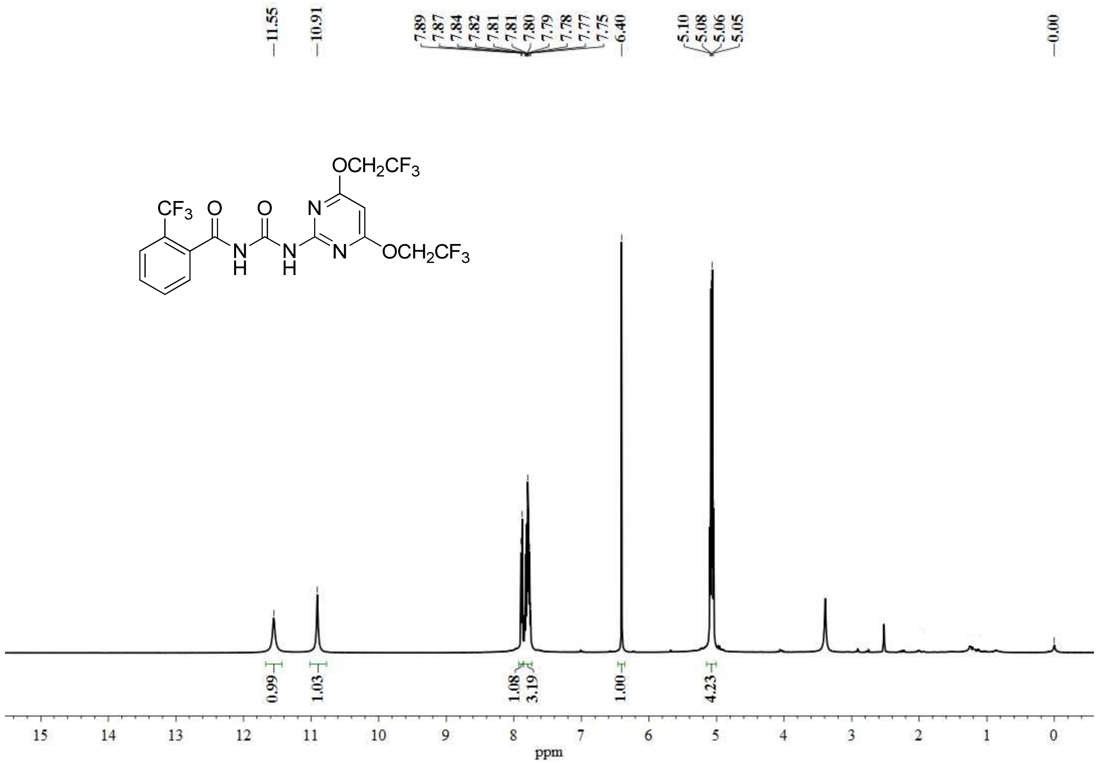

Figure S36 ^{1}H NMR spectra of compound 6

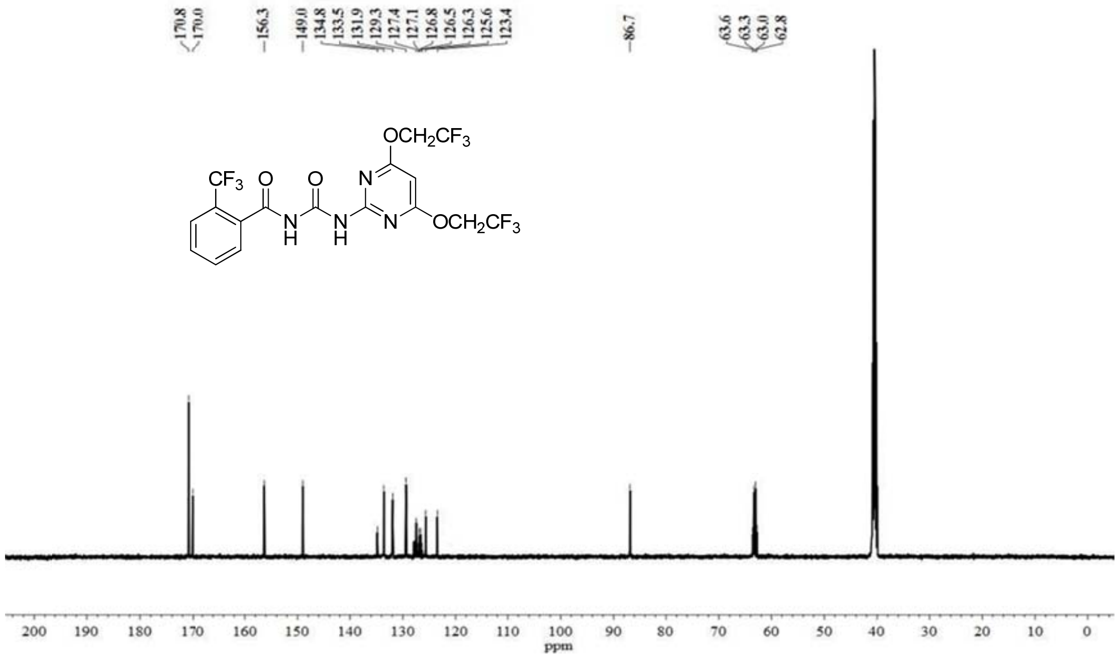

Figure S37 ^{13}C NMR spectra of compound 6

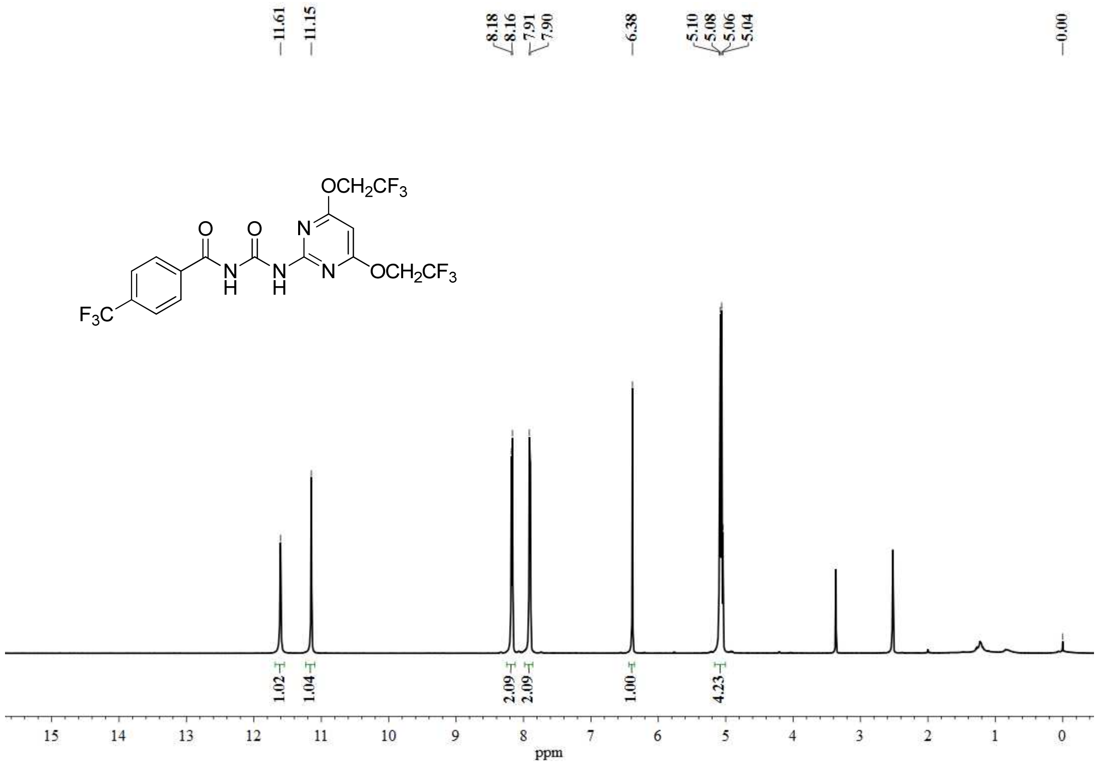

Figure S38 ^{1}H NMR spectra of compound 7

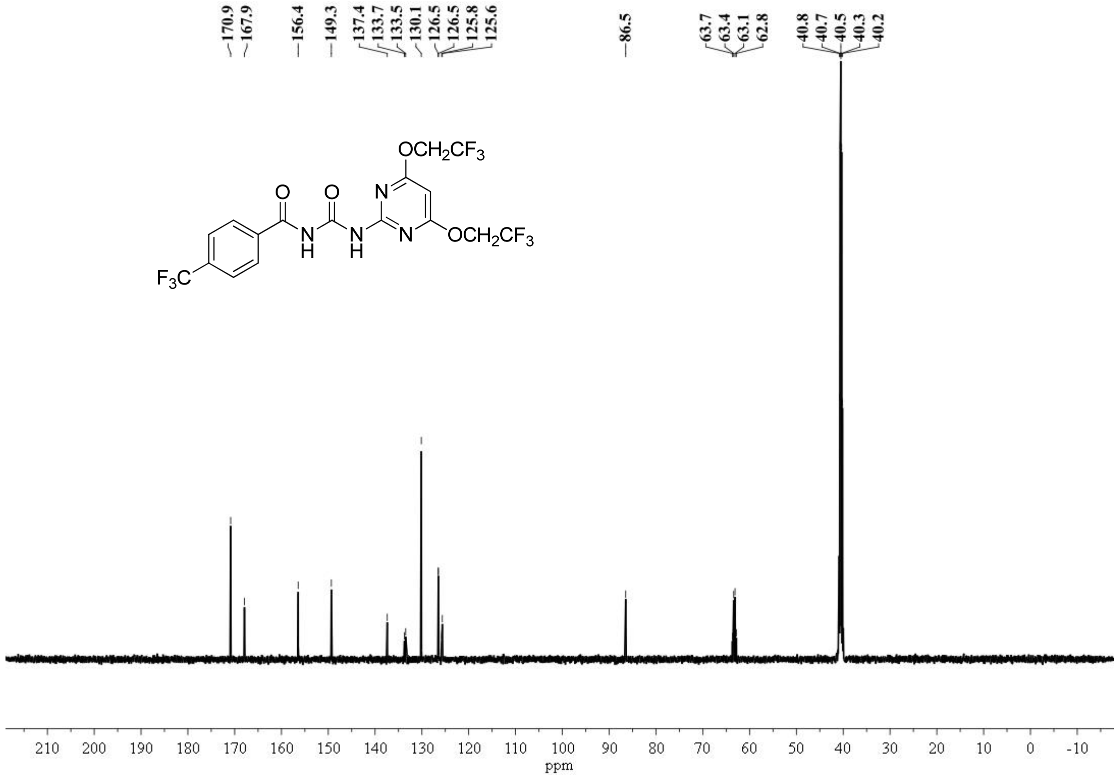

Figure S39 ^{13}C NMR spectra of compound 7

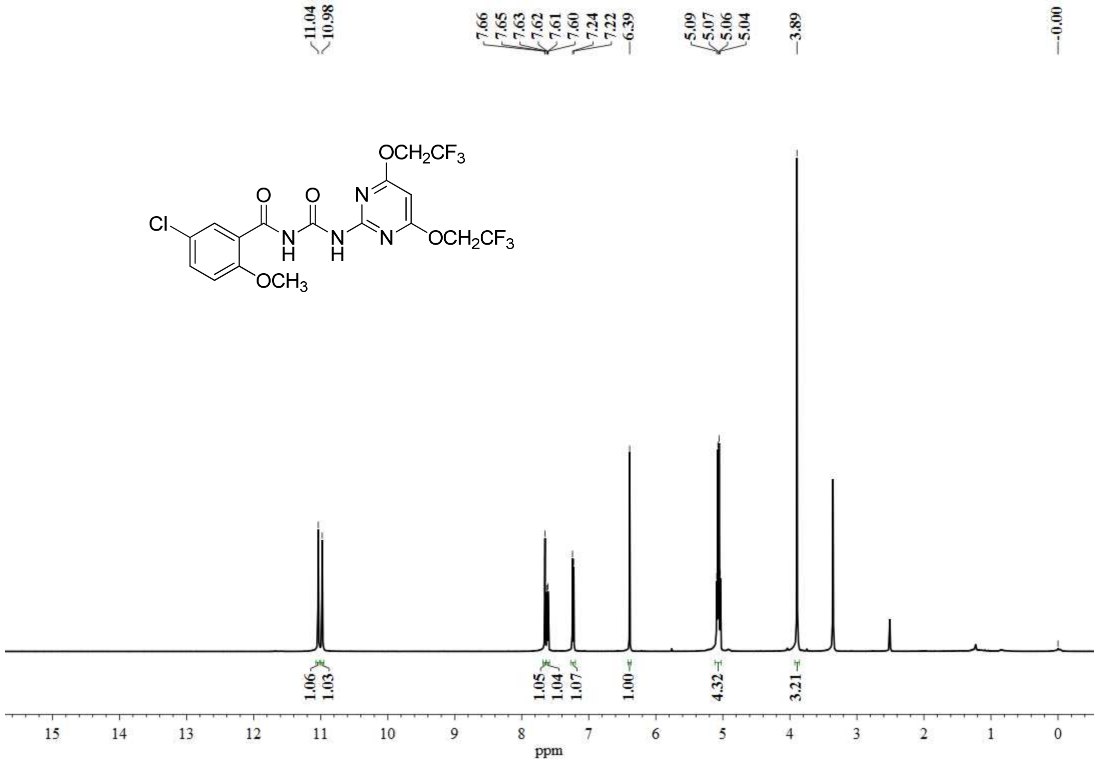

Figure S40 ^{1}H NMR spectra of compound 8

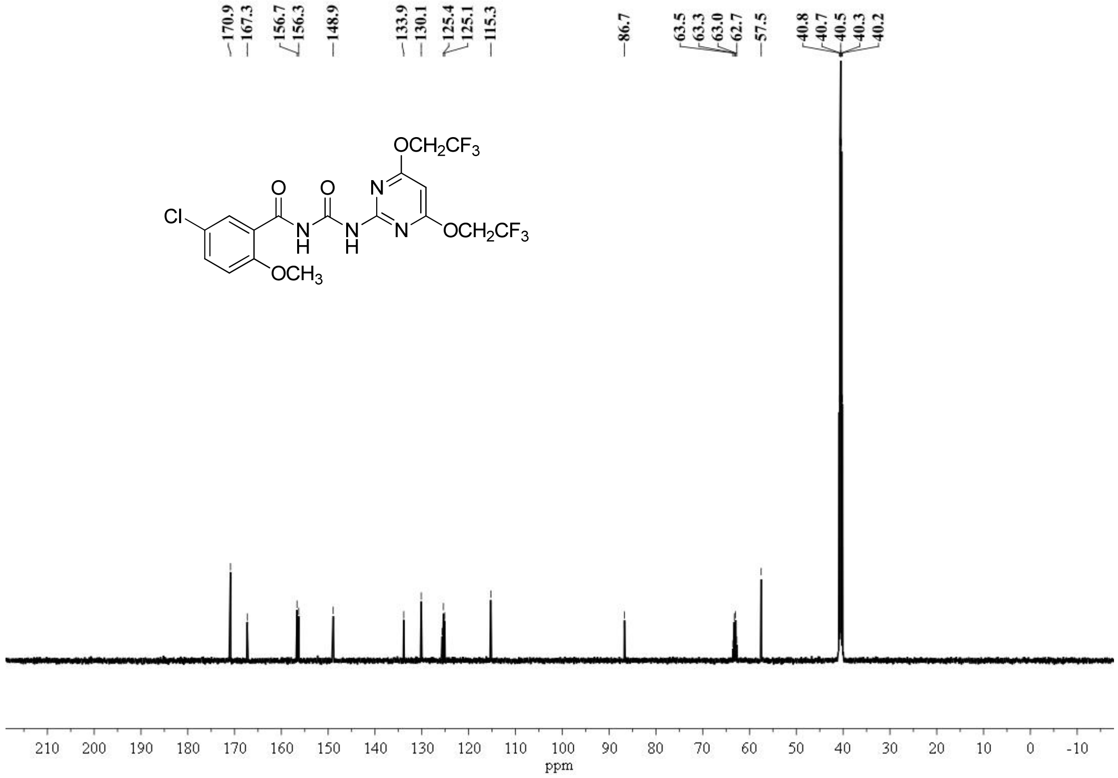

Figure S41 ^{13}C NMR spectra of compound 8

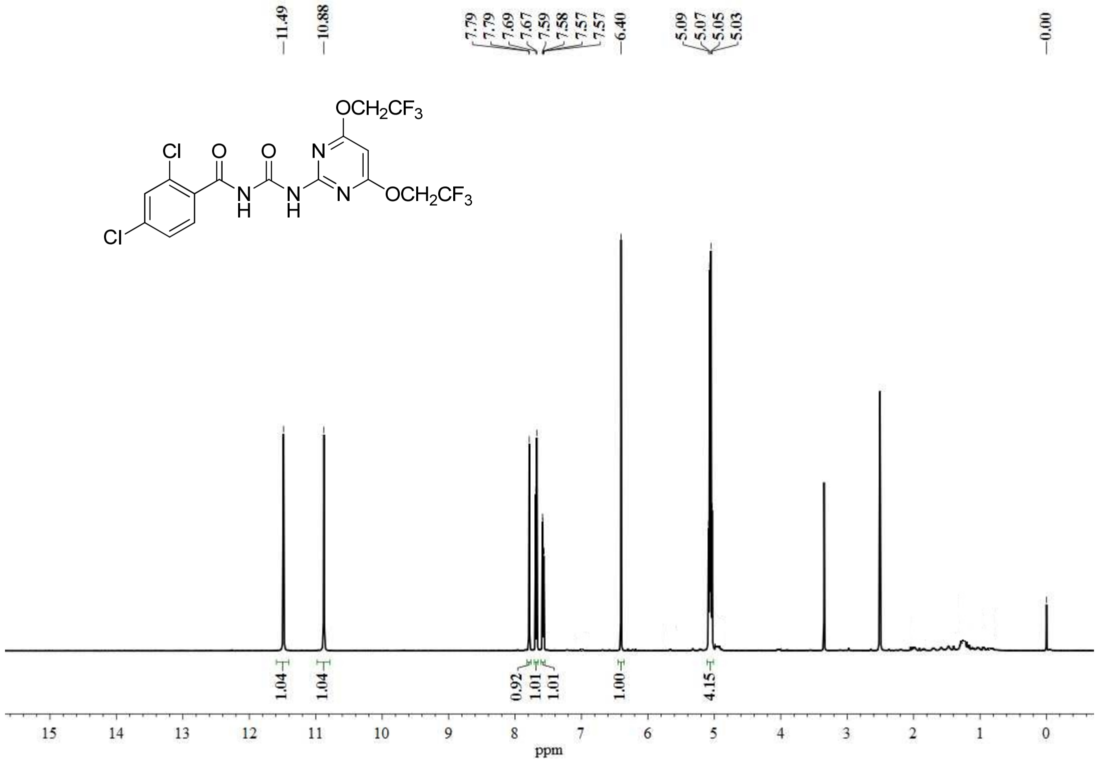

Figure S42 ^{1}H NMR spectra of compound 9

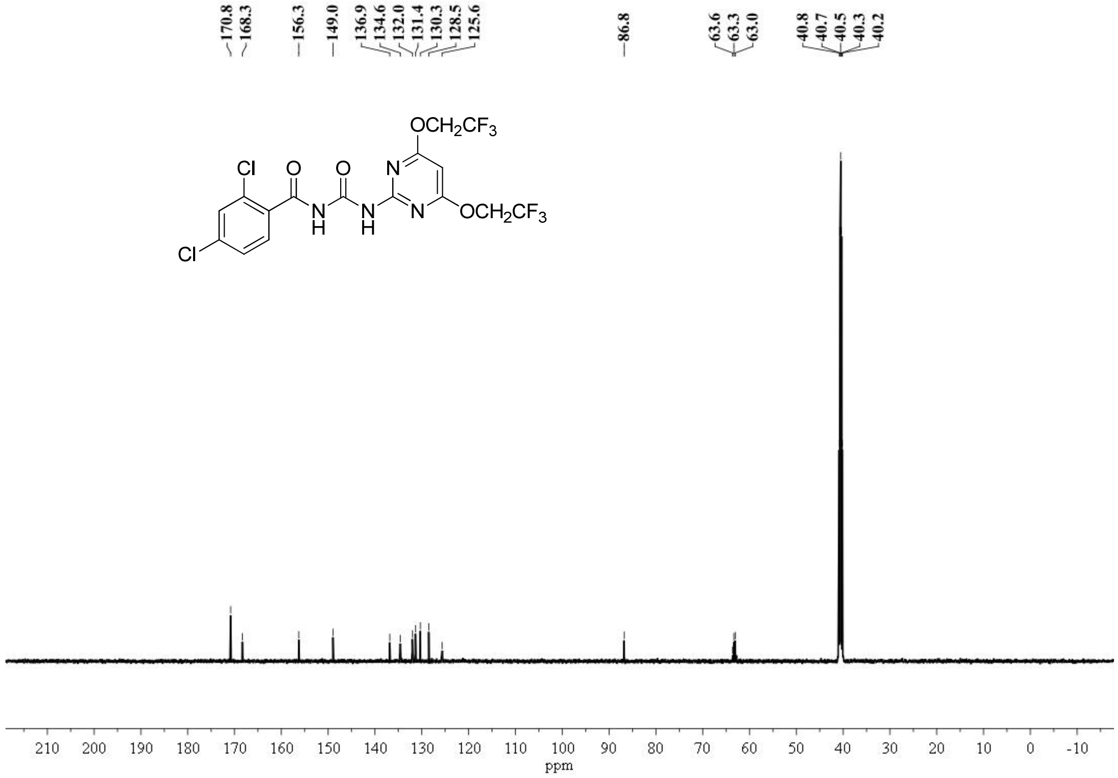

Figure S43 ^{13}C NMR spectra of compound 9

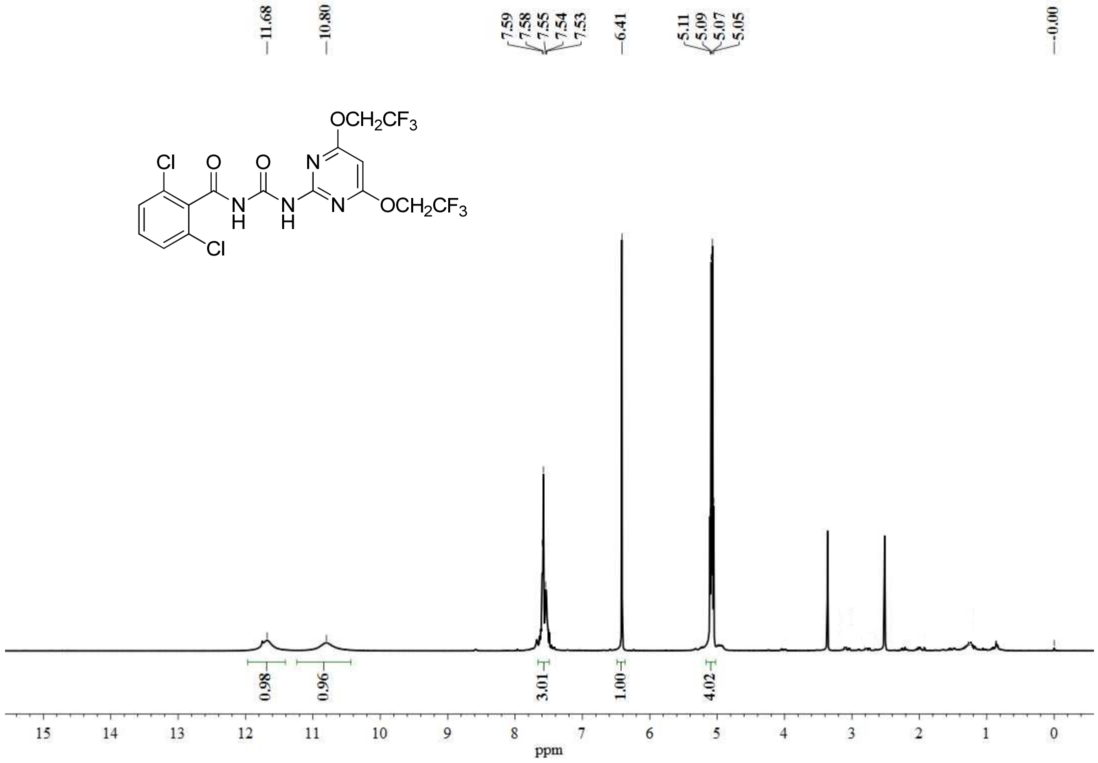

Figure S44 ^{1}H NMR spectra of compound 10

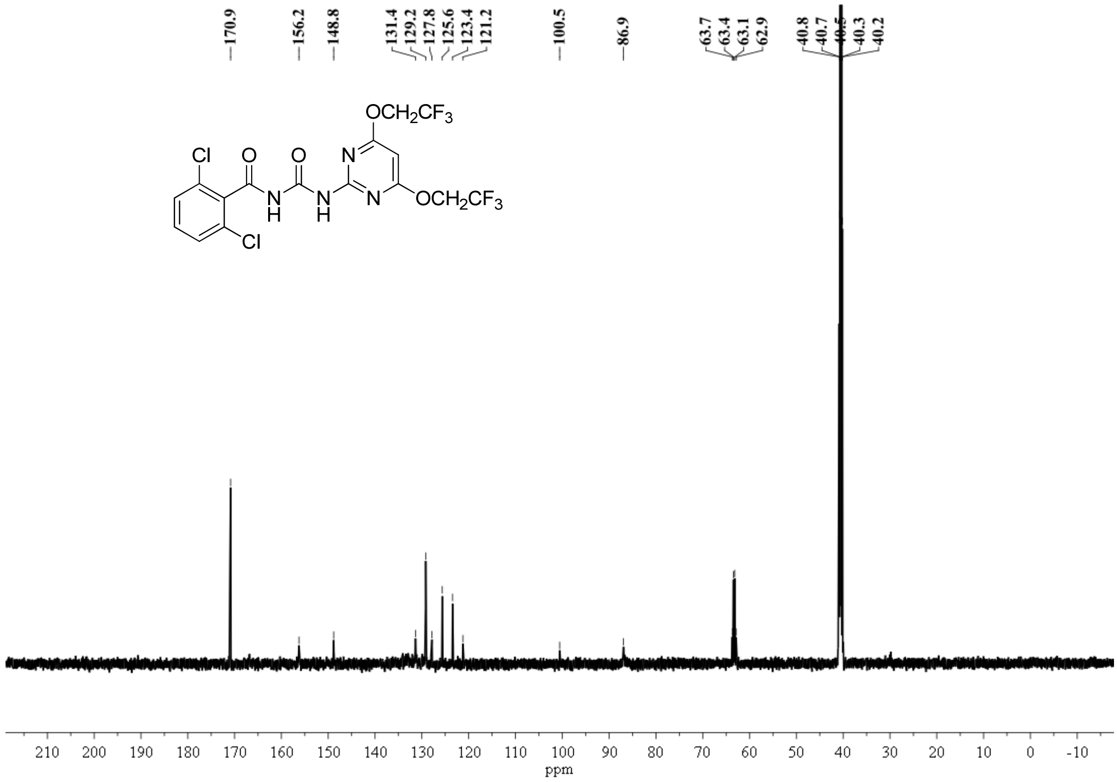

Figure S45 ^{13}C NMR spectra of compound 10

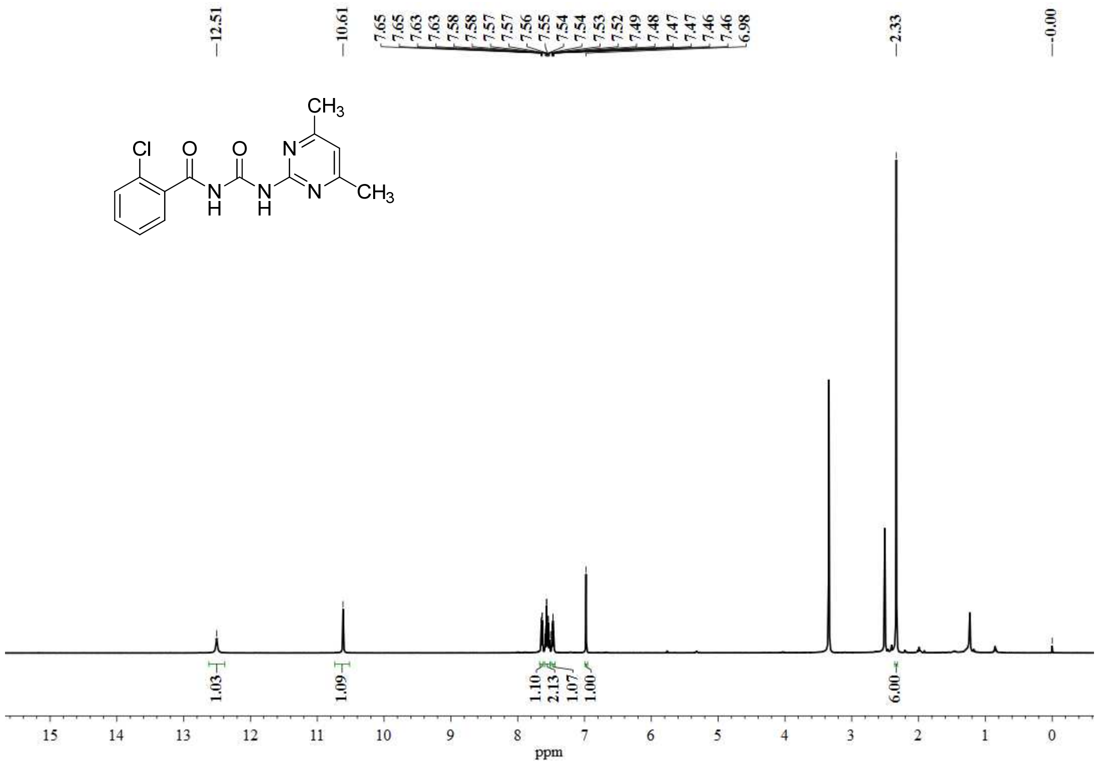

Figure S46 ^{1}H NMR spectra of compound 11

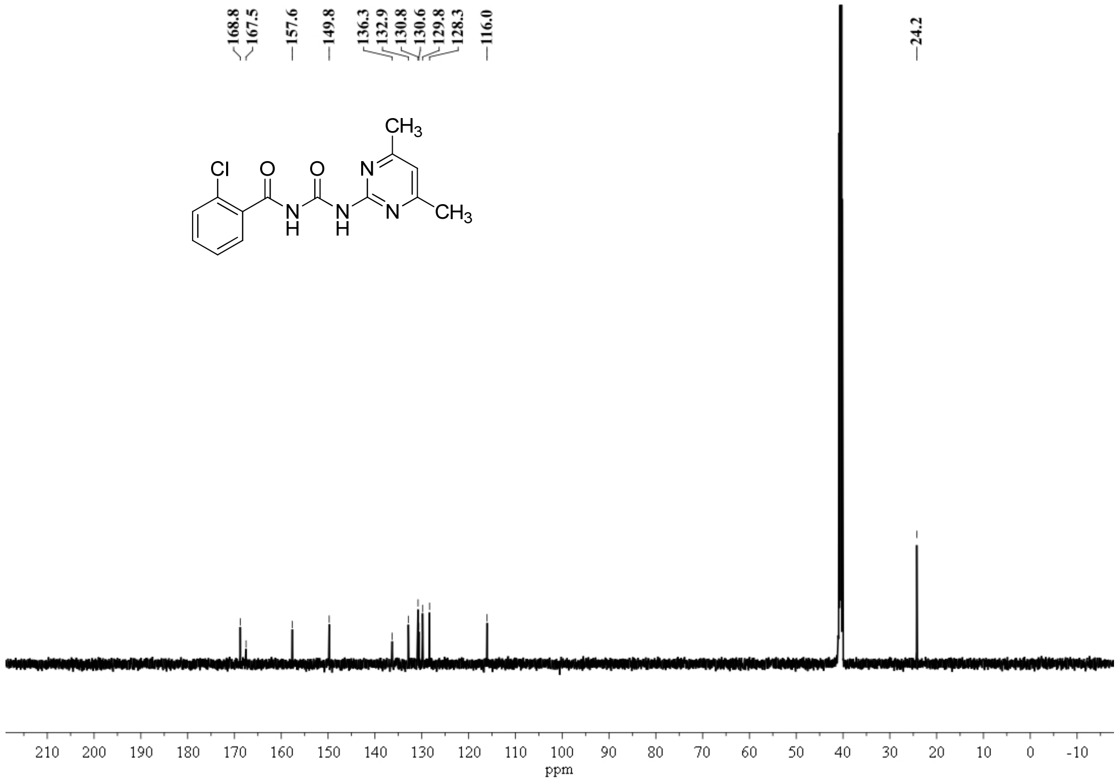

Figure S47 ^{13}C NMR spectra of compound 11

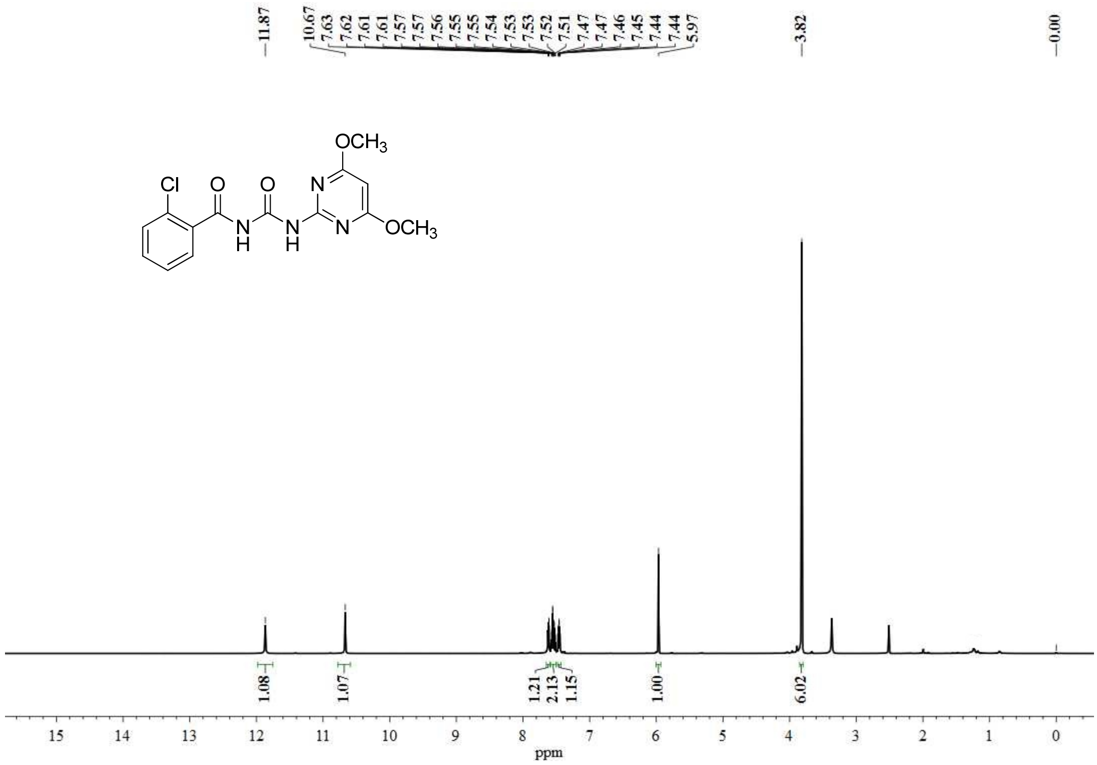

Figure S48 ^{1}H NMR spectra of compound 12

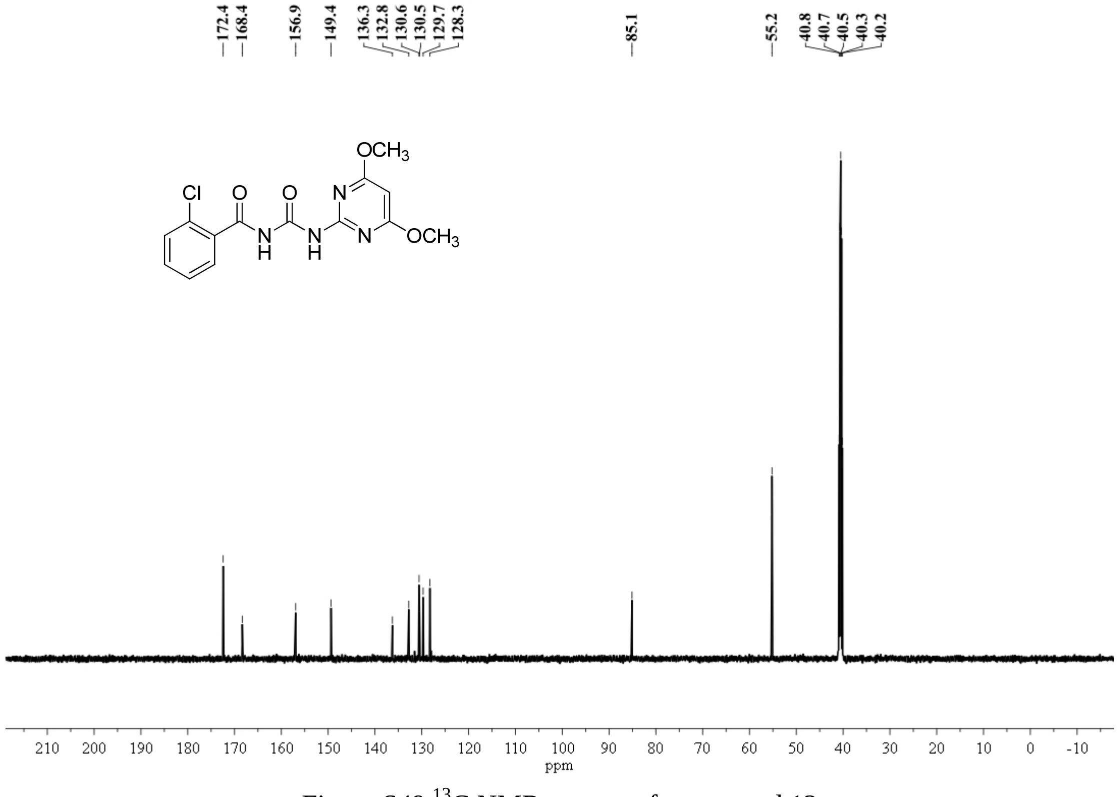

Figure S49 ^{13}C NMR spectra of compound 12

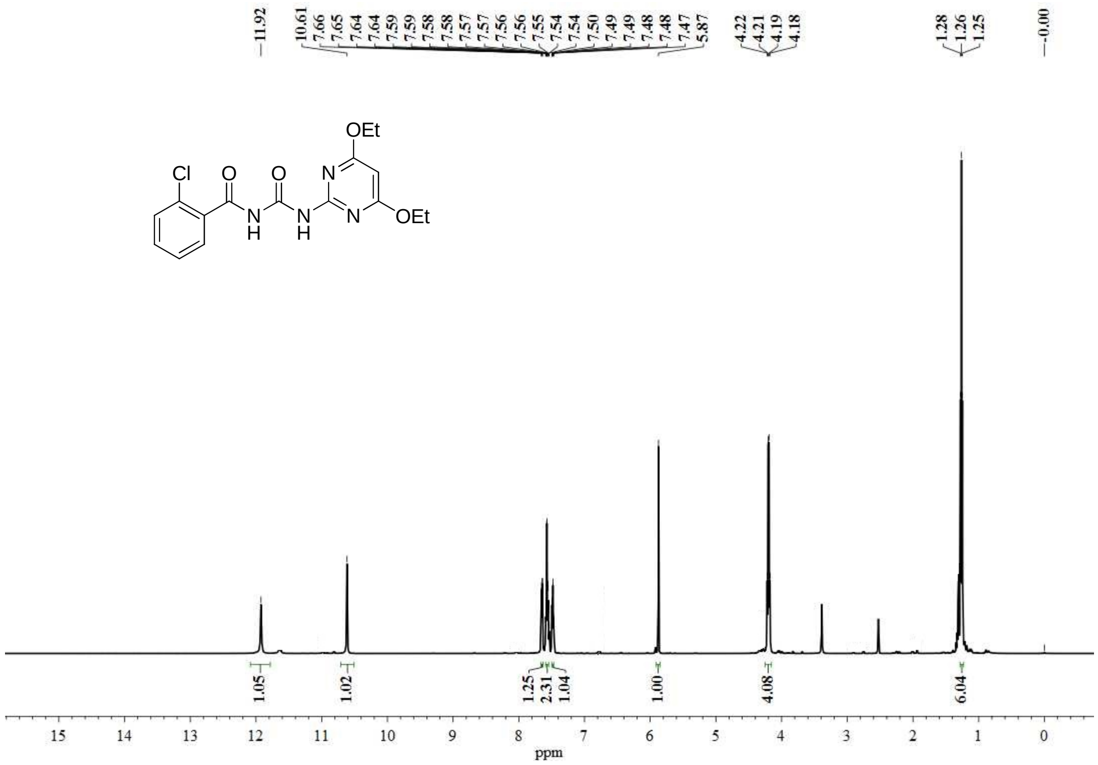

Figure S50 ^{1}H NMR spectra of compound 13

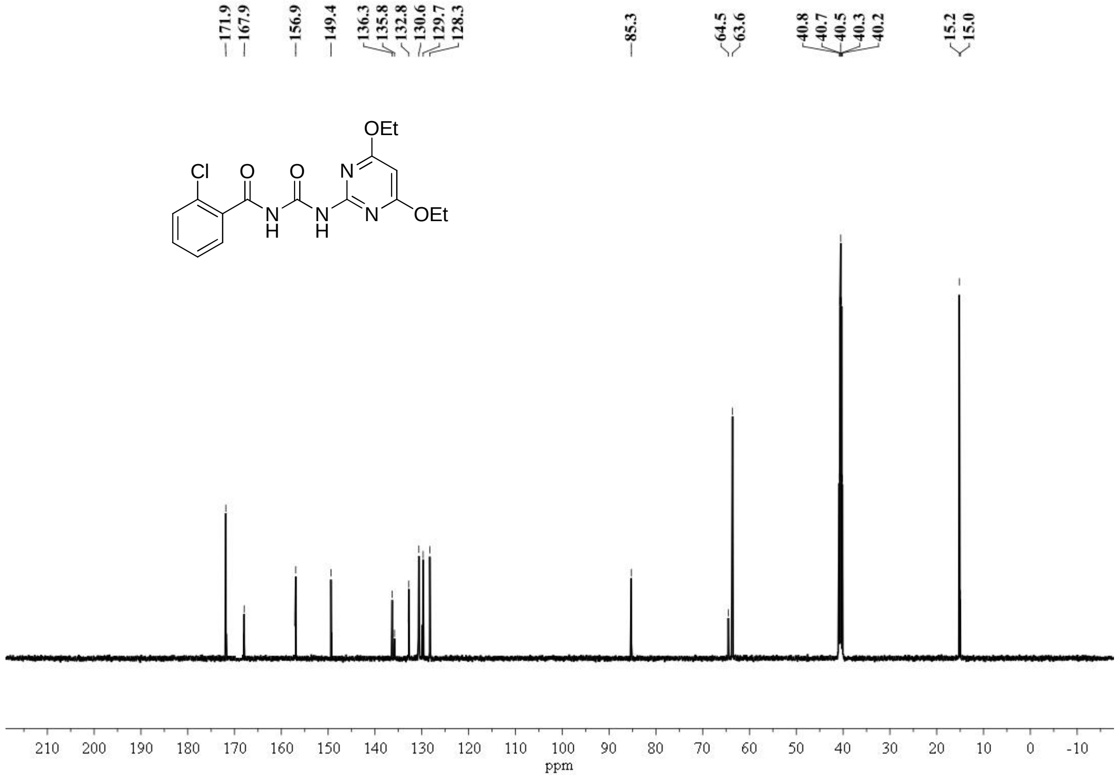

Figure S51 ^{13}C NMR spectra of compound 13

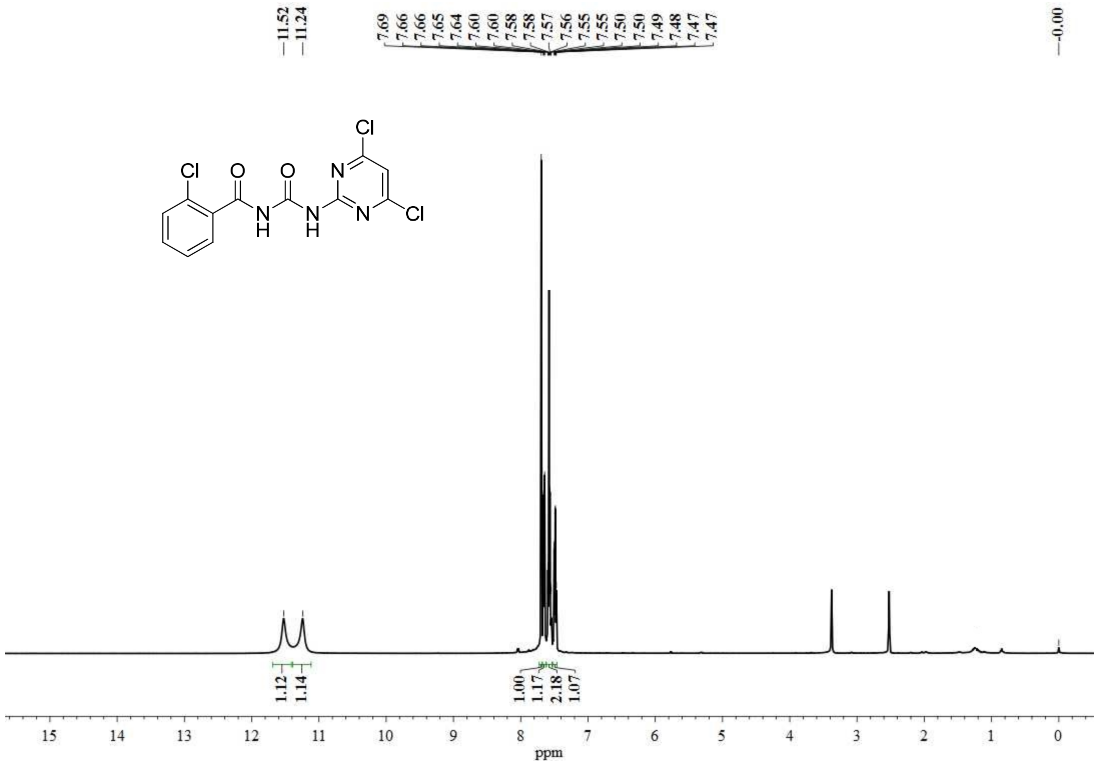

Figure S52 ^{1}H NMR spectra of compound 14

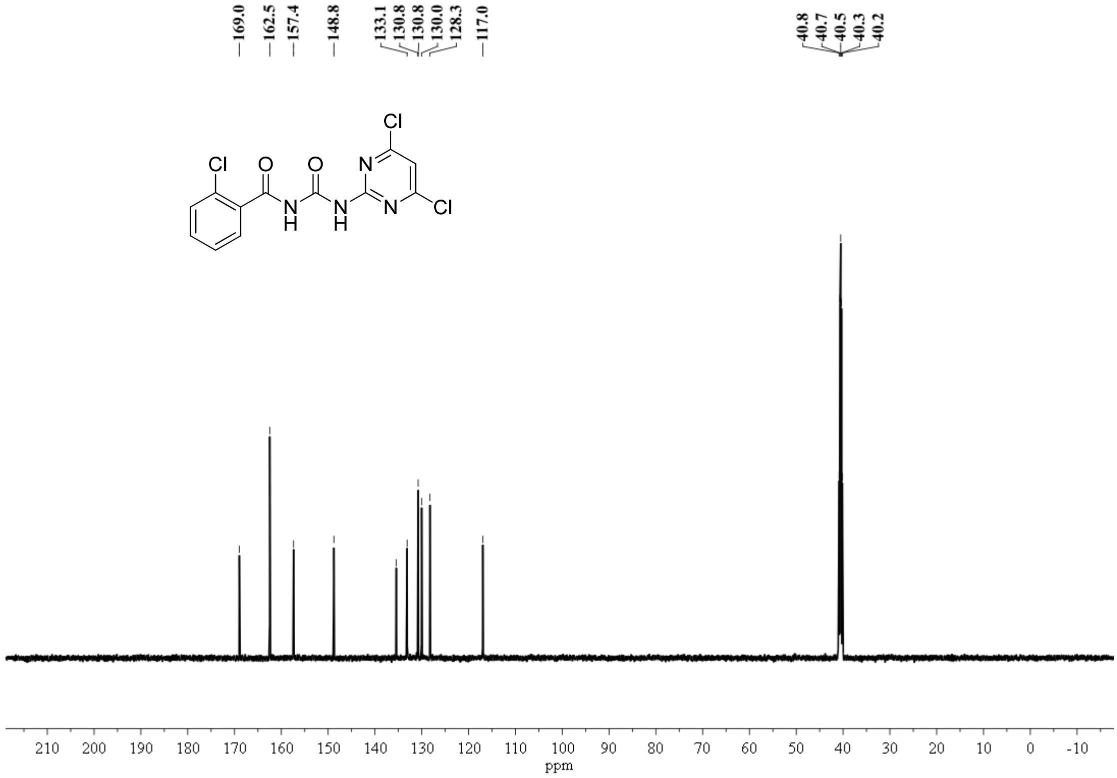

Figure S53 ^{13}C NMR spectra of compound 14

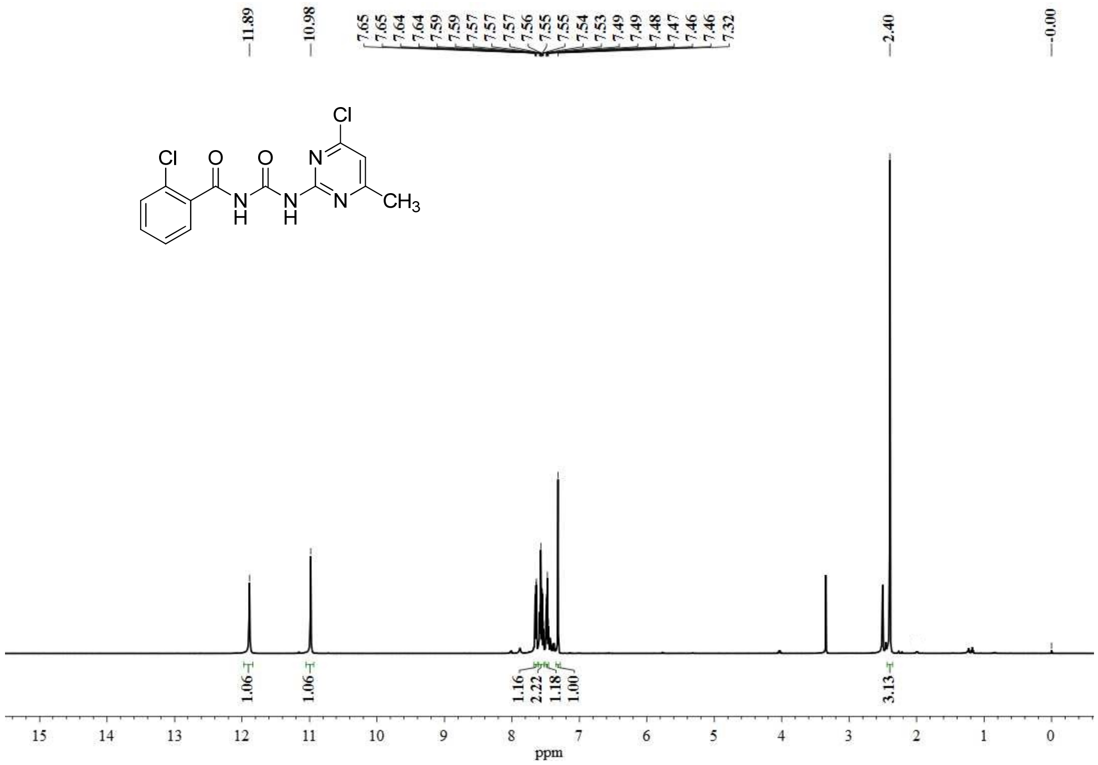

Figure S54 ^{1}H NMR spectra of compound 15

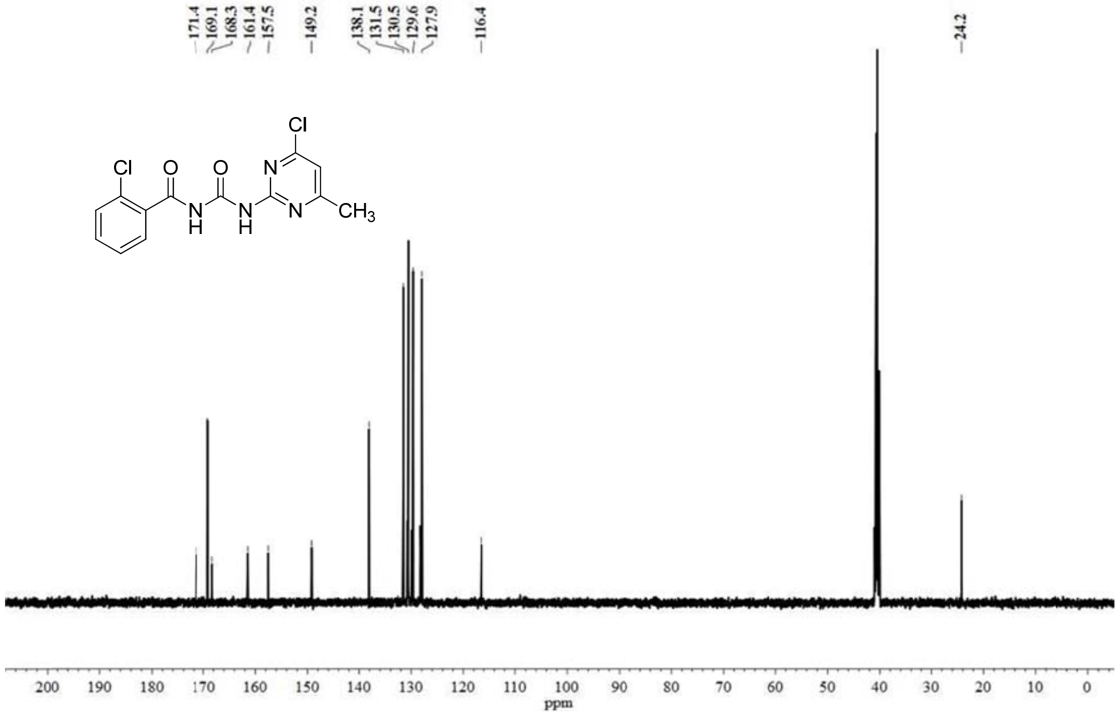

Figure S55 ^{13}C NMR spectra of compound 15

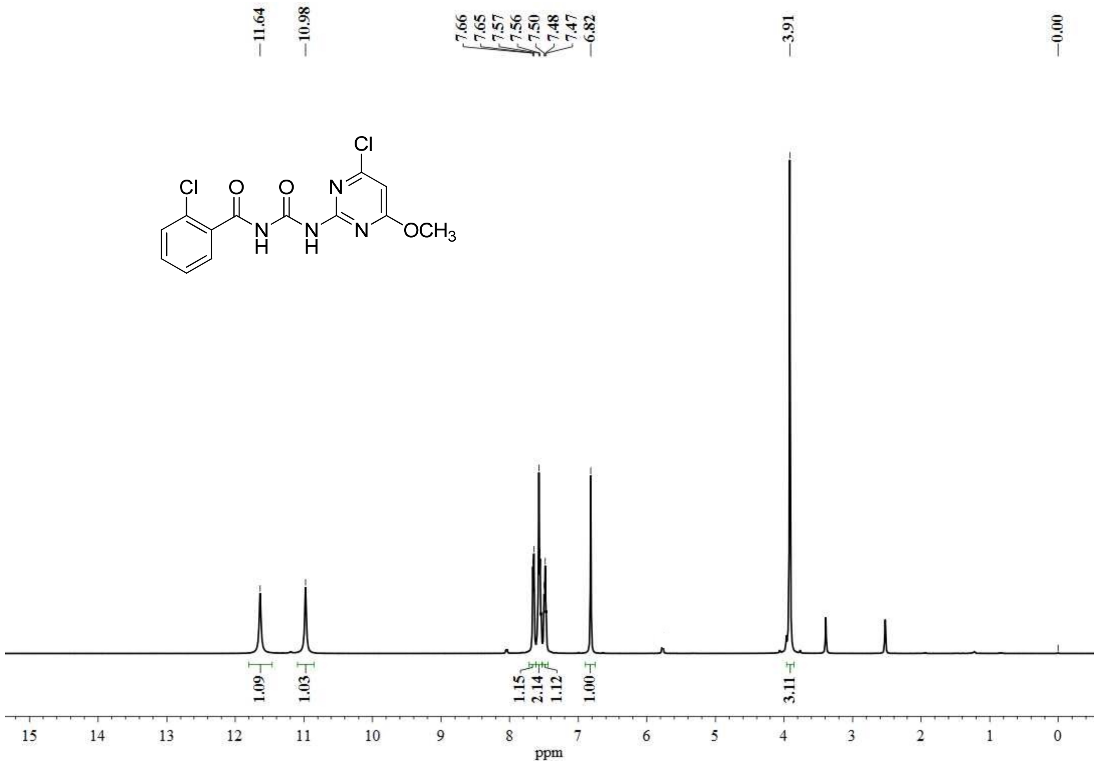

Figure S56 ^{1}H NMR spectra of compound 16

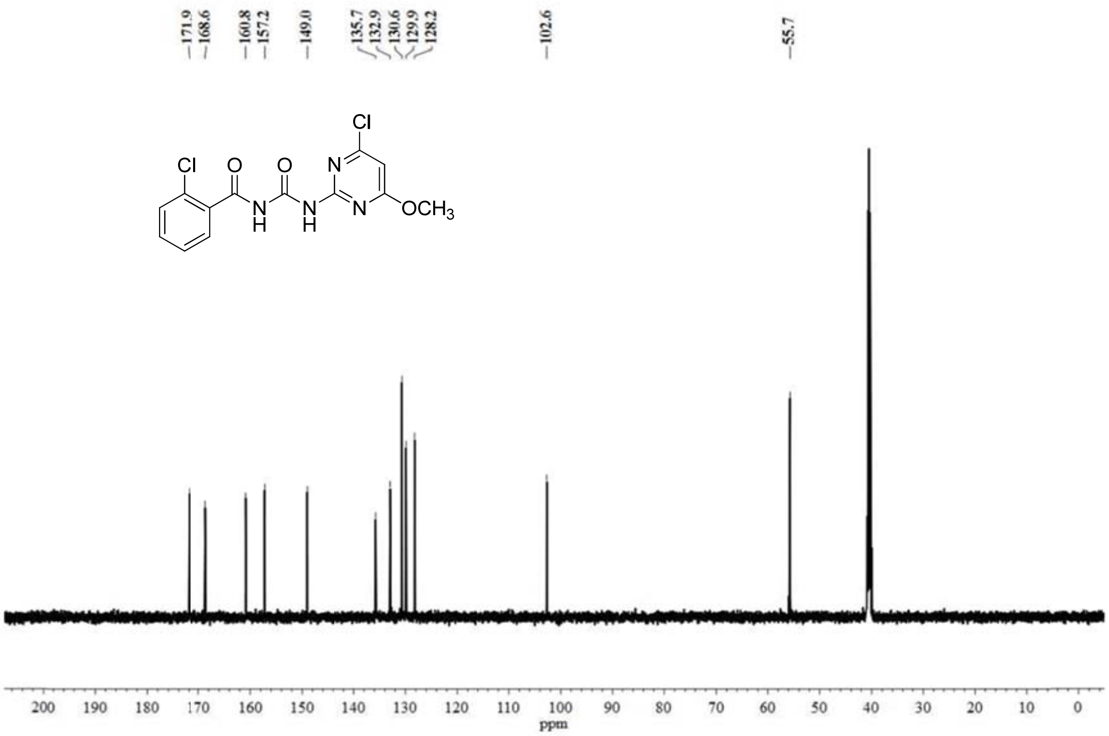

Figure S57 ^{13}C NMR spectra of compound 16

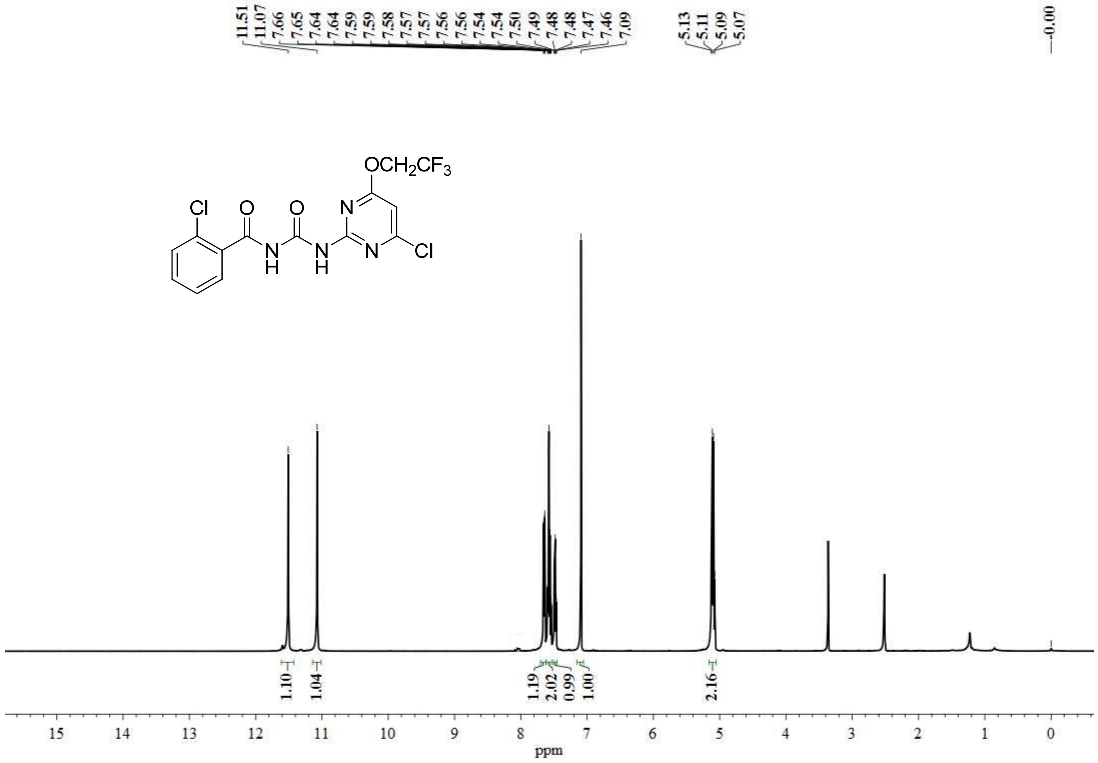

Figure S58 ^{1}H NMR spectra of compound 17

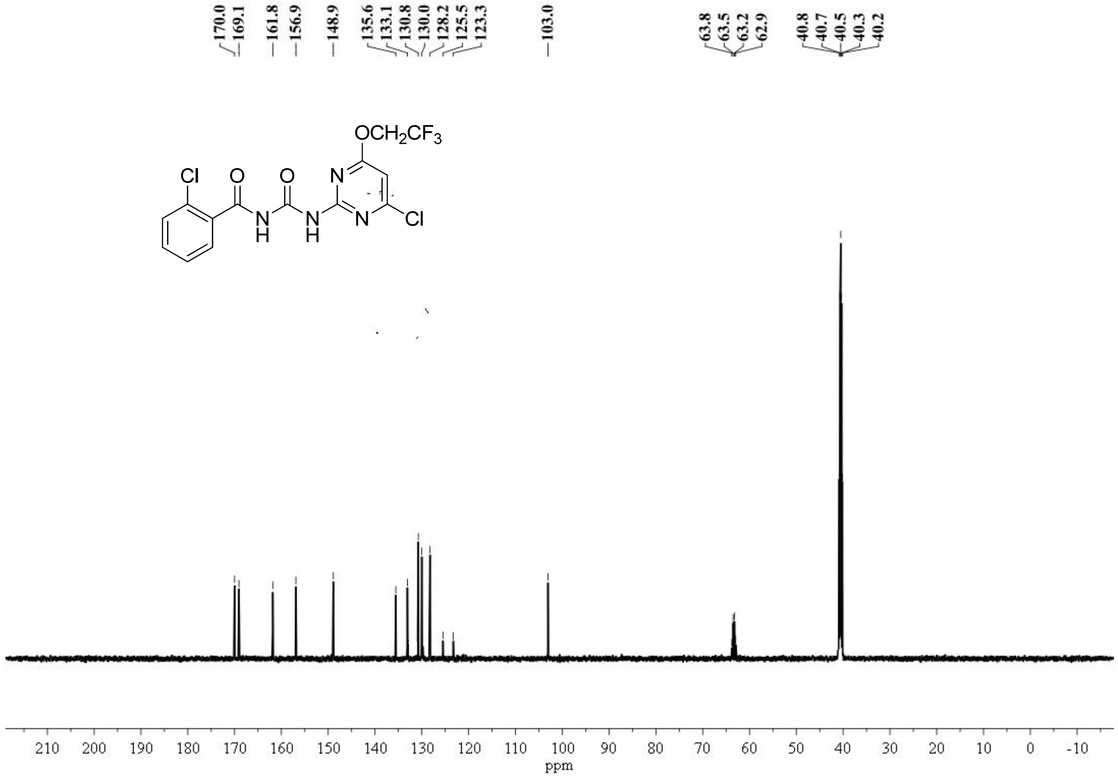

Figure S59 ^{13}C NMR spectra of compound 17

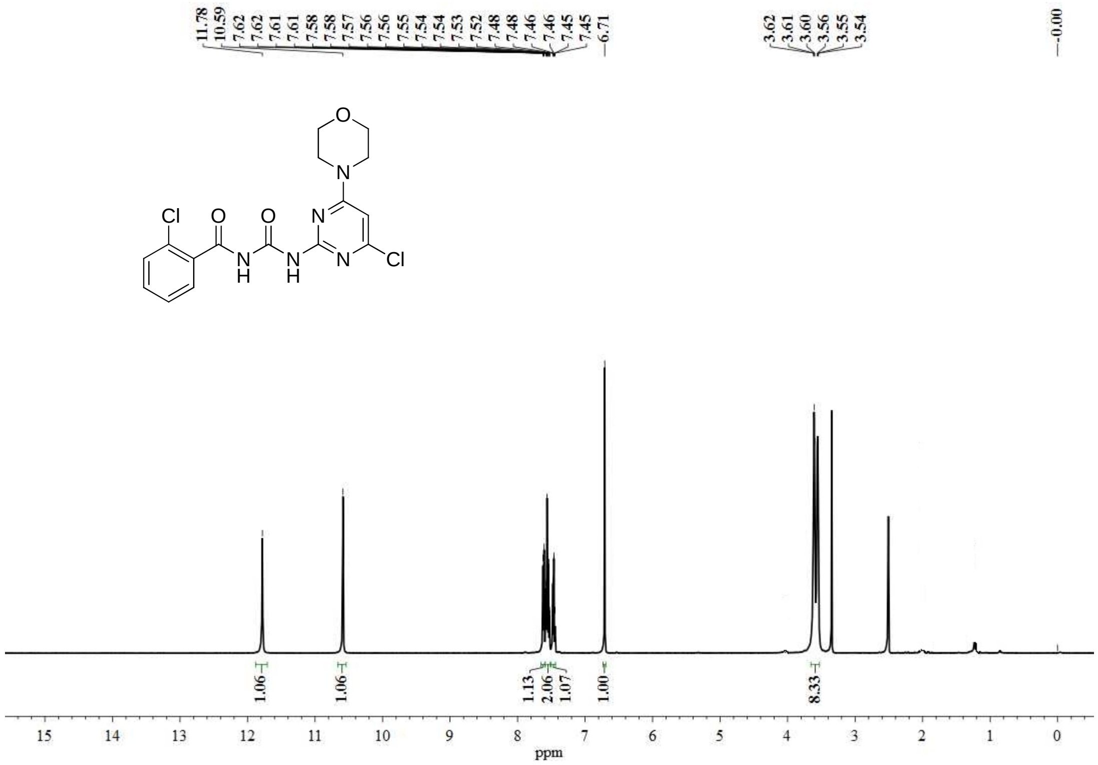

Figure S60 ^{1}H NMR spectra of compound 18

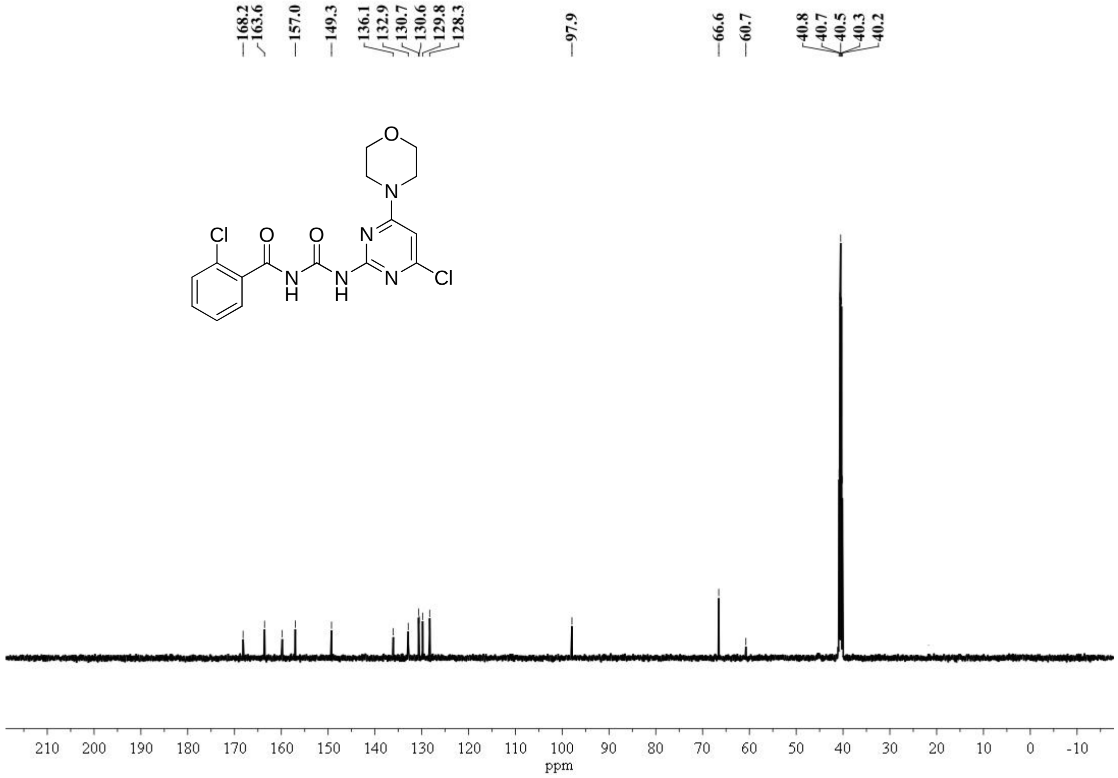

Figure S61 ^{13}C NMR spectra of compound 18

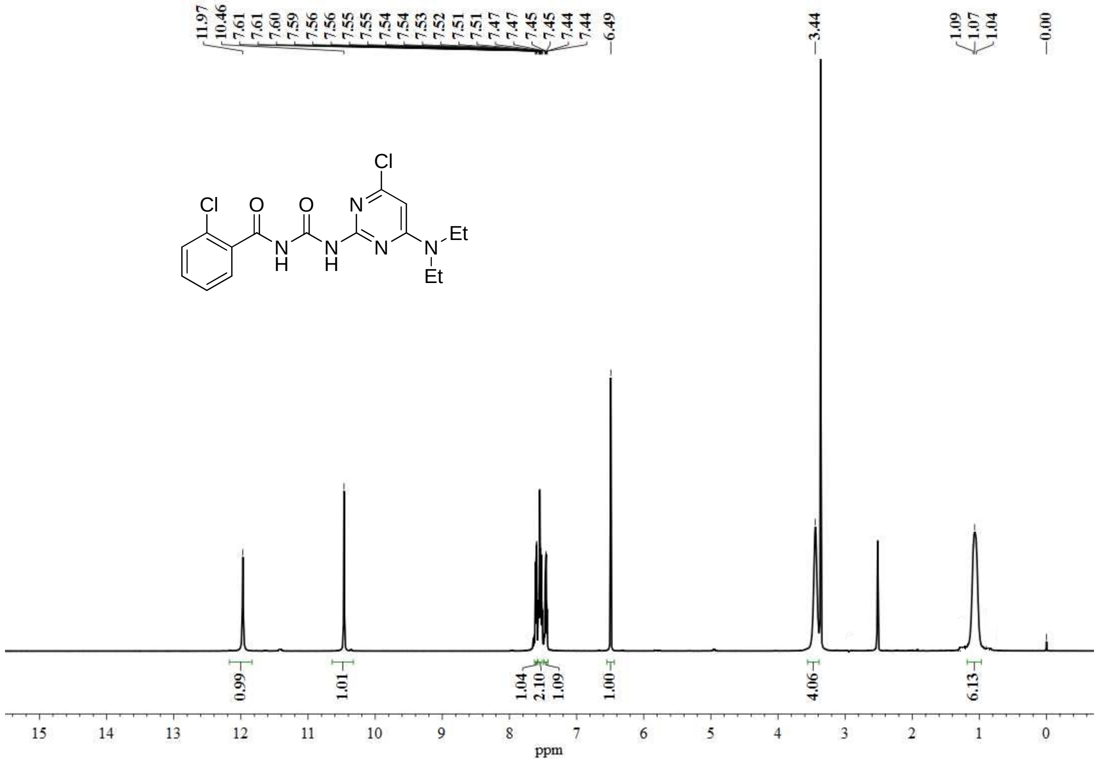

Figure S62 ^{1}H NMR spectra of compound 19

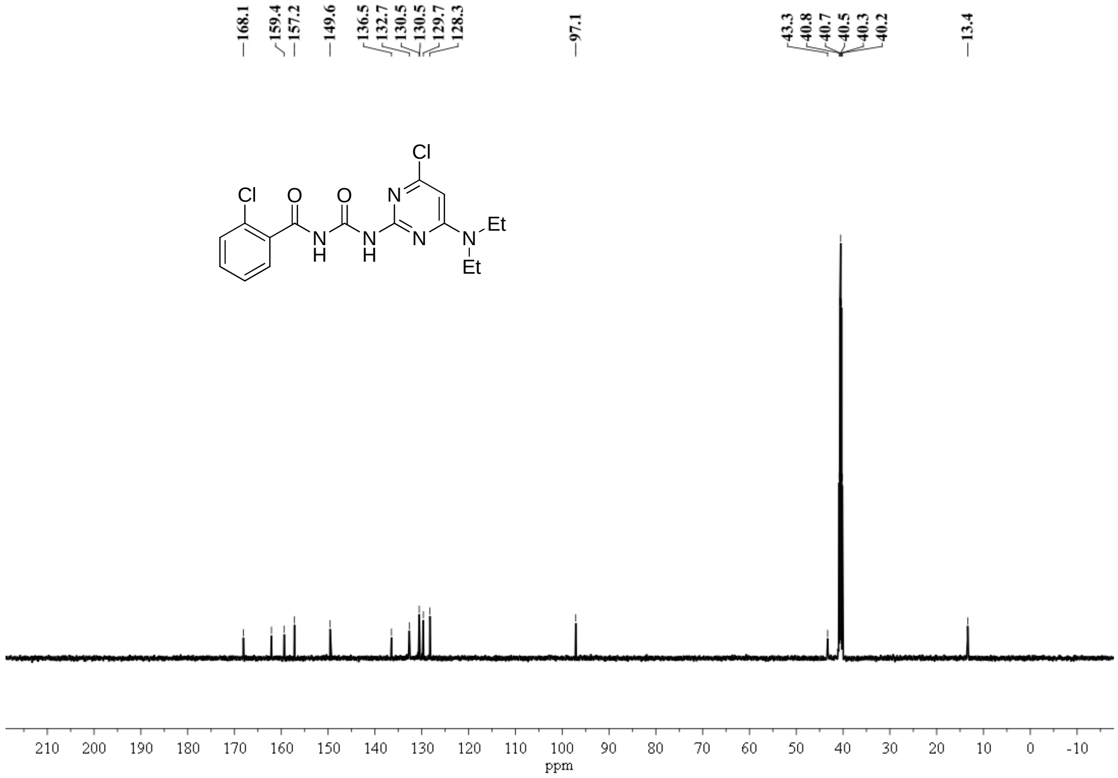

Figure S63 ^{13}C NMR spectra of compound 19

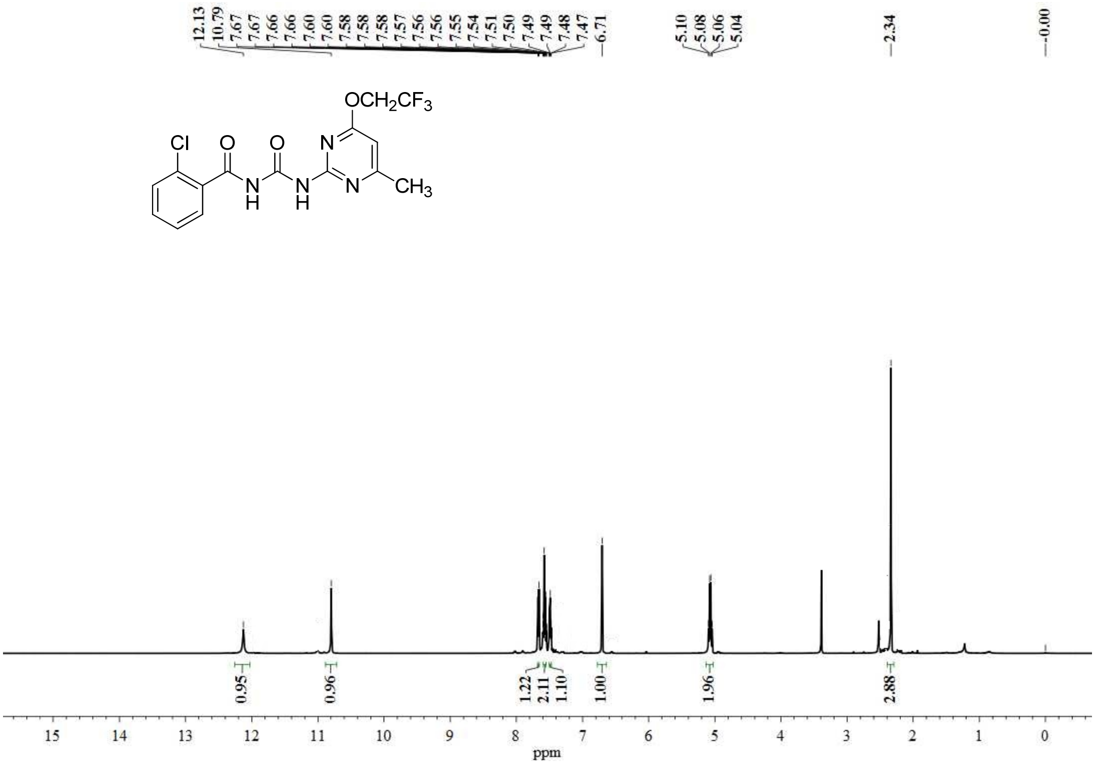

Figure S64 ^{1}H NMR spectra of compound 20

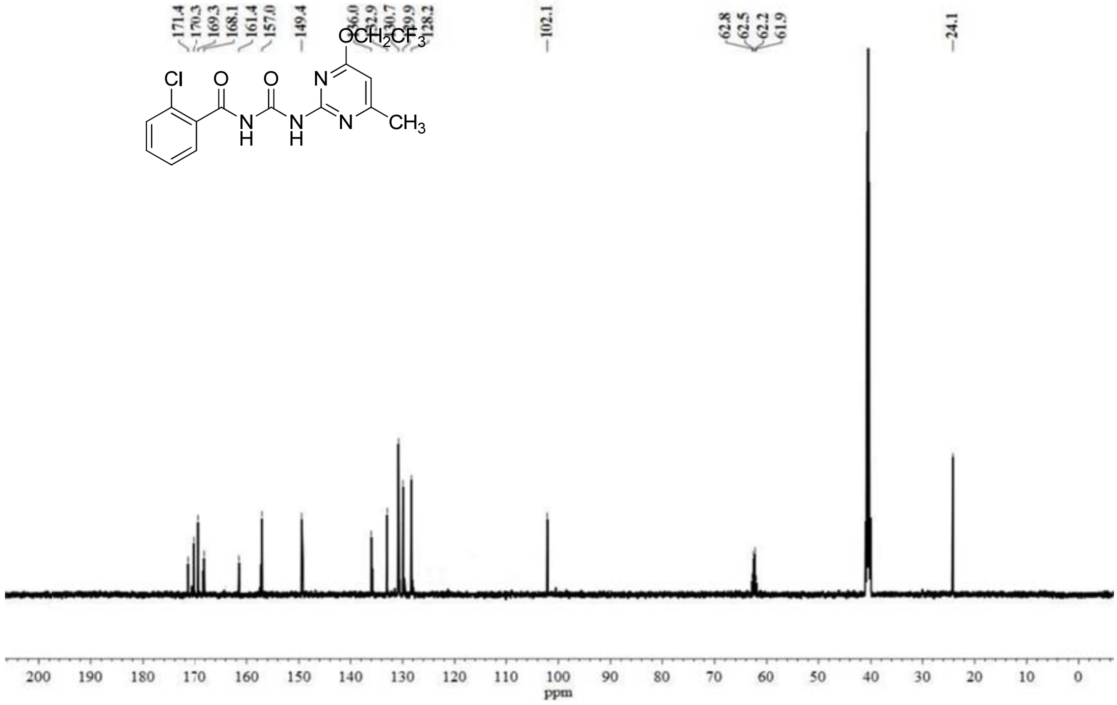

Figure S65 ^{13}C NMR spectra of compound 20

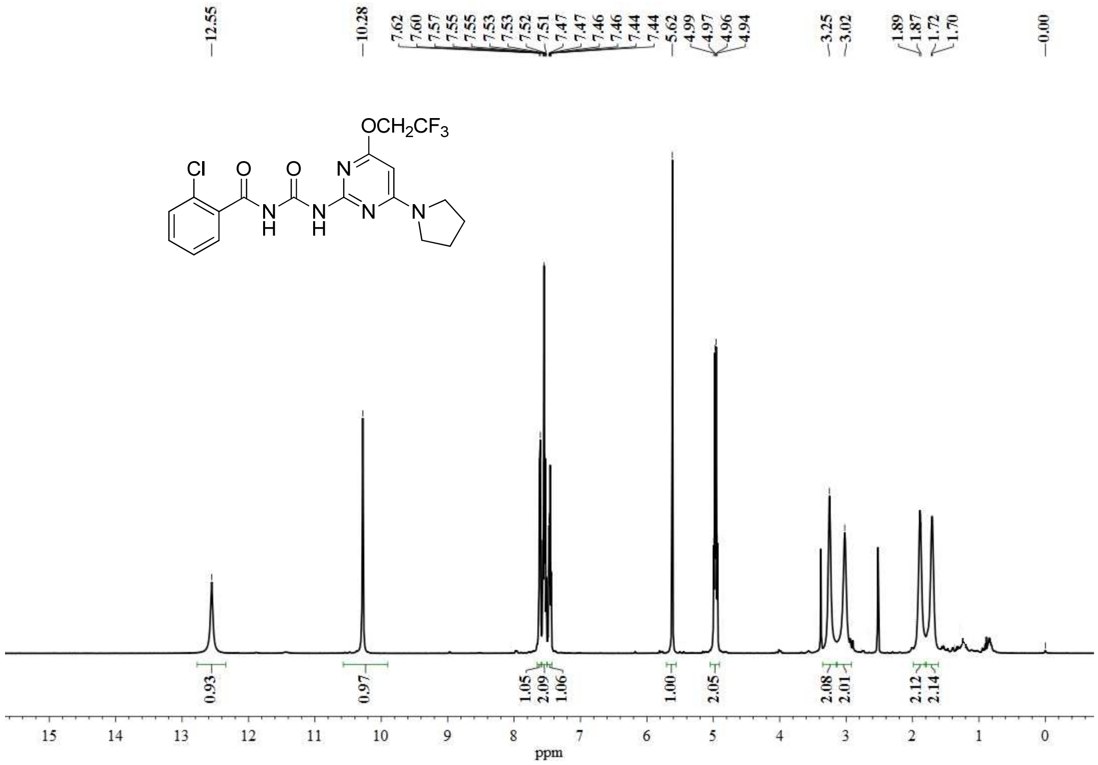

Figure S66 ^{1}H NMR spectra of compound 21

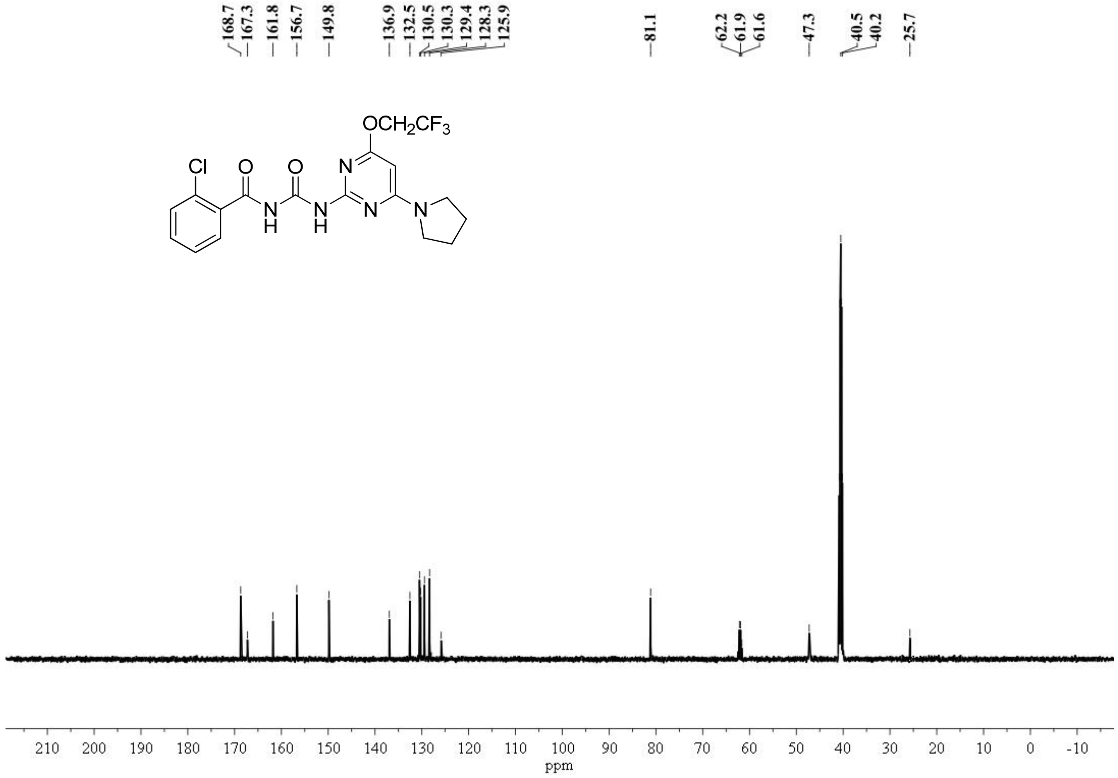

Figure S67 ^{13}C NMR spectra of compound 21

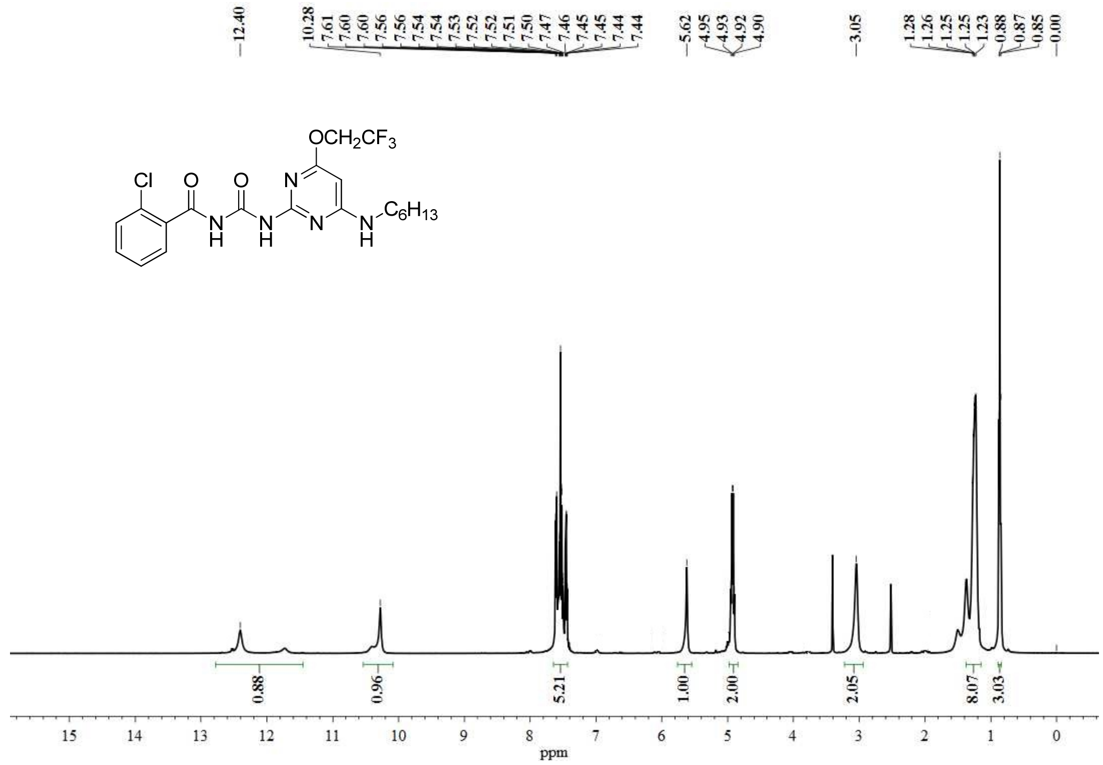

Figure S68 ^{1}H NMR spectra of compound 22

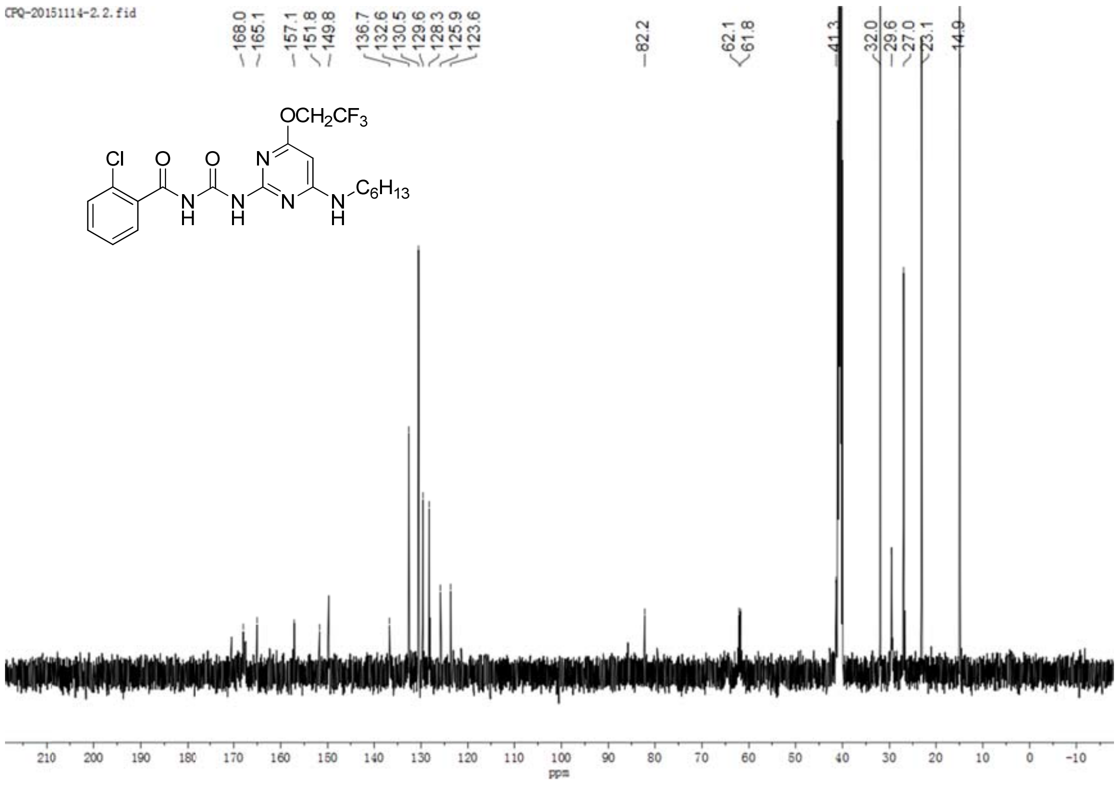

Figure S69 ^{13}C NMR spectra of compound 22

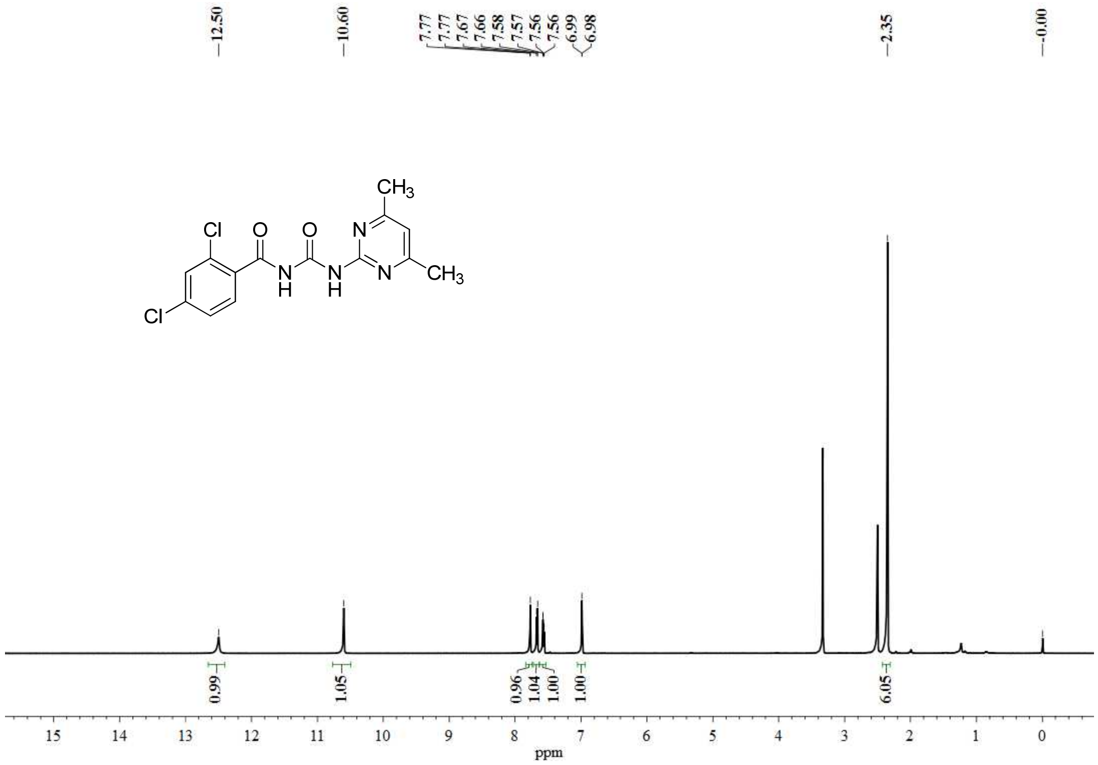

Figure S70 ^{1}H NMR spectra of compound 23

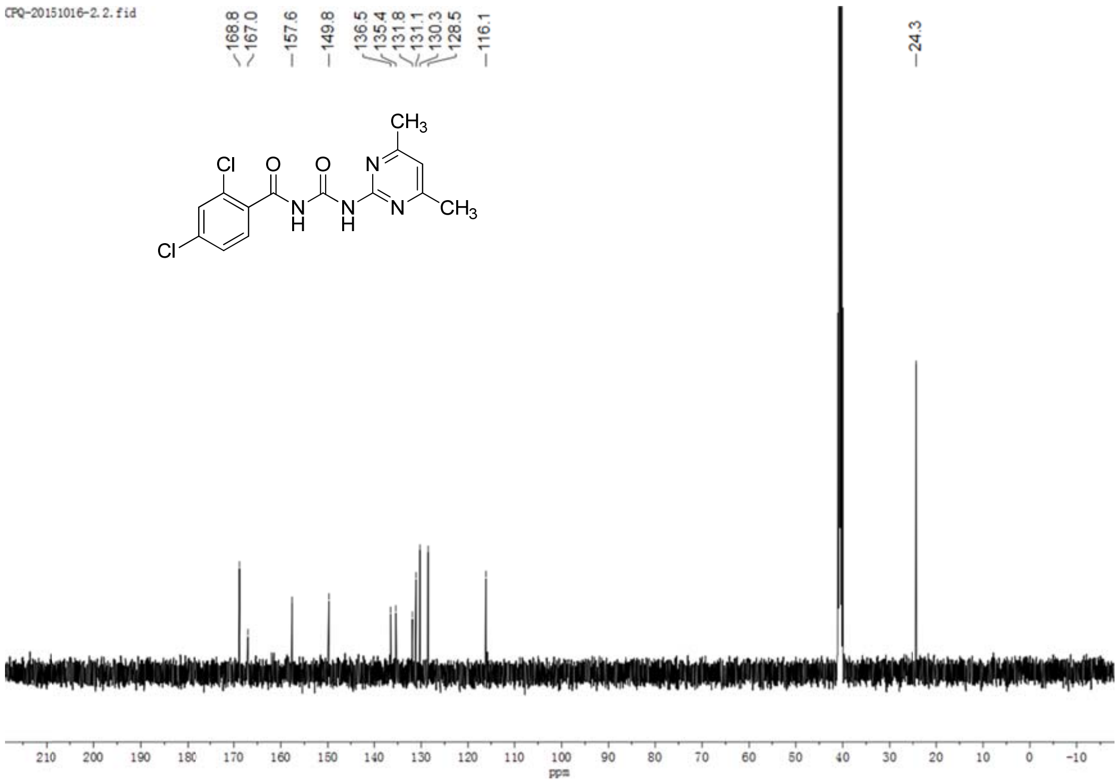

Figure S71 ^{13}C NMR spectra of compound 23

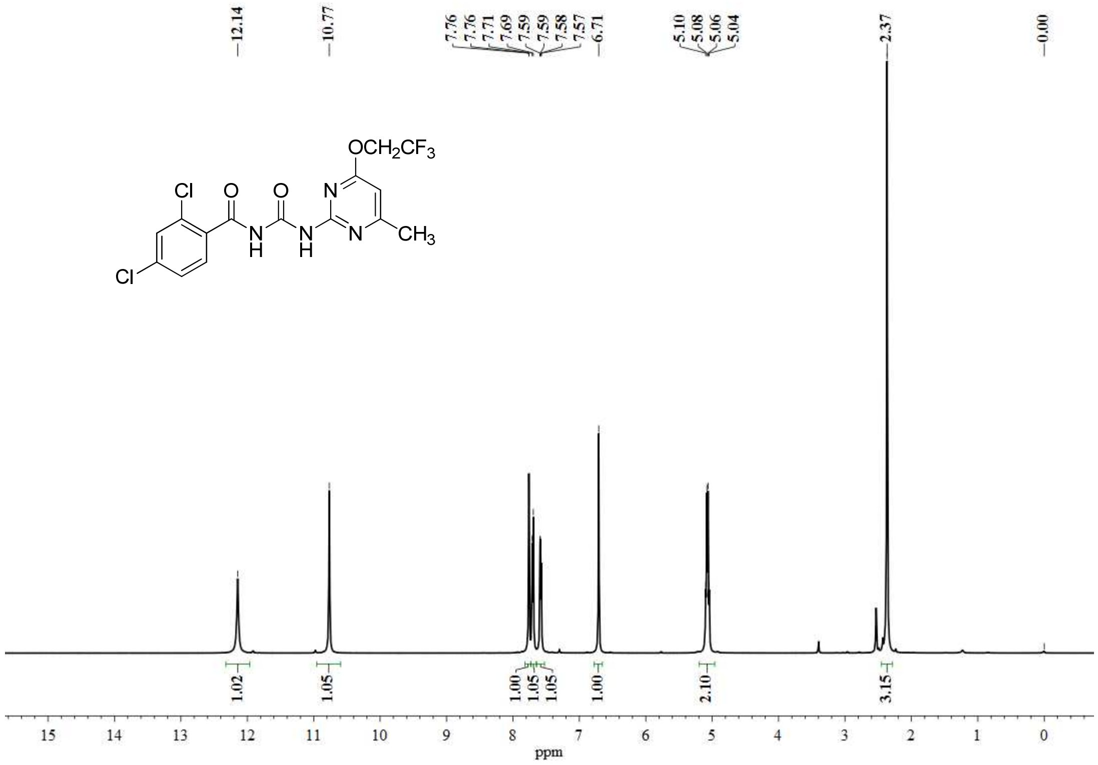

Figure S72 ^{1}H NMR spectra of compound 24

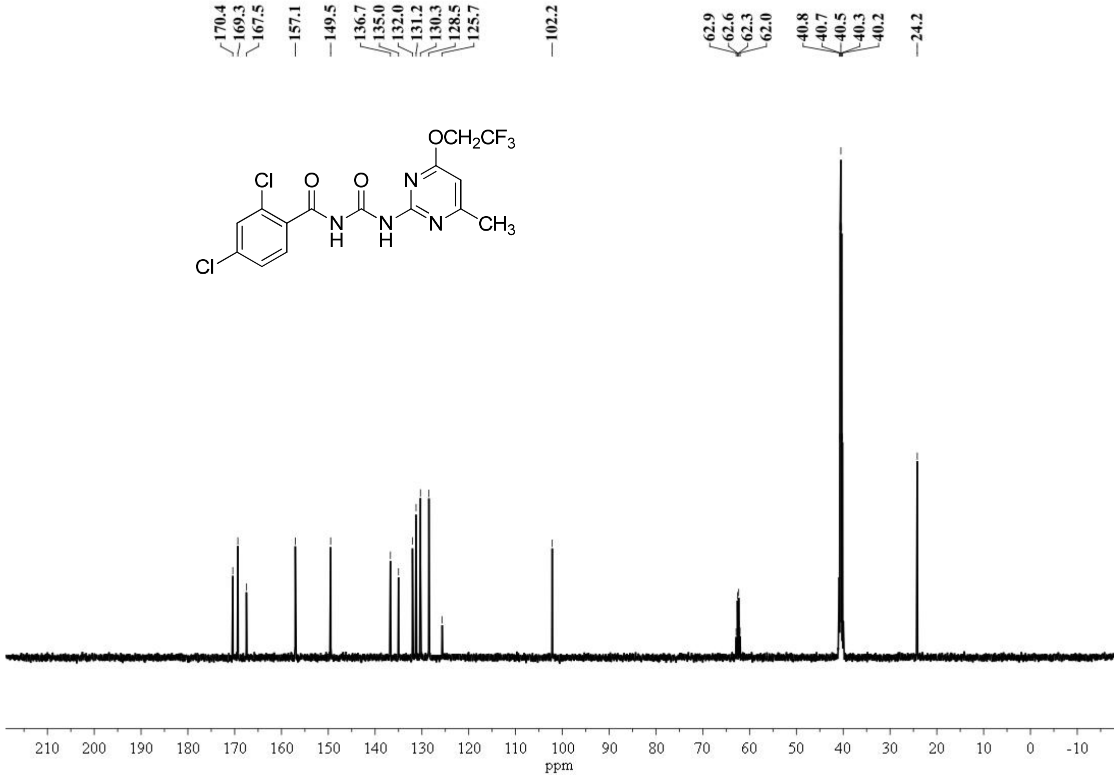

Figure S73 ^{13}C NMR spectra of compound 24

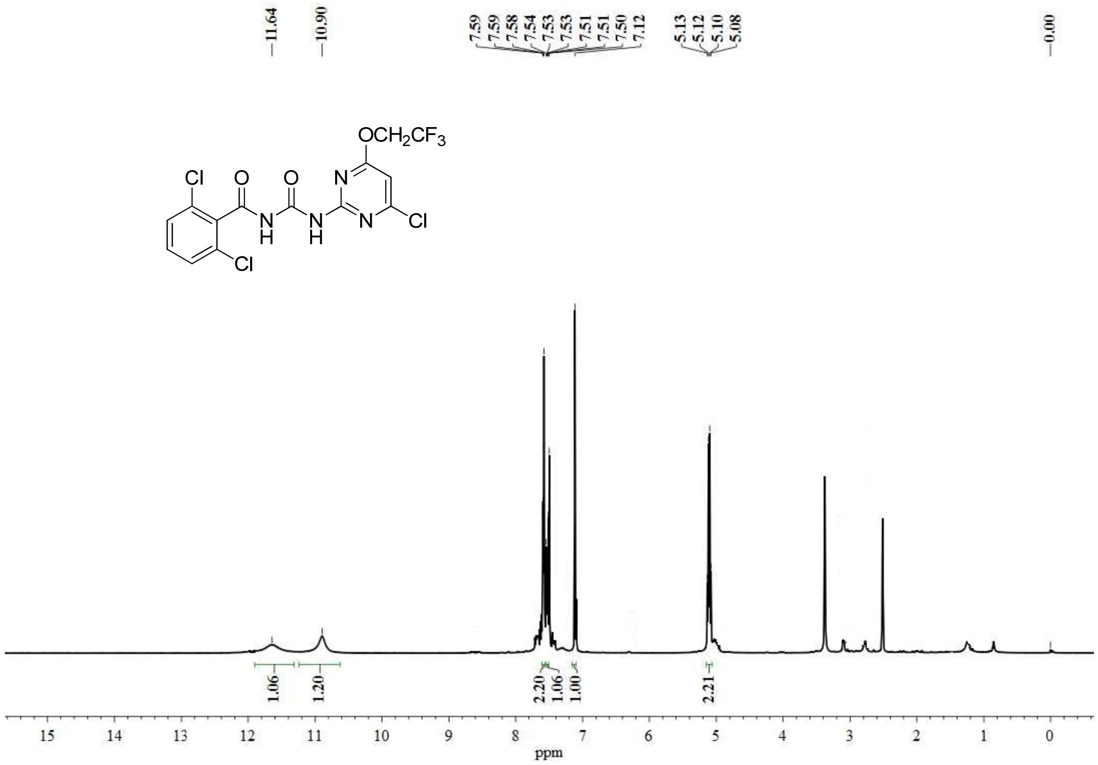

Figure S74 ^{1}H NMR spectra of compound 25

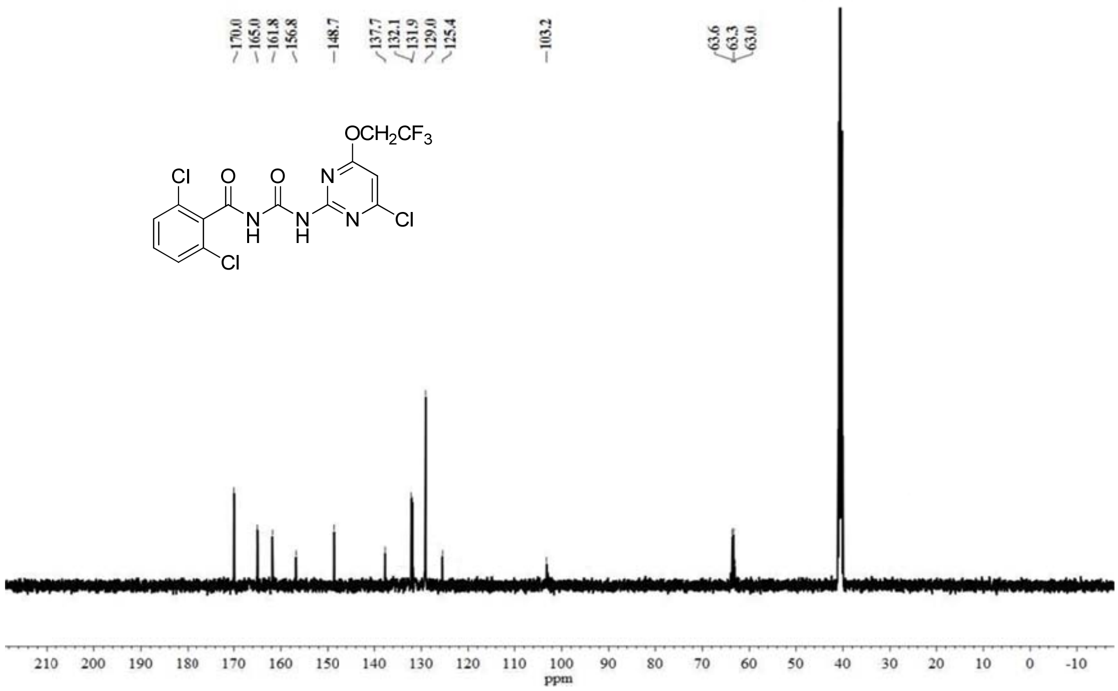

Figure S75 ^{13}C NMR spectra of compound 25

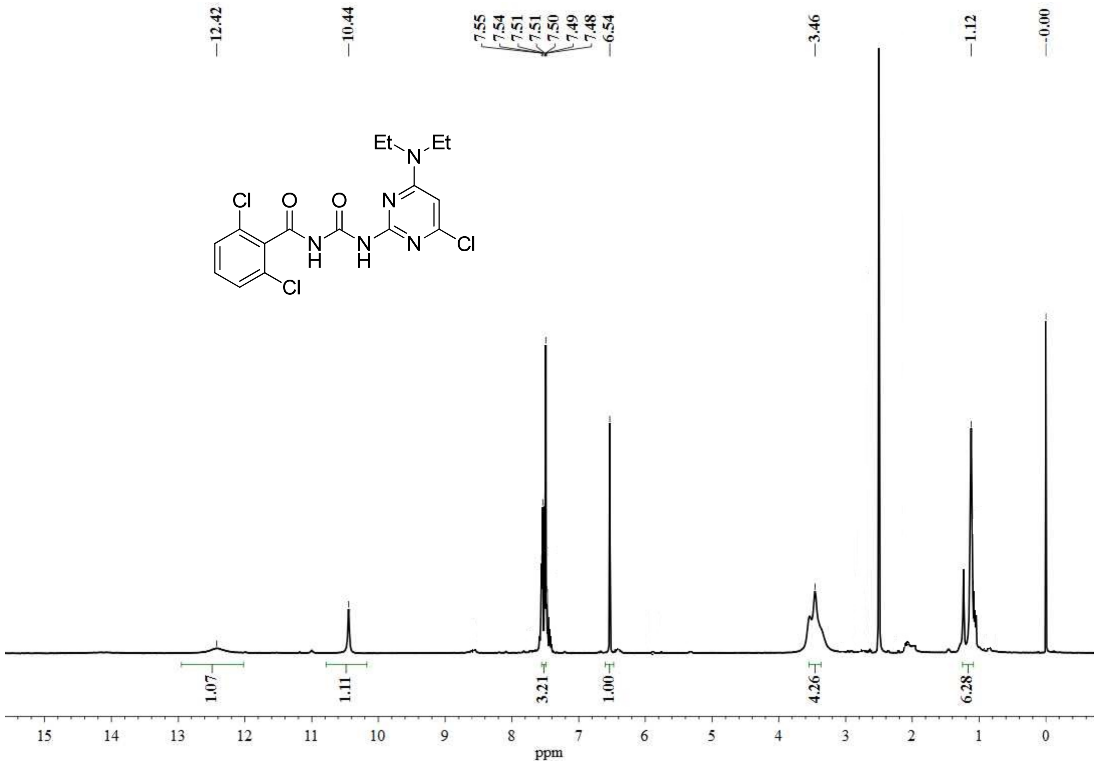

Figure S76 ^{1}H NMR spectra of compound 26

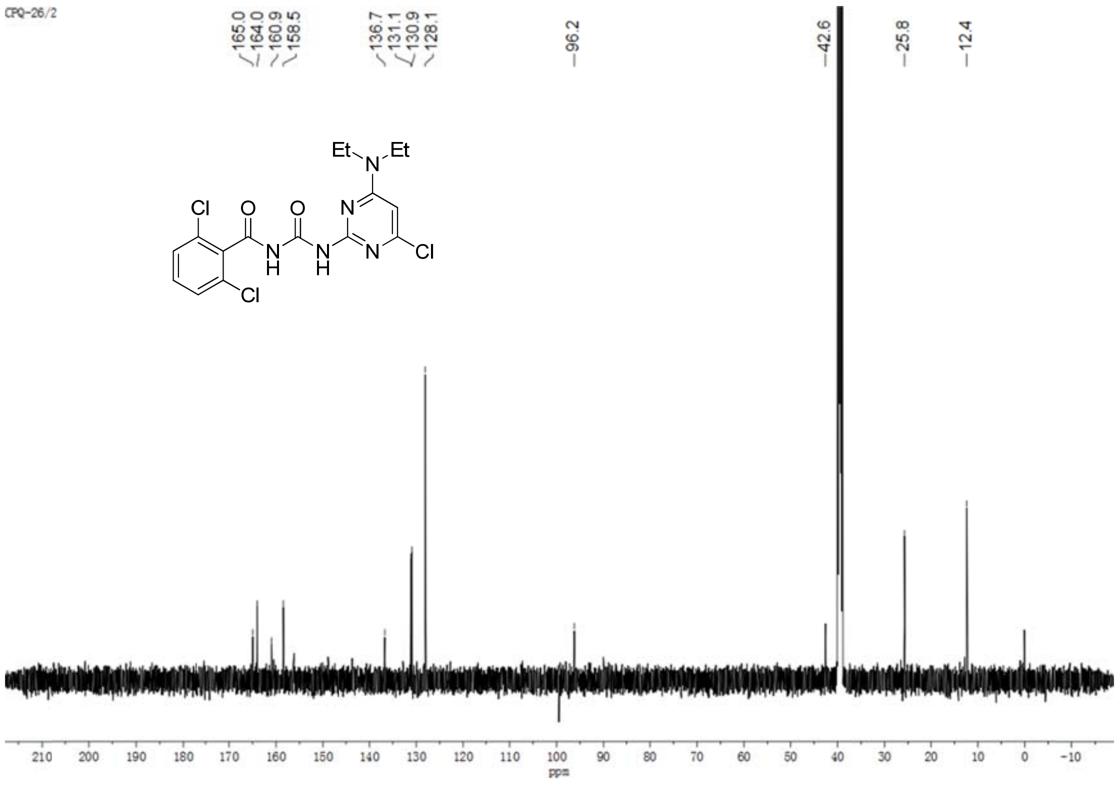

Figure S77 ^{13}C NMR spectra of compound 26

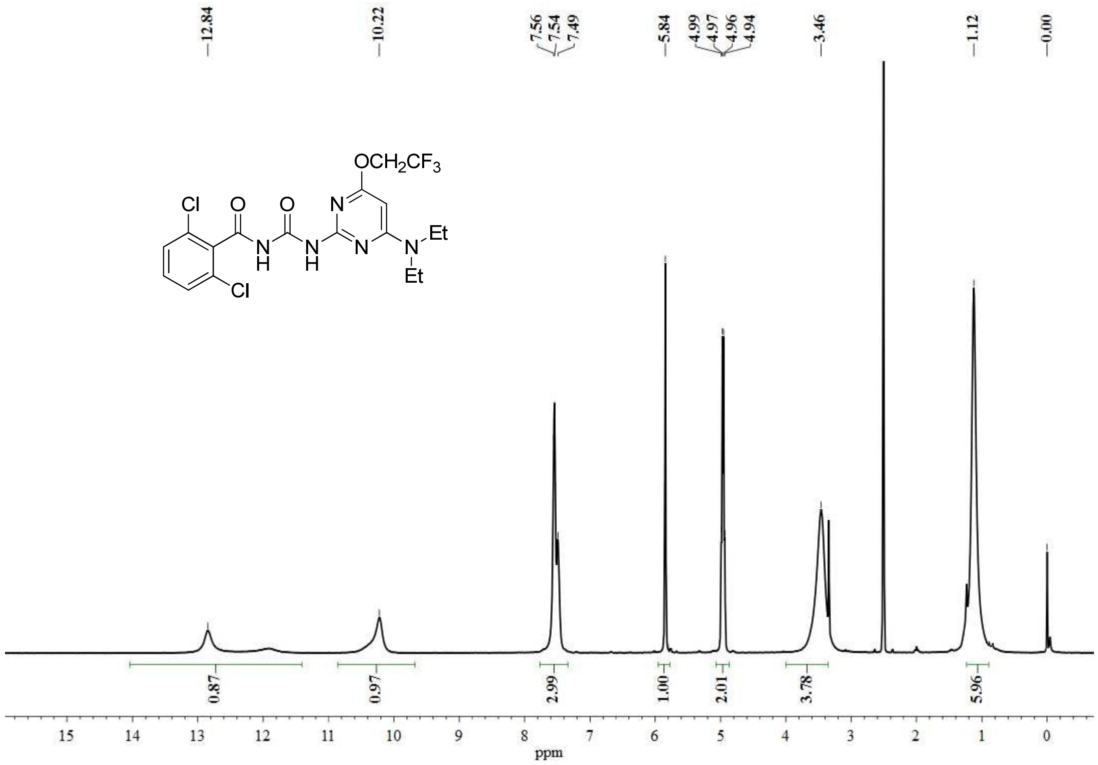

Figure S78 ^{1}H NMR spectra of compound 27

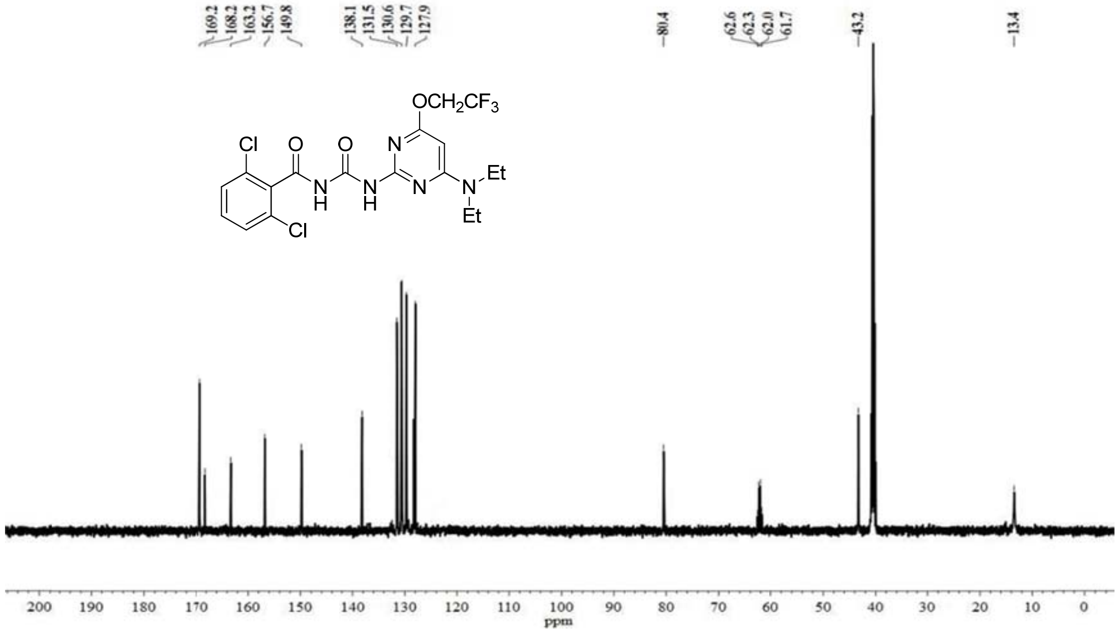

Figure S79 ^{13}C NMR spectra of compound 27

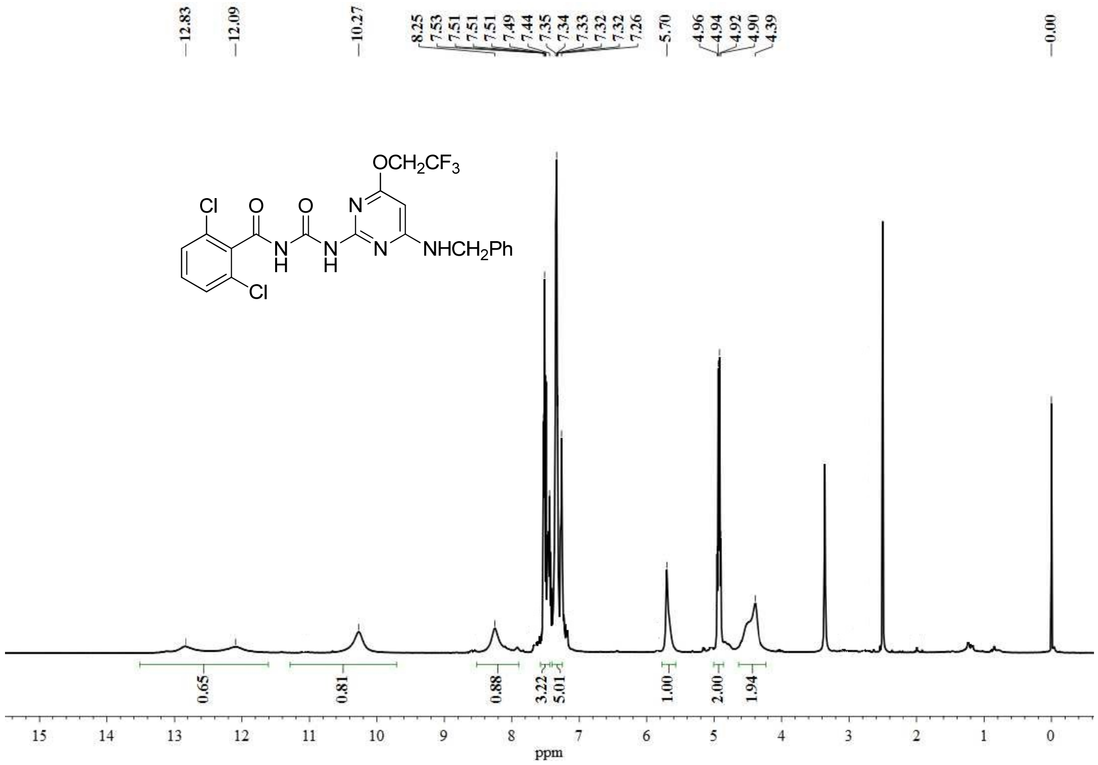

Figure S80 ^{1}H NMR spectra of compound 28

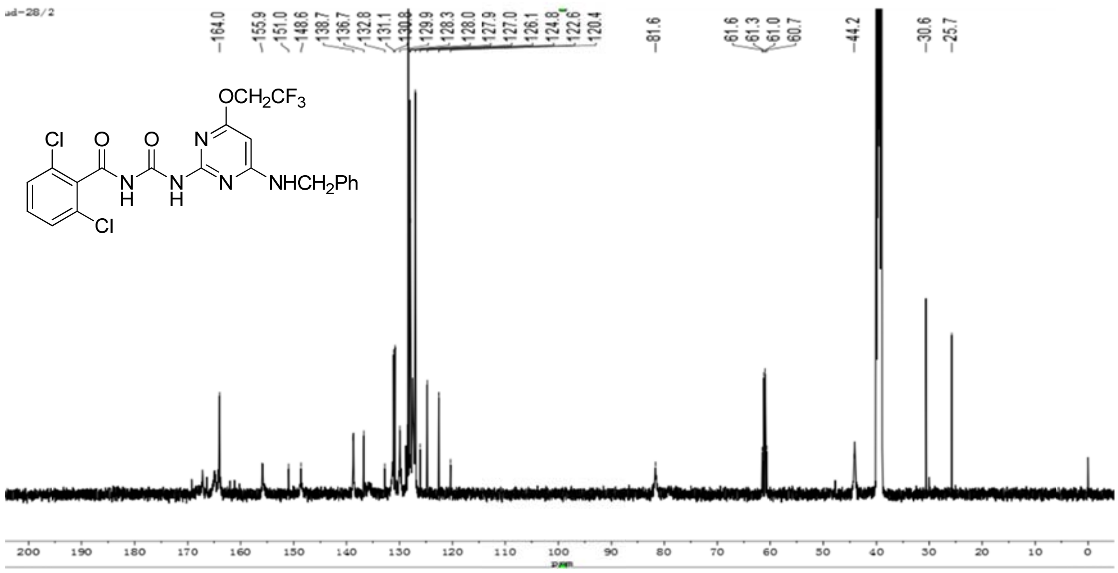

Figure S81 ^{13}C NMR spectra of compound 28

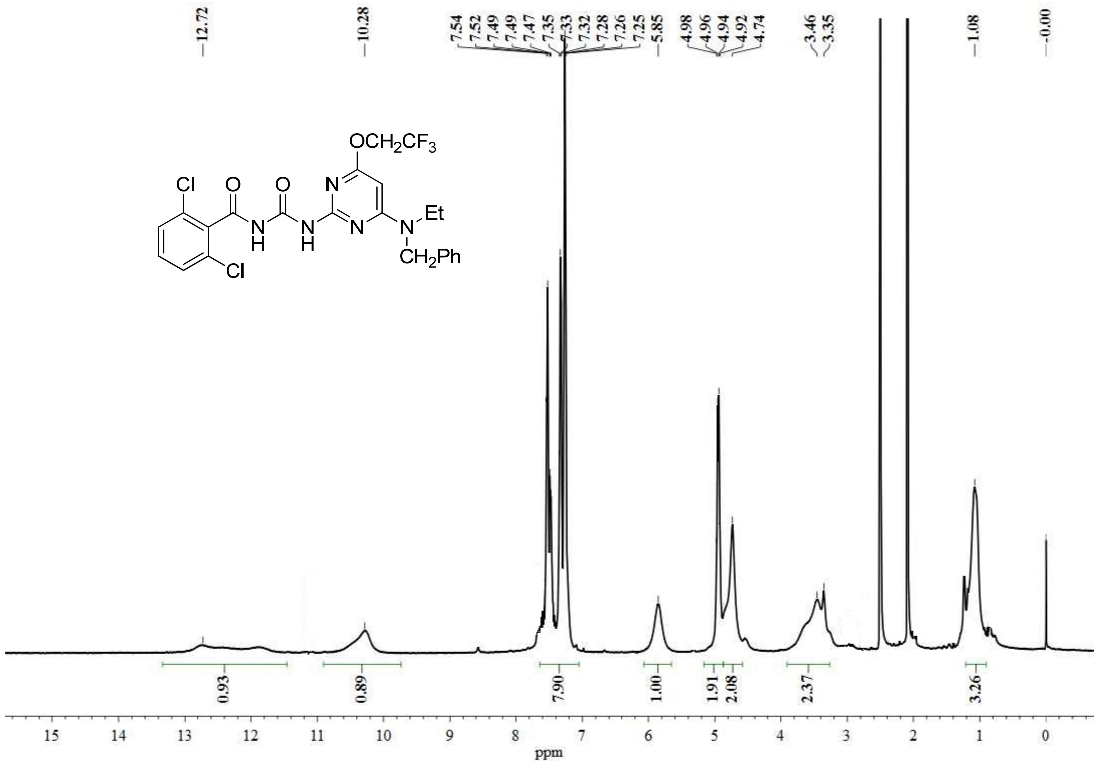

Figure S82 ^{1}H NMR spectra of compound 29

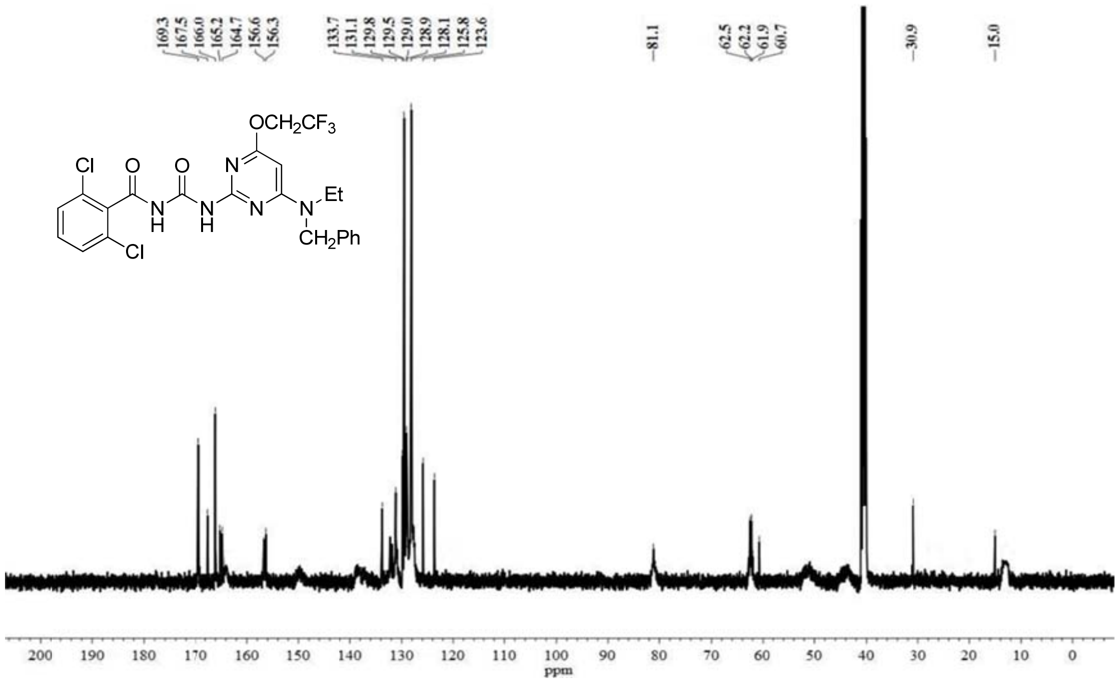

Figure S83 ^{13}C NMR spectra of compound 29

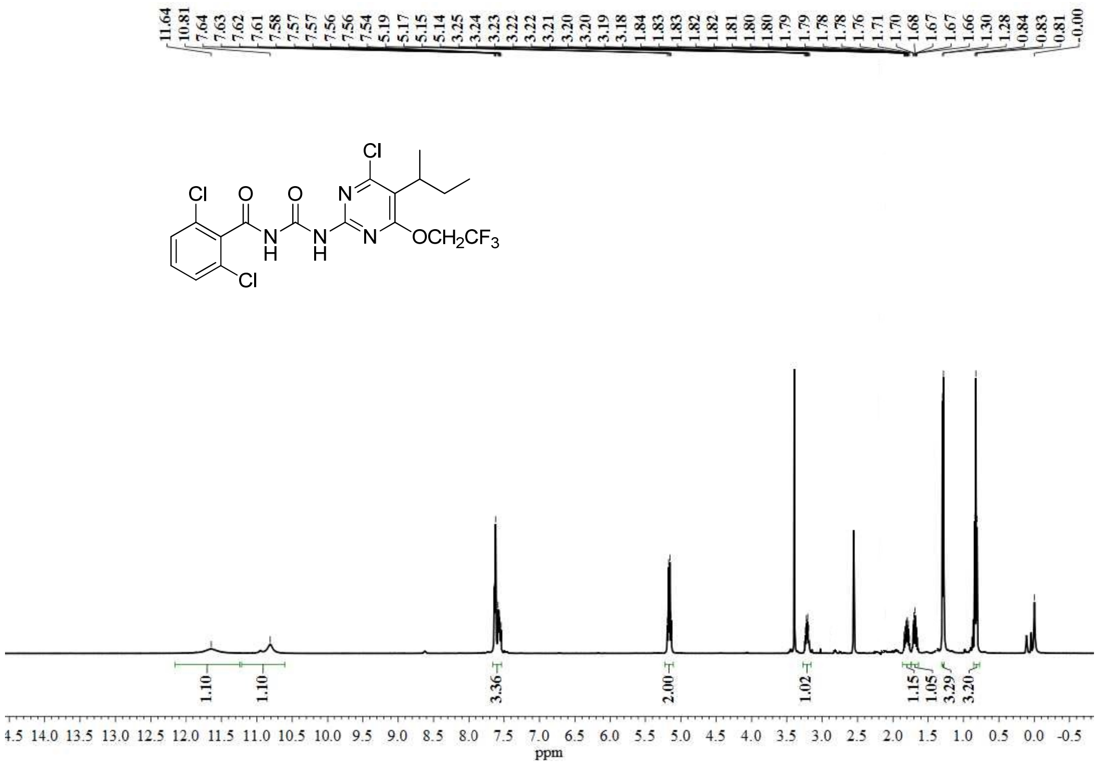

Figure S84 ^{1}H NMR spectra of compound 30

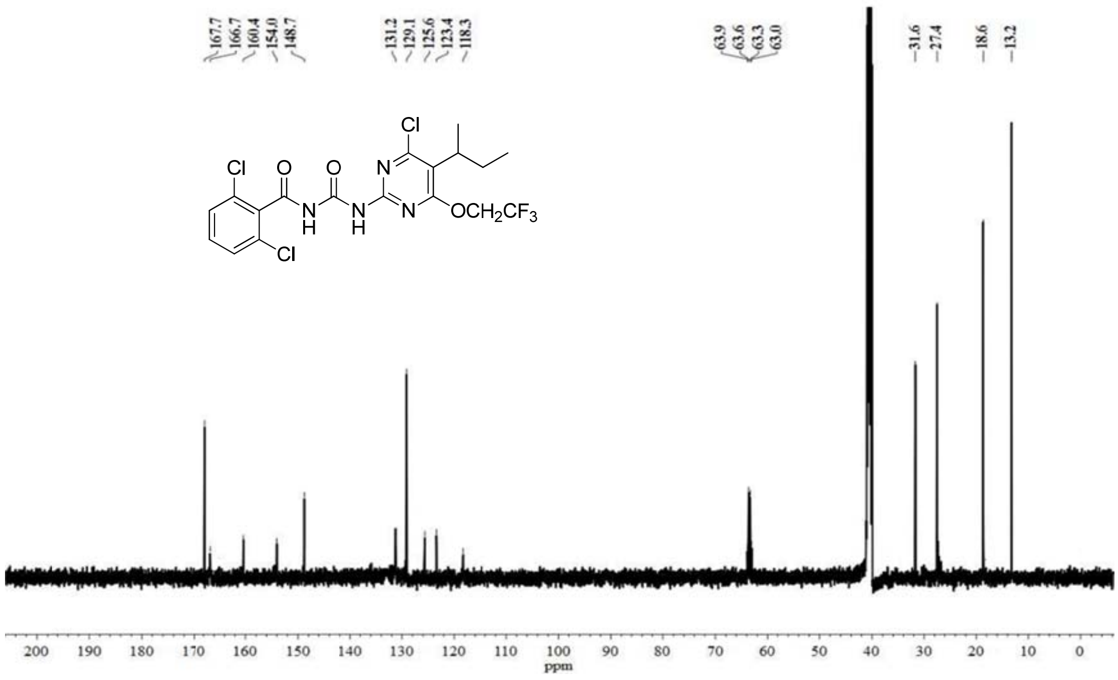

Figure S85 ^{13}C NMR spectra of compound 30

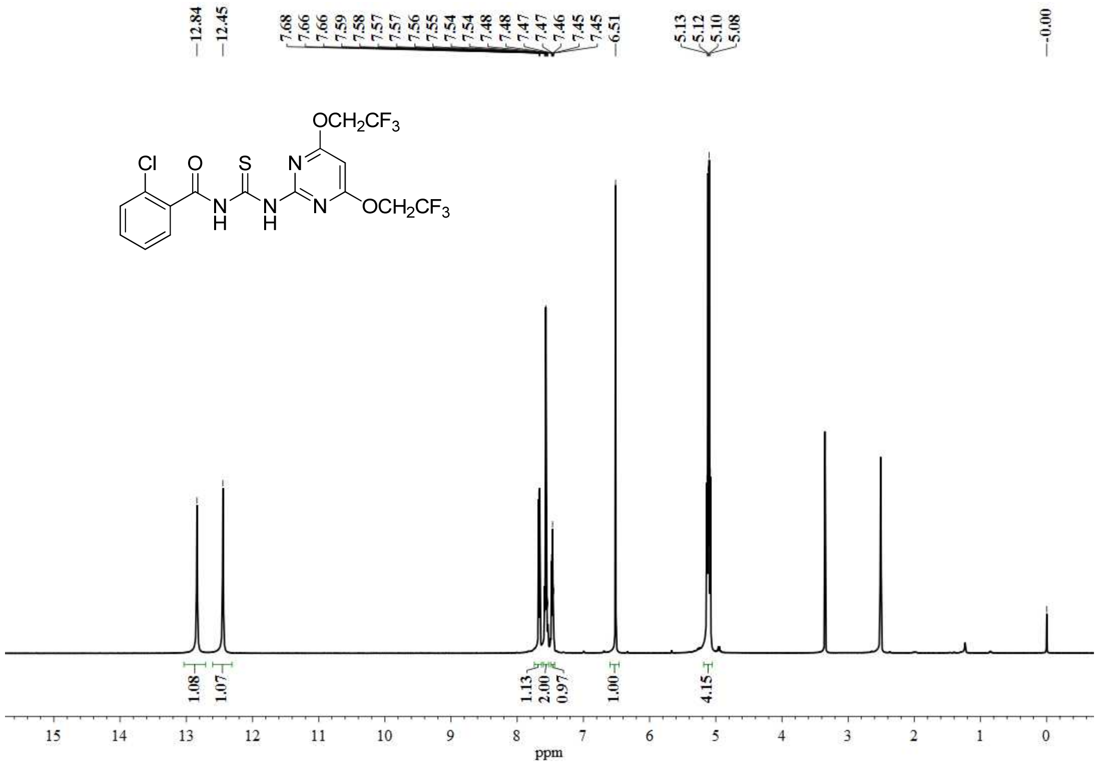

Figure S86 ^{1}H NMR spectra of compound 31

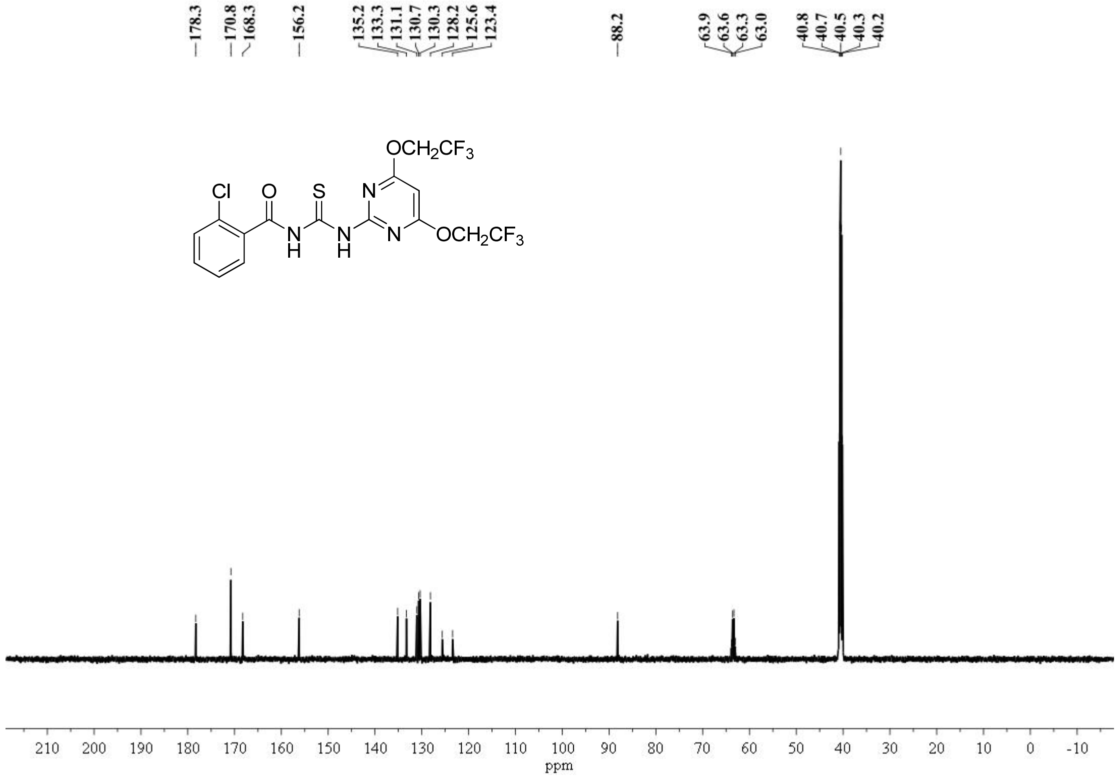

Figure S87 ^{13}C NMR spectra of compound 31

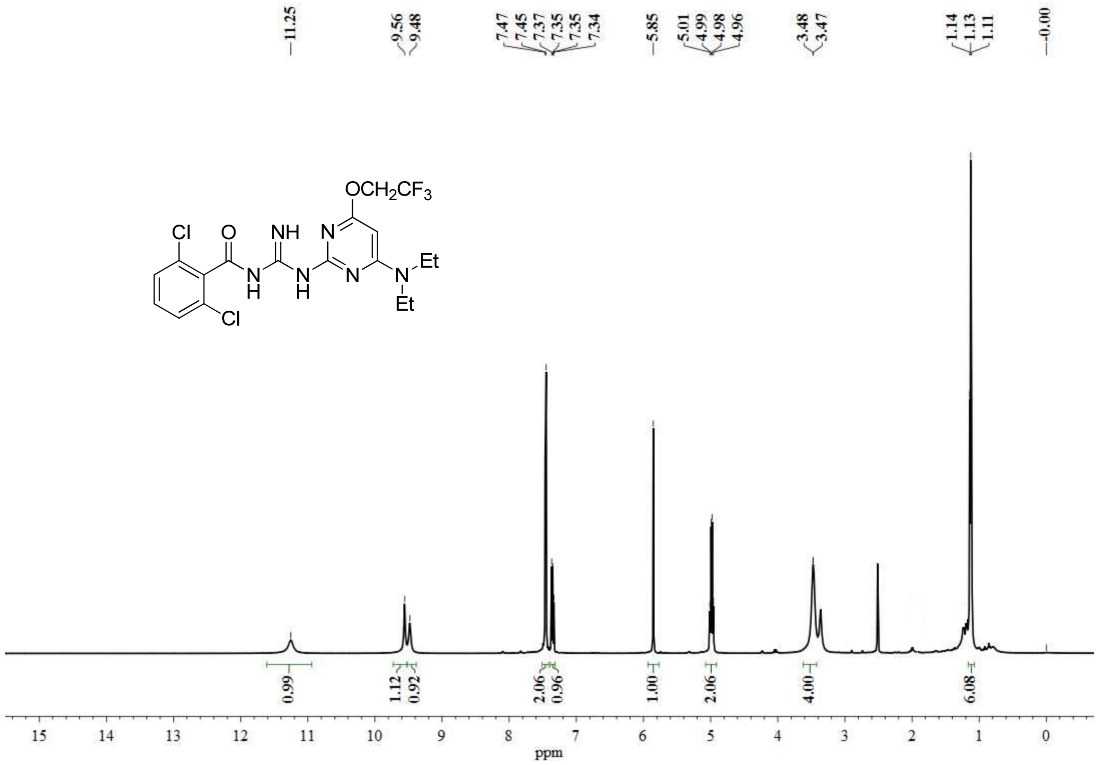

Figure S88 ^{1}H NMR spectra of compound 32

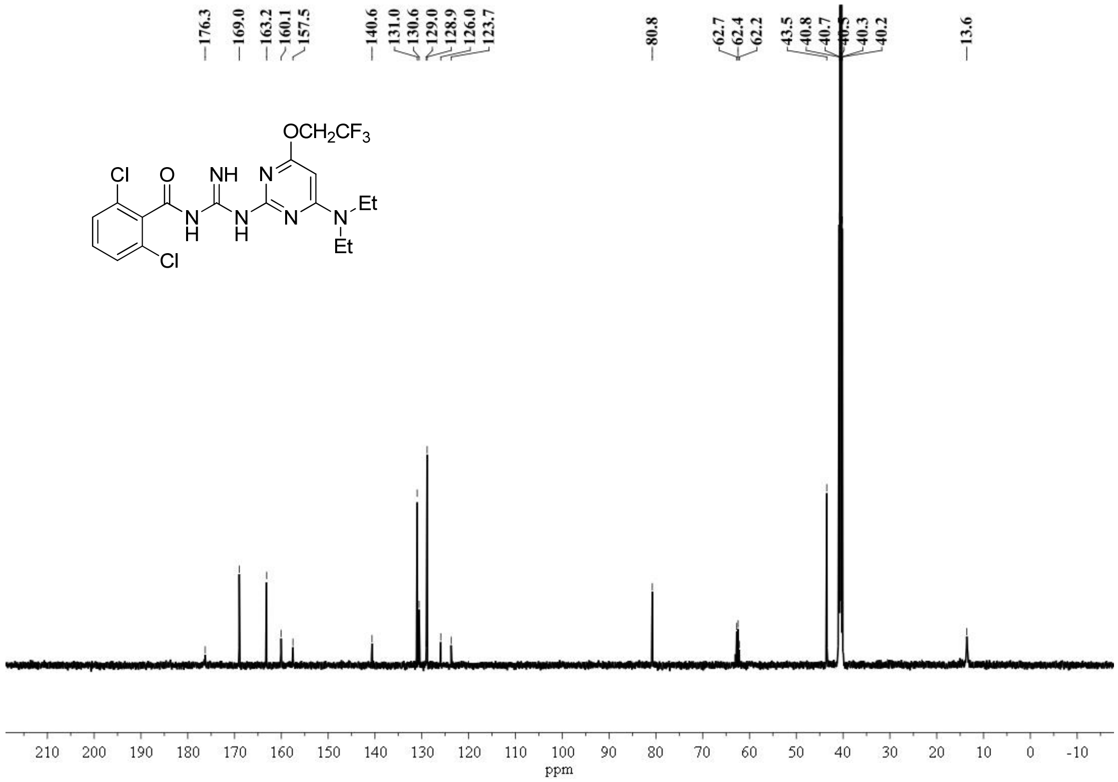

Figure S89 ^{13}C NMR spectra of compound 32

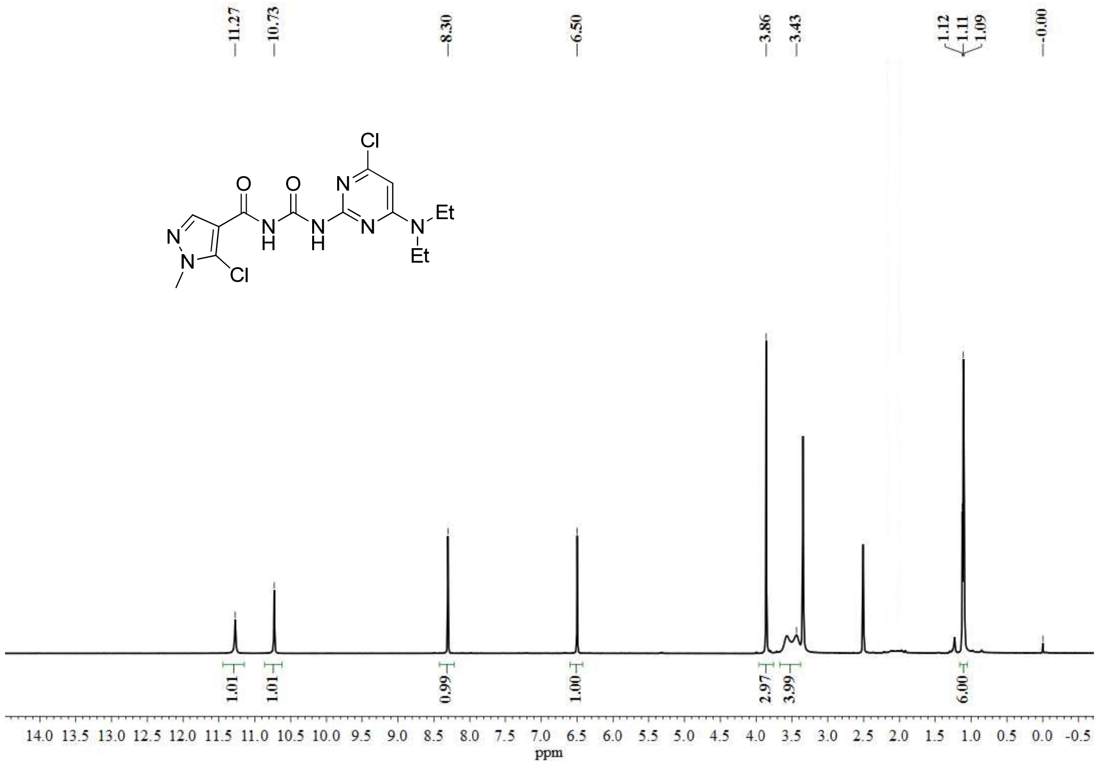

Figure S90 ^{1}H NMR spectra of compound 33

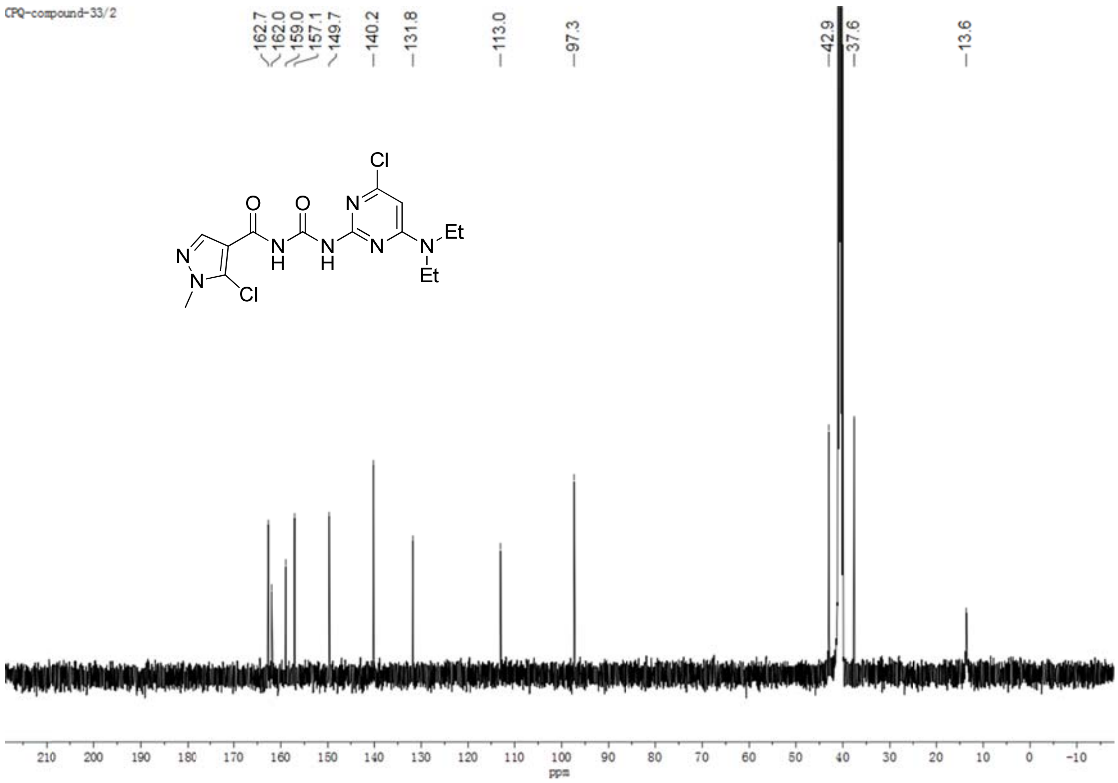

Figure S91 ^{13}C NMR spectra of compound 33

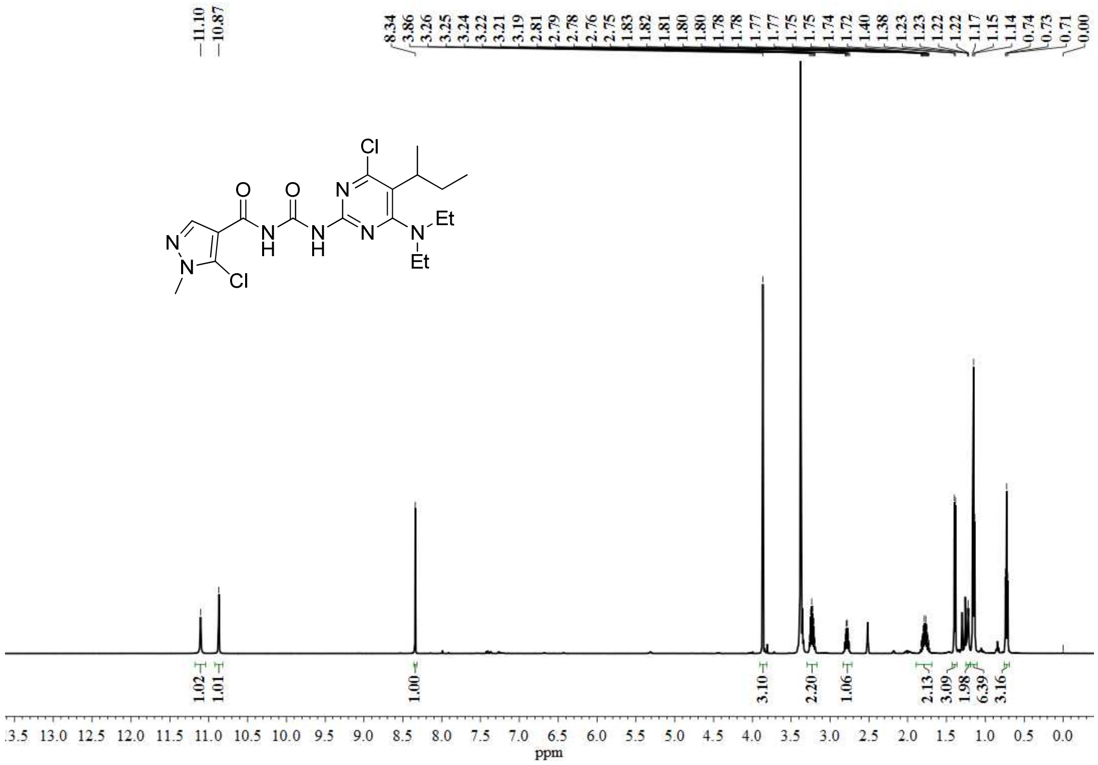

Figure S92 ^{1}H NMR spectra of compound 34

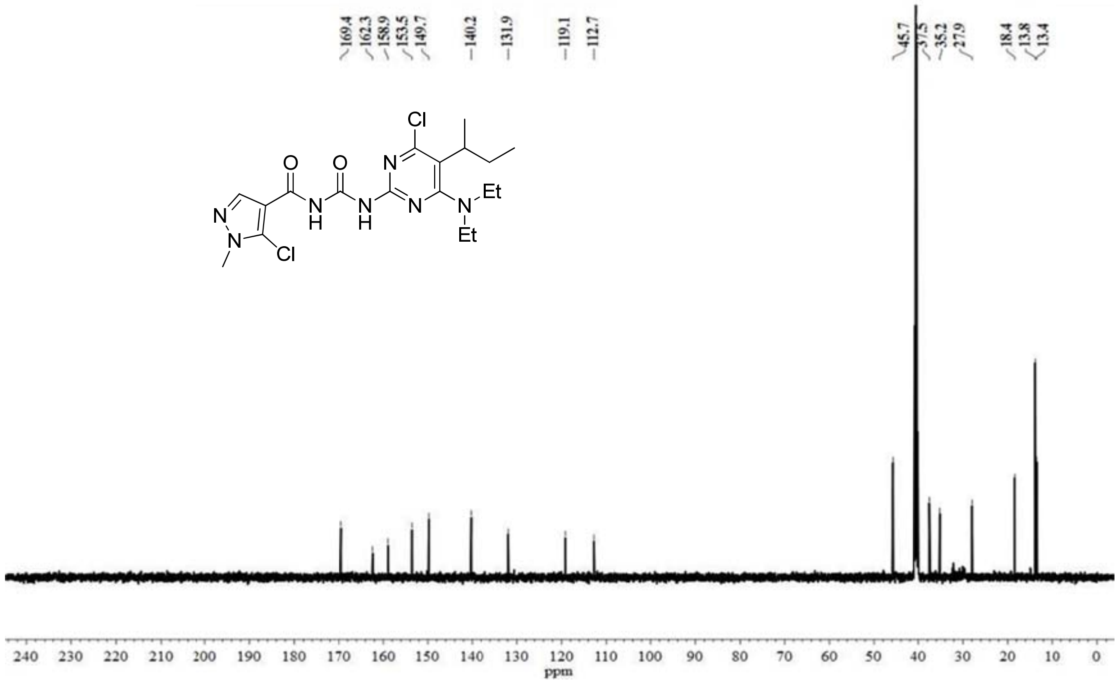

Figure S93 ^{13}C NMR spectra of compound 34

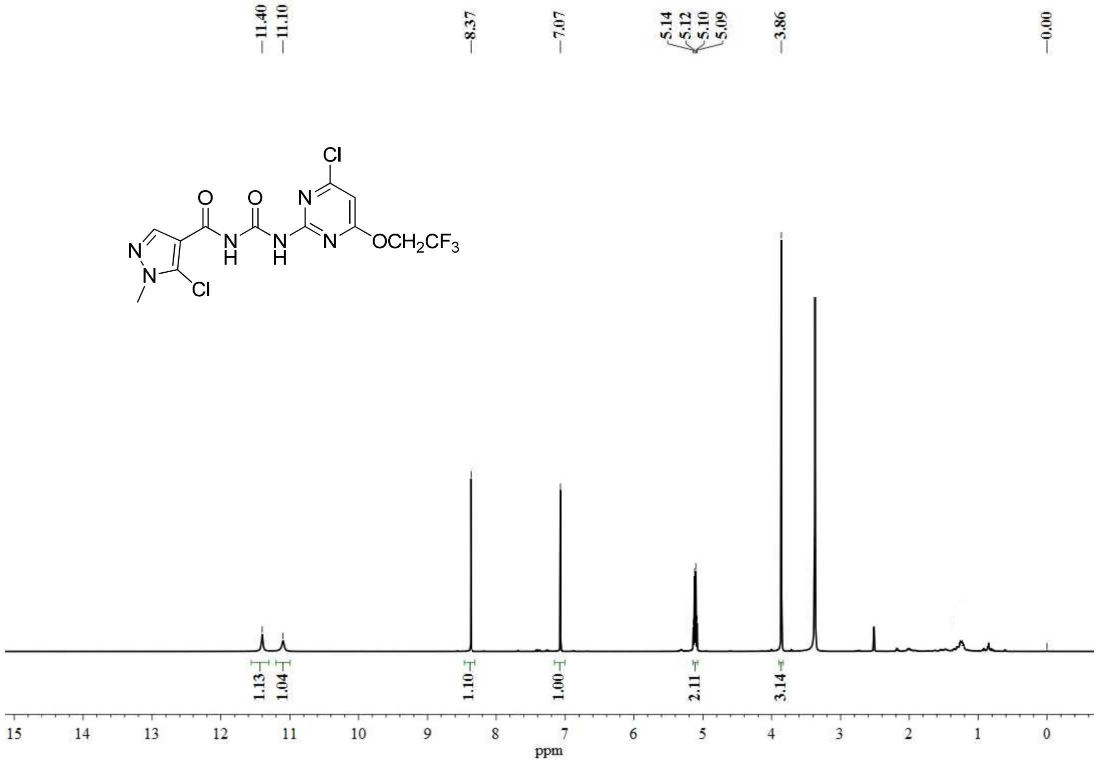

Figure S94 ^{1}H NMR spectra of compound 35

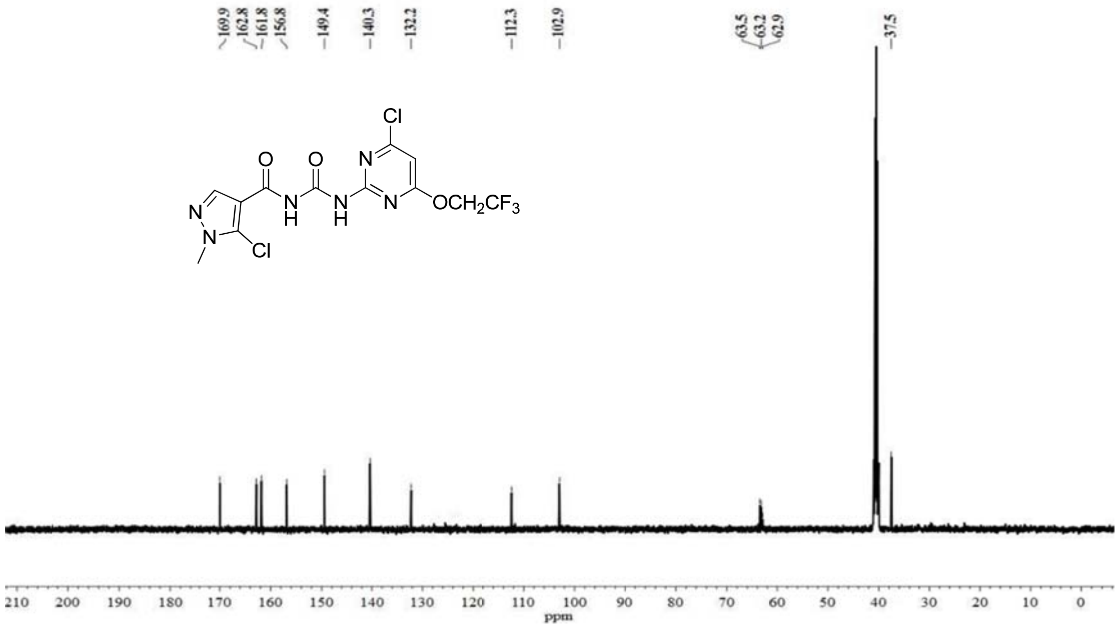

Figure S95 ^{13}C NMR spectra of compound 35

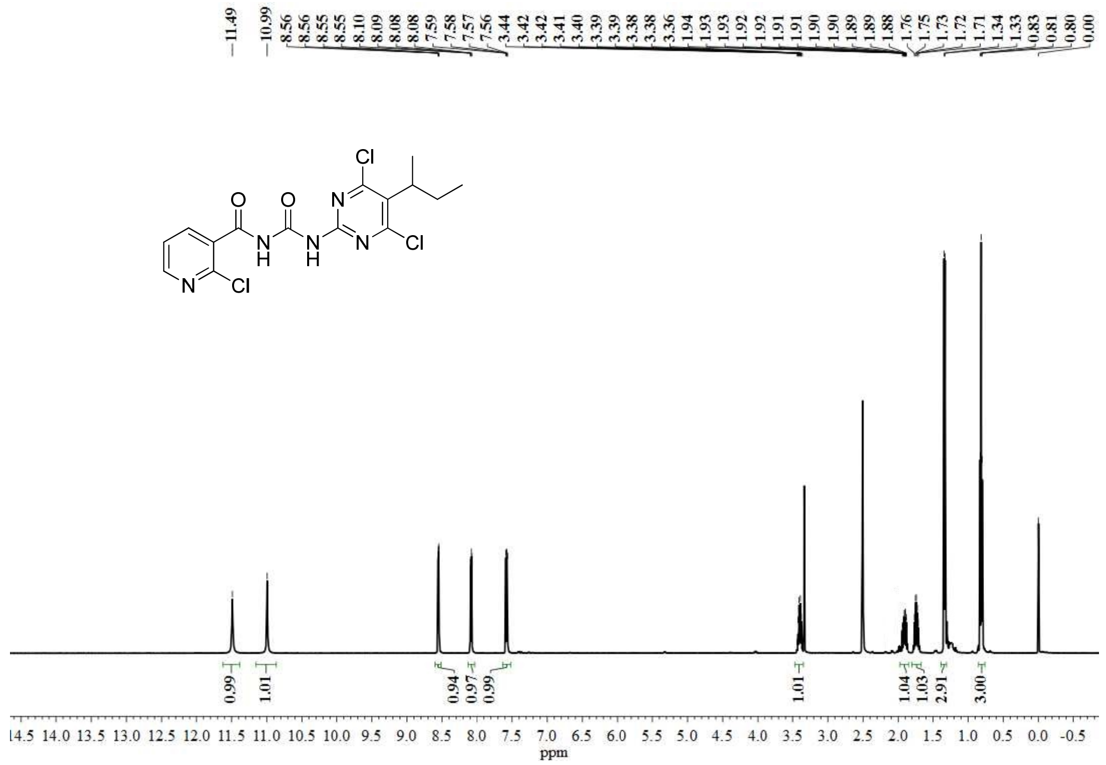

Figure S96 ^{1}H NMR spectra of compound 36

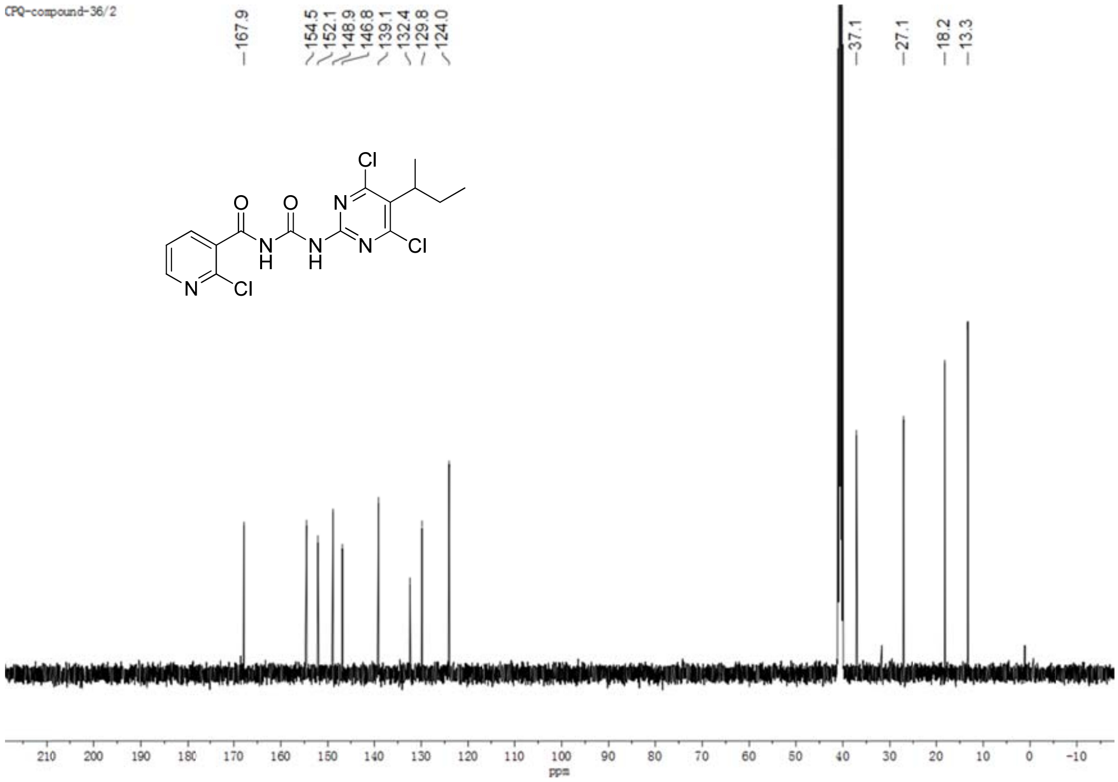

Figure S97 ^{13}C NMR spectra of compound 36

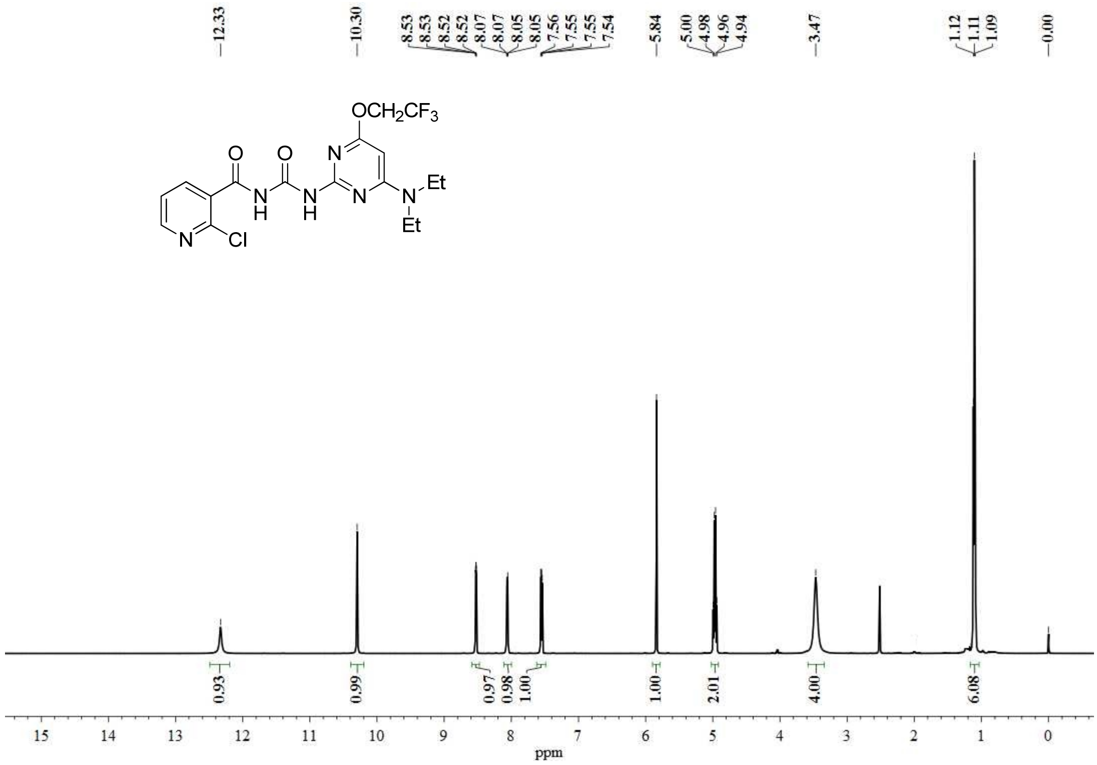

Figure S98 ^{1}H NMR spectra of compound 37

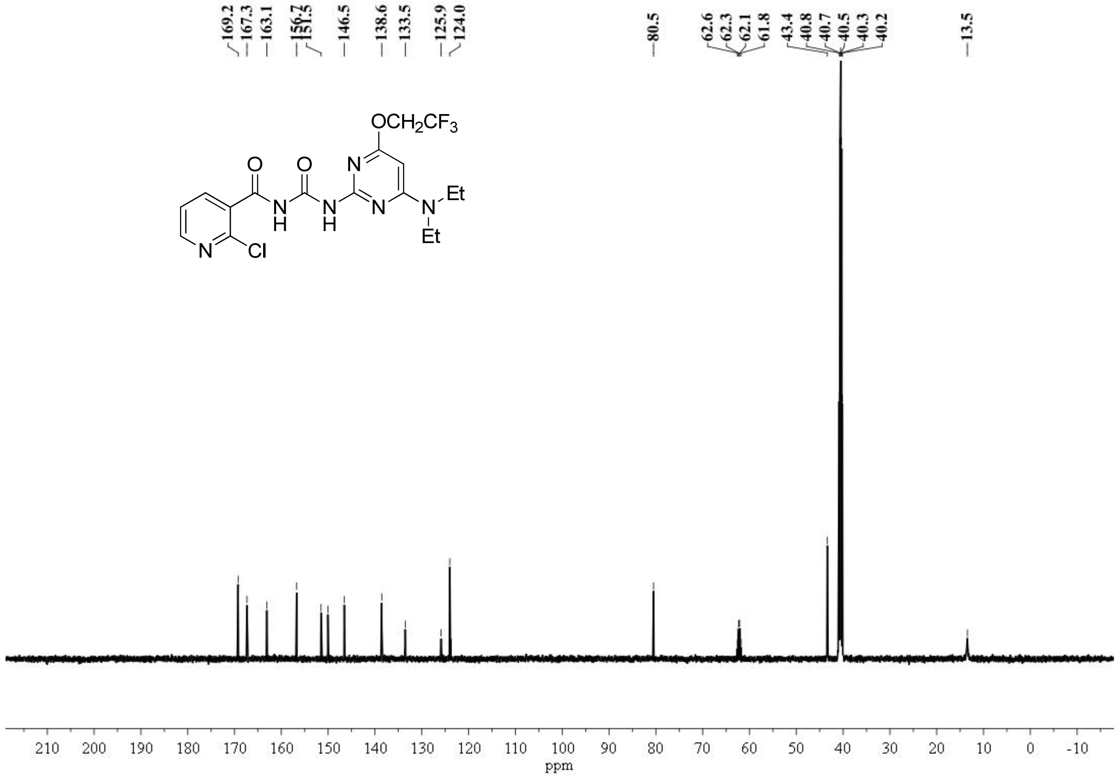

Figure S99 ^{13}C NMR spectra of compound 37

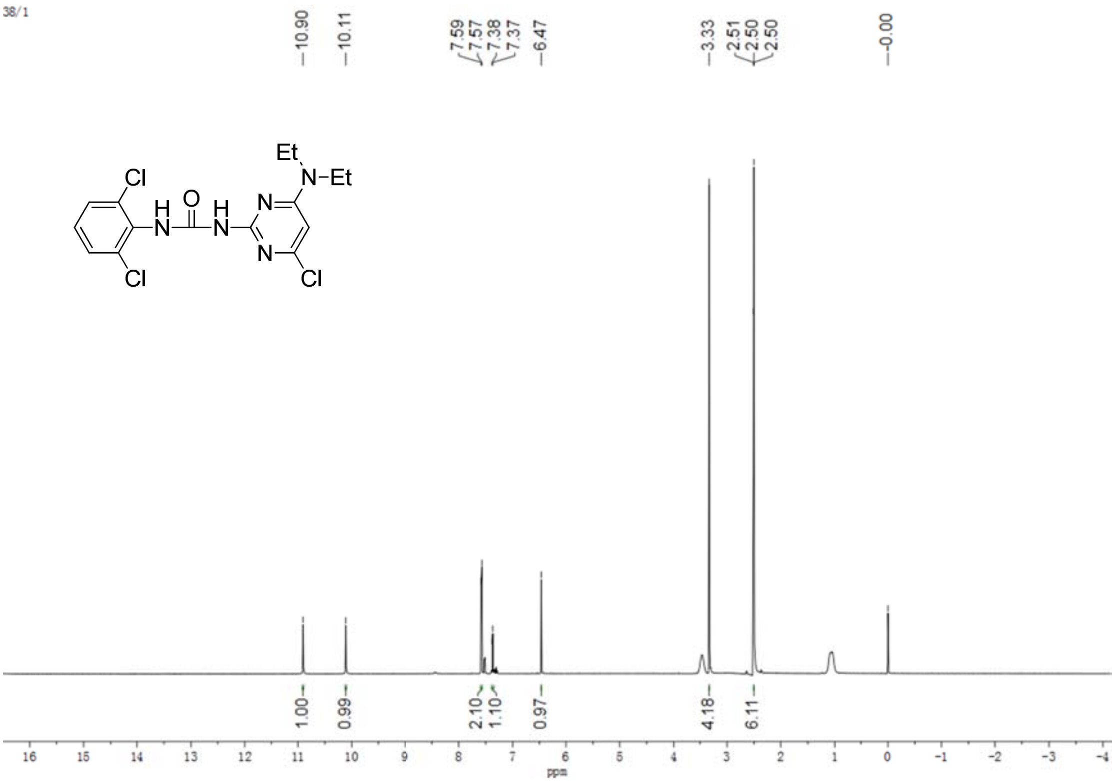

Figure S100 ^{1}H NMR spectra of compound 38

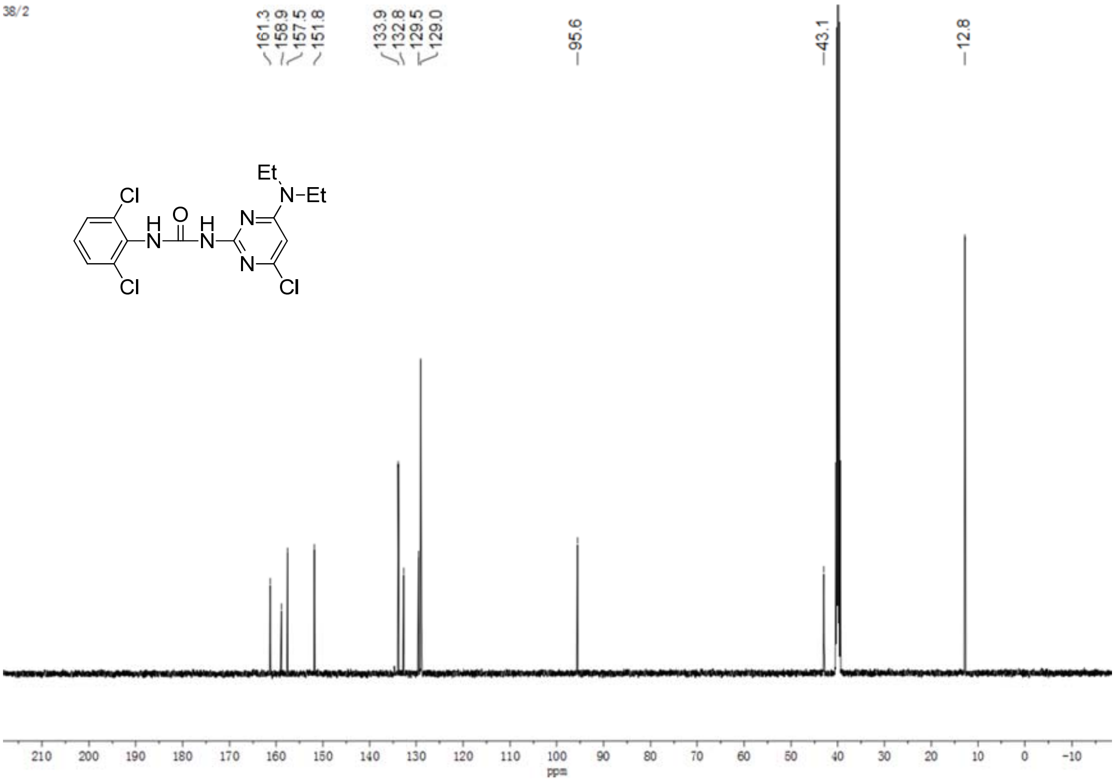

Figure S101 ^{13}C NMR spectra of compound 38

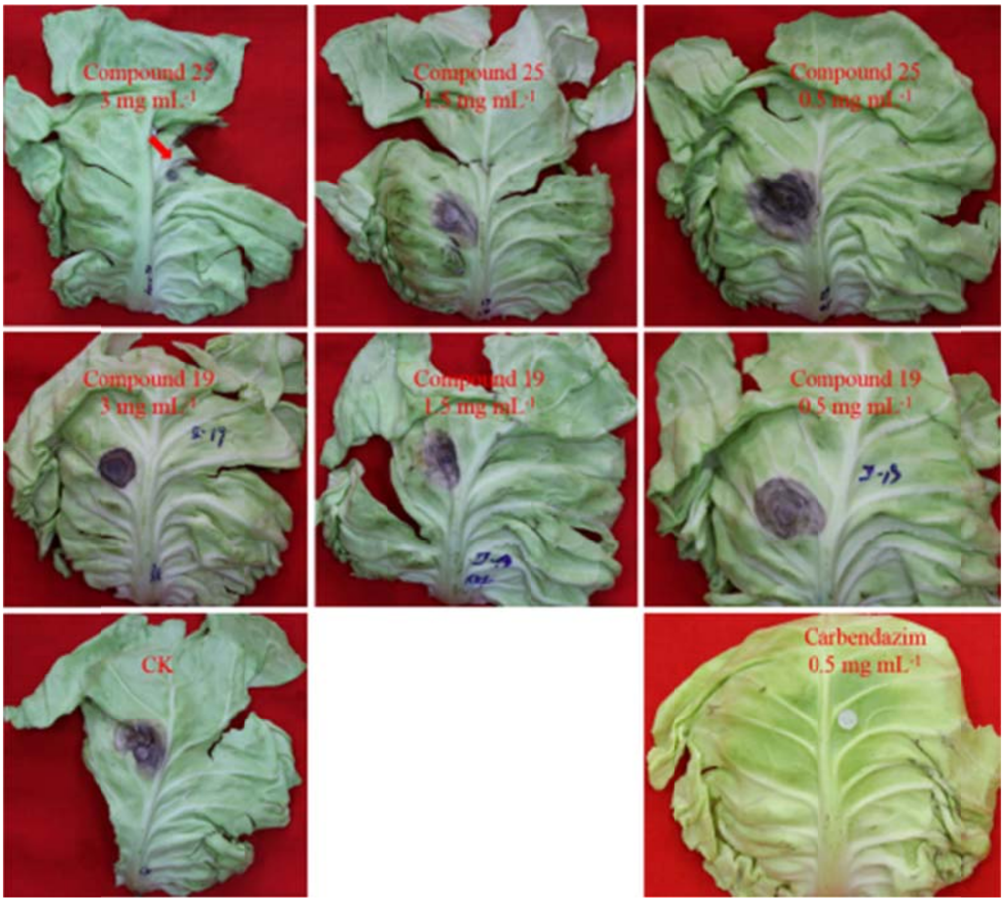

Figure S102 Photo for Protective activity of compounds
